# Cell Signaling by Tryptophan Catabolism

**DOI:** 10.1021/acs.biochem.6c00179

**Published:** 2026-04-24

**Authors:** Alex Torrelli-Diljohn, Bhargavi Kulkarni, Dario A. Vitturi

**Affiliations:** Department of Pathology, Heersink School of Medicine, 9968 The University of Alabama at Birmingham, Birmingham, Alabama 35233, United States

## Abstract

Tryptophan (Trp) metabolism follows three main branches: the kynurenine (KP), serotonin, and indole (IP) pathways. These pathways generate bioactive metabolites that regulate immune responses, redox balance, neurotransmission, metabolic homeostasis, inflammation, and circadian rhythms. A common theme across these pathways is the activation of the aryl hydrocarbon receptor (AhR). Several metabolites from KP, IP, and serotonin act as endogenous AhR ligands or interact indirectly with AhR, but the downstream consequences of this interaction depend on the cellular environment and inflammatory context. In addition, Trp metabolites also impact other signaling pathways, including GPR35, NMDA receptors, serotonergic receptors, and NAD^+^ biosynthesis. Notably, our group recently discovered that the upregulation of the KP results in the formation of the novel redox-active mediator kynurenine-carboxyketoalkene. This finding expands the signaling repertoire of Trp metabolism to include the modulation of cysteine-dependent pathways, with important implications for the maintenance of cellular homeostasis and immune control. Overall, flux through the oxidative arm of the KP links inflammation to cellular energy metabolism, while microbial indole derivatives influence host–mucosal immunity and host–microbe communication. The serotonin pathway connects neuroendocrine signaling with the peripheral nervous system’s regulation of metabolism. Shifts in Trp homeostasis caused by inflammation, alterations in microbial composition, or metabolic demand modify downstream signaling outputs under physiological and pathological conditions. In this regard, dysregulation of Trp metabolism is implicated in neurodegeneration, cancer, metabolic disease, cardiovascular dysfunction, and chronic inflammation. This review presents Trp catabolism as a distributed signaling network and offers new insights into its physiological functions.

## Graphical abstract

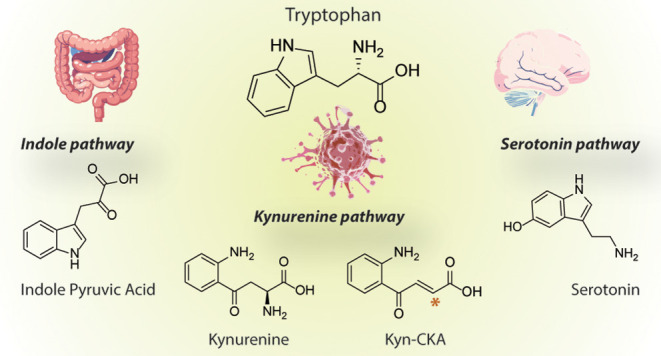

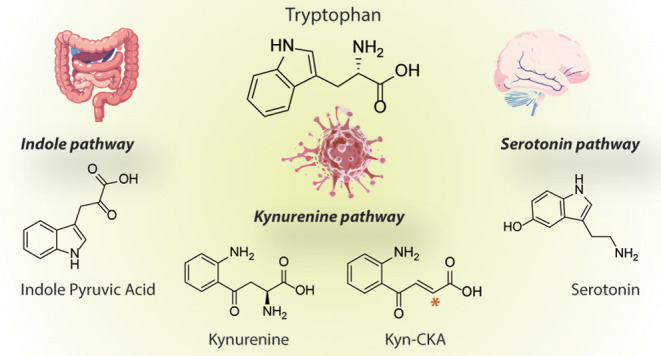

## Introduction

Tryptophan (Trp) is an essential amino acid obtained primarily through dietary sources.^1^ Trp also plays a crucial role in regulating key physiological processes through catabolism, mediated by its metabolites. Trp metabolism follows three pathways: the kynurenine pathway (KP), carried out by indoleamine-2,3-dioxygenases 1 and 2 (IDO1 and IDO2) or by tryptophan-2,3-dioxygenase 2 (TDO2);^2^ the indole pathway (IP) mediated primarily by the gut microbiota;^3^ and the 5-hydroxytryptamine (5-HT) or serotonin pathway, facilitated by tryptophan hydroxylase 1 and 2 (TPH1 and TPH2).^4^ Among these pathways, the KP is the primary route for Trp catabolism that produces metabolites that regulate immune tone, redox balance, inflammation, and NAD^+^ biosynthesis.^2^ Additionally, the microbial IP contributes to intestinal barrier integrity, host–microbe communication, immune regulation, and neuromodulation, and can either complement or counterbalance KP metabolites.^5–9^ In parallel, the serotonin pathway links Trp catabolism to neurotransmission, circadian regulation, neuroendocrine signaling, and the gut microbiome via the gut–brain axis.^10–12^ Historically, these pathways have been viewed in isolation; however, there is increasing evidence that they are interconnected and converge at shared signaling nodes.^13^ In this review, we offer a comprehensive overview of Trp catabolism, focusing on the signaling roles of its downstream metabolites and addressing disease relevance.

## The Tryptophan–Kynurenine Pathway

The kynurenine pathway (KP, Figure 1) is the primary route of tryptophan (Trp) catabolism in mammals, accounting for approximately 95% of systemic Trp degradation (Figure 1).^2^ This pathway is facilitated by three main enzymes: indoleamine-2,3-dioxygenases 1 and 2 (IDO1 and IDO2) or by tryptophan-2,3-dioxygenase 2 (TDO2). IDO1 expression is enriched in the placenta, pulmonary vascular epithelium, the brain, the female reproductive tract epithelium, pancreatic β-cells, and mature dendritic cells in secondary lymphoid organs.^14^ IDO1 is transcriptionally induced by proinflammatory cytokines such as IFN-γ and is upregulated during immune activation and metabolic stress.^15^ IDO2 is a homologue of IDO1 and is primarily expressed in hepatic and placental tissues; however, it has been shown to exhibit lower catalytic efficiency for Trp catabolism compared to IDO1.^16,17^ Finally, TDO2 is abundantly expressed in hepatocytes and transcriptionally regulated by glucocorticoids. In terms of post-translational mechanisms of regulation, TDO2 and IDO1 are regulated by heme availability and incorporation, as both enzymes are heme-dependent dioxygenases. In addition, IDO1 is regulated by phosphorylation and inhibited by nitric oxide, while the activity of TDO2 is modulated by tryptophan binding to exosites.^2,18–20^

**Figure 1:**
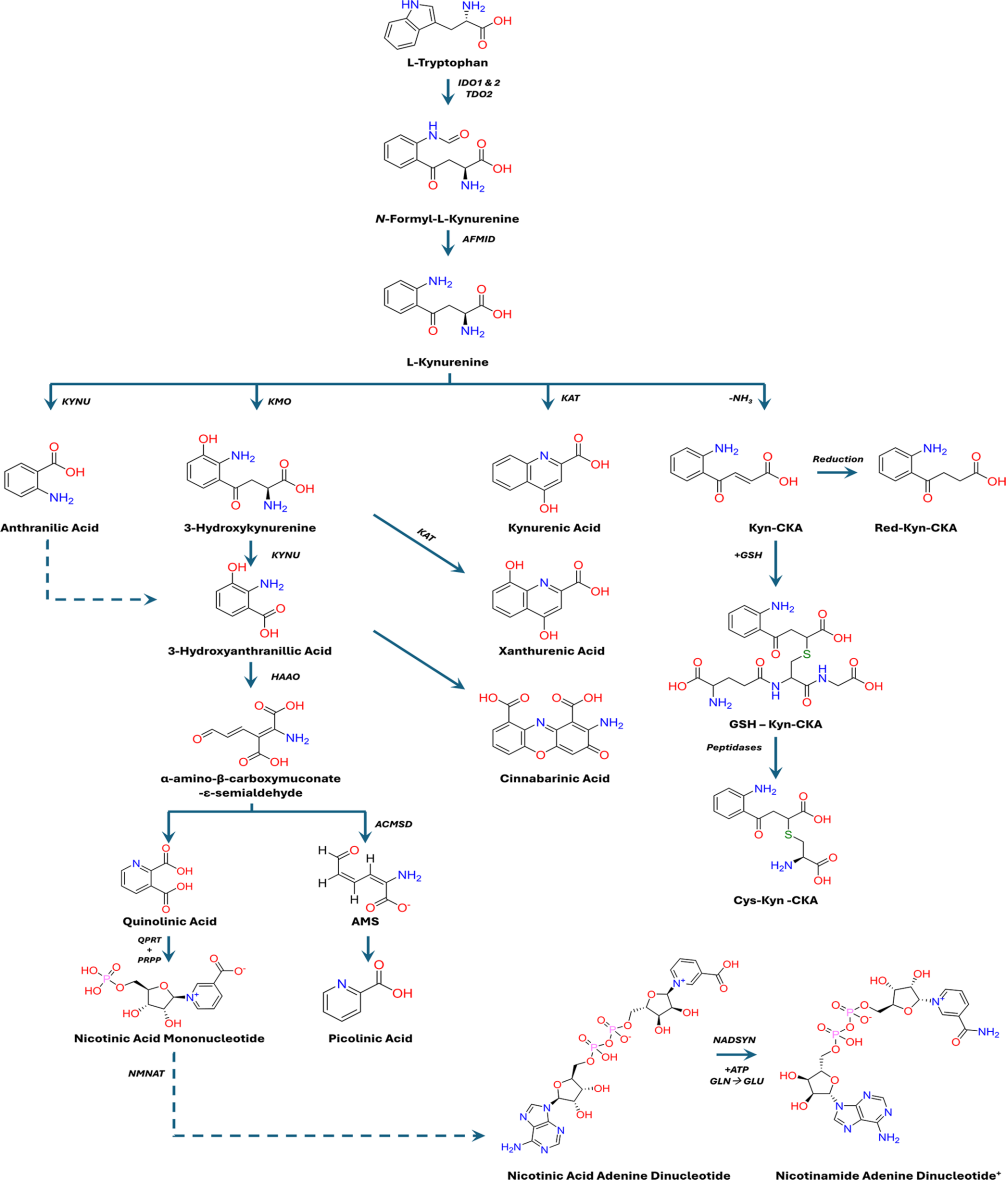
Overview of the kynurenine pathway of l-tryptophan catabolism. l-Tryptophan is oxidized by indoleamine-2,3-dioxygenases (IDO1/2) or tryptophan-2,3-dioxygenase (TDO2) to form *N*-formyl-l-kynurenine (NFK). NFK is hydrolyzed by arylformamidase (AFMID) to l-kynurenine (KYN), the first stable metabolite and a central metabolic branch point in the KP. KYN is metabolized by kynureninase (KYNU), kynurenine 3-monooxygenase (KMO), or kynurenine aminotransferases (KATs) to form anthranilic acid (AA), 3-hydroxykynurenine (3-HK), or kynurenic acid (KynA), as well as spontaneously deaminate to form the electrophilic metabolite kynurenine-carboxyketoalkene (Kyn-CKA). 3-HK is metabolized to 3-hydroxyanthranilic acid (3-HAA) by KYNU or by KATs to form xanthurenic acid (XA). 3-HAA can either be metabolized by 3-hydroxyanthranilate 3,4-dioxygenase (HAAO) to form α-amino-β-carboxymuconate-ε-semialdehyde (ACMS) or undergo oxidative dimerization to form cinnabarinic acid (CA). ACMS serves as a branch point intermediate that can either undergo spontaneous intramolecular cyclization to quinolinic acid (QA), or it can be metabolized by ACMS decarboxylase (ACMSD) to form picolinic acid (PA). QA is converted to nicotinic acid mononucleotide (NAMN) by quinolinate phosphoribosyltransferase (QPRT), which is subsequently adenylated by nicotinamide mononucleotide adenyltransferases (NMNAT) to generate nicotinic acid adenine dinucleotide (NAAD). Finally, NAAD is amidated by nicotinamide adenine dinucleotide synthetase (NADSYN) to form NAD^+^. Kyn-CKA is metabolized by the mercapturic acid pathway to generate glutathione (GSH) and cysteine (Cys) conjugates or reduced to Red-Kyn-CKA by an unidentified NADPH-dependent reductase.

Tryptophan binding to the active site of IDO1/2 or TDO2 enables heme-bound oxygen (O_2_) to cleave the 2,3-double bond of Trp. This reaction forms a transient 2-indolenylperoxo radical that yields ferryl-oxo (Fe^IV^=O) and Trp-epoxide intermediates. The second step of the reaction occurs with the protonation of the Trp-epoxide intermediate, which allows a nucleophilic attack by the ferryl-oxygen at C2, cleaving the C2–C3 bond to produce *N*-formylkynurenine (NFK).^21,22^ NFK is subsequently hydrolyzed by arylformamidase (AFMID) into l-kynurenine (KYN), which is the first stable product of the KP.^23^ AFMID is systemically expressed with significant levels in the liver, kidneys, CNS, and endocrine tissues, as well as in the gastrointestinal and reproductive systems, muscular and vascular tissues.^17^

### Kynurenine

The formation of KYN represents a branch point for four downstream pathways that lead to the formation of kynurenic acid (KynA), anthranilic acid (AA), 3-hydroxykynurenine (3-HK), and the recently identified cysteine-reactive metabolite kynurenine-carboxyketoalkene (Kyn-CKA).^23,24^

Under homeostatic conditions, KYN is produced by TDO2 in the liver and can also be obtained from various dietary sources, such as honey, mushrooms, milk, and fermented foods.^25^ However, under inflammatory conditions or in the setting of cancer, KYN synthesis is predominantly driven by IDO1, which is expressed in dendritic cells, macrophages, endothelial cells, and epithelial barriers.^14^ Systemic and local KYN levels are controlled not only by tissue- and context-specific changes in enzyme expression but also by the activity of different transporters in the cellular membrane. In this regard, KYN is transported across the blood–brain barrier using the l-type amino acid transporter 1 (LAT1, SLC7A5) and organic anion transporters 1 (OAT1, SLC22A6) and 3 (OAT3, SLC22A8), which account for 60% of KYN that enters the brain.^26^ In astrocytes and T cells, KYN uptake also involves LAT1, but contributions from LAT2 (SLC7A8) and PAT4 (SLC36A4) have also been reported.^27–29^ Moreover, a role for the cystine/glutamate antiporter (xCT, SLC7A11) in KYN uptake was recently identified in cancer cells.^30^

#### Kynurenine–AhR Signaling

In addition to its role as a hub metabolite in tryptophan metabolism, KYN has been reported to function as an endogenous activator of the cytosolic aryl hydrocarbon receptor (AhR).^2^ The AhR protein–receptor complex contains c-Src, heat shock protein Hsp90, and two cochaperones, XAP2 and p23.^31–34^ Upon ligand binding, AhR undergoes a conformational change that exposes a nuclear localization signal for nuclear transport, where it detaches from its chaperones and heterodimerizes with the AhR nuclear translocator protein (ARNT).^35,36^ The AhR/ARNT heterodimer functions as a transcription factor, binding to xenobiotic response elements to drive transcription of xenobiotic-metabolizing enzymes and drug transporters, including *CYP1A1*, *CYP1A2*, *CYP1B1*, and other members of the cytochrome P450 family.^37^ Additionally, AhR plays crucial roles in regulating immune responses,^38–44^ fatty acid metabolism,^45,46^ epithelial cell barrier integrity,^47,48^ and cell migration.^49^ Notably, while the upregulation of the KP is often associated with AhR activation, whether KYN itself acts as the proximal ligand is controversial. Recently obtained cryo-EM and X-ray diffraction structures for AhR–Hsp90–XAP2 and AhR–ARNT–DNA complexes identified a conserved binding site capable of accommodating a wide range of hydrophobic planar ligands; however, none of these studies included kynurenine.^50–52^ In this regard, although the absence of structural data does not prove the inability of kynurenine to be a *bona fide* AhR ligand, recent molecular docking studies strongly argue against this possibility.^53,54^ As a result, the emerging view in the field is that kynurenine acts as a precursor for secondary products or metabolites that can bind and activate AhR with higher potency.^55^ Examples of these species include kynurenic acid, the electrophilic derivative kynurenine-carboxyketoalkene (Kyn-CKA), and the trace-extended aromatic condensation products (TEACOPs).^24,53,54,56^

#### The Kynurenine–AhR Axis in Immune Regulation

Tryptophan metabolism via the KP directly influences immune regulation via AhR signaling.^38^ In this regard, a seminal study by Mezrich et al. demonstrated that activation of the KYN–AhR axis leads to AhR-dependent generation of FoxP3^+^ T regulatory cells (Tregs).^57^ The generation of KYN-dependent FoxP3^+^ Tregs promotes an immunosuppressive microenvironment that inhibits the production and activation of effector T cells. Furthermore, the activation of toll-like receptors (TLR4 by lipopolysaccharide and TLR9 by CpG) induces AhR expression in bone-marrow-derived dendritic cells, which in turn promotes IDO1 expression and subsequent KYN production. Notably, deletion of AhR in dendritic cells inhibits IDO1 expression, reduces extracellular KYN accumulation, and results in both impaired IL-10 production and increased polarization of naïve T cells toward the T-helper-17 (Th17) phenotype.^58^ These findings suggest that KYN–AhR signaling functions as a positive feedback loop in which AhR activation promotes further KYN production and the engagement of immunosuppressive signaling. More recently, this mechanism has been shown to have critical implications for the response to infection and the maintenance of peripheral self-tolerance.^59,60^

In the context of cancer, overexpression of both IDO1 and TDO2 enables constitutive KYN–AhR signaling, which results in the generation of a sustained immunosuppressive tumor microenvironment.^61–63^ More specifically, KYN–AhR signaling enhances FoxP3^+^ stability and promotes the expression of IL-10, cytotoxic T-lymphocyte antigen 4 (CTLA-4), and vascular endothelial growth factor (VEGF). This results in a shift in tumor-associated macrophages toward an M2-like state characterized by elevated CD206 and PD-L1 expression, reduced antigen presentation, and impaired CD8^+^ T-cell activation, thus contributing to tumor cell immune evasion.^61,62^

#### Kynurenine Signaling in Cardiometabolic Diseases

The presence of increased systemic levels of KYN in obesity has been reported to contribute to the onset of metabolic dysfunction.^64–67^ In this regard, and in line with previous observations, Huang et al. demonstrated that the white adipose tissue is a major source of circulating KYN due to increased IDO1 expression, both in high-fat diet models and in volunteers with increasing body mass indices.^65,68^ Mechanistically, KYN was observed to activate the STAT3/IL-6 pathway, which disrupts lipid homeostasis in adipocytes, resulting in impaired insulin signaling and glucose tolerance.^68^ Besides the adipose tissue, obesity has been associated with a shift in intestinal metabolism, redirecting Trp from microbiome-dependent indole synthesis toward increased KYN production, thus contributing to increased systemic KYN levels.^69^ Notably, the dysregulation of the KP has also been linked to diabetes. Clinical studies have shown an increase in KP activity in patients with type 2 diabetes (T2D), which was also found to be predictive of the development of chronic kidney disease in this population.^70–72^ Interestingly, while increased KYN appears to be detrimental for the development of T2D, IDO1 has been reported to have a protective role against type 1 diabetes. In this regard, loss of IDO1 expression in human pancreatic beta-cells was identified as an early event in the development of islet dysfunction.^73^ Similarly, decreased IDO1 activity is observed in antigen-presenting cells (APCs) derived from mouse models and pediatric type 1 diabetes patients.^2,74,75^ Critically, augmenting IDO1 expression in APCs restores tolerance toward pancreatic autoantigens in mouse models of autoimmune diabetes.^74,76^ Taken together, these observations suggest the possibility that the upregulation of the KYN pathway could reflect an early adaptive response aimed at attenuating inflammation, which in chronic conditions could contribute to the development of maladaptive responses. This is further compounded in the context of renal impairment, as KYN is primarily excreted by the kidneys.^77–79^

## The Crossroads of KYN Metabolism

Once KYN is produced, its metabolic fate is determined by the local cellular context and physiological demand. This implies that the KP does not produce a single specific outcome; instead, it supports multiple functions, some of which are net positive, including neuroprotection, neurotransmission, reduced inflammation, immune regulation, NAD^+^ biosynthesis, and others that can be deleterious, such as neurotoxicity or immune evasion in the context of cancer.^2,23,26,80,81^ The following sections cover the downstream routes of KYN metabolism, leading to the synthesis of kynurenic acid (KynA), anthranilic acid (AA), kynurenine-carboxyketoalkene (Kyn-CKA), and 3-hydroxykynurenine (3-HK), focusing on their formation mechanisms, signaling actions, and contributions to tissue homeostasis and disease.

### Kynurenic Acid

KynA is one of the best-characterized downstream metabolites of KYN, and it is known for its roles in neuroprotection and immune regulation.^82,83^ KynA is formed by the irreversible transamination of KYN by kynurenine aminotransferases I–IV (KATI-IV) and 2-oxoacid cosubstrates, such as the tricarboxylic acid cycle intermediate α-ketoglutaric acid.^84–86^ KynA is highly enriched in the human cerebral cortex, putamen, and cerebrospinal fluid, with KATII responsible for KynA synthesis in astrocytes. In mice, KATII activity is reported in these same brain regions, in addition to the mouse brainstem, olfactory bulb, and retinal ganglion cells.^87,88^ In contrast, microglia and neurons exhibit a low capacity for KynA production and primarily follow the 3-HK route of KYN metabolism.^89,90^

Outside of the CNS, KynA production is mediated by other KAT isoforms.^91^ KynA production has been detected in endothelial^92^ and epithelial cells,^93,94^ pancreatic islet cells,^95^ peripheral blood mononuclear cells,^96^ skeletal muscle,^97^ erythrocytes,^98^ and hepatic cells.^82^ In addition, KynA is present in physiological fluids such as urine, serum, amniotic fluid,^99^ breast milk,^100^ synovial fluid,^101^ saliva,^102^ gastric juice, bile, and pancreatic juice.^103^ Since KynA is produced locally within tissues, its effect on the surrounding microenvironment is determined by regional synthesis, KYN availability, and the balance between KynA and 3-HK metabolism.^82,104^

#### Kynurenic Acid Modulates Glutamatergic and Cholinergic Function

KynA has been shown to act as an antagonist for glutamate receptors sensitive to *N*-methyl-d-aspartate (NMDA), both at the NMDA (IC_50_ ∼ 200 μM) and glycine (IC_50_ ∼ 10 μM) binding sites, as well as for both α-amino-3-hydroxy-5-methyl-4-isoxazolepropionic acid (AMPA) and kainate ionotropic glutamate receptors (IC_50_ ∼ 250 μM).^105–107^ Thus, KynA exerts neuroprotective effects by protecting against neuronal excitotoxic injury due to excessive NMDA receptor activation by high concentrations of glutamate or quinolinic acid (QA), another product of the KP.^108,109^ In contrast, the hypoglutamatergic hypothesis proposes that decreased activation of glutamate receptors is a main contributor to the development of cognitive disorders and schizophrenia. Notably, KynA is elevated in the brains and CSF of schizophrenic patients, particularly in the prefrontal cortex.^110^ While the mechanisms behind the elevation of KynA levels and its contributions to symptom development are unclear, long-term administration of neuroleptic drugs in rats has been observed to decrease CSF levels of KynA, and attenuation of KynA levels is associated with improved cognitive function in rodents.^110,111^ In the context of AMPA receptors, one study reported biphasic effects of KynA, with low concentrations facilitating receptor signaling and higher concentrations acting as a competitive antagonist.^112^ However, while the inhibitory actions of KynA on AMPAR signaling are well-established, the potentiating effects observed at lower concentrations have not been subsequently reproduced. In addition, KynA has been proposed to inhibit the α7 nicotinic acetylcholine receptor (α7-nAChR) with an IC_50_ ∼ 7 μM and to promote the expression of the α4β2 nicotinic acetylcholine receptor upon prolonged exposure,^113^ but unequivocal evidence for these and similar subsequent observations has proven difficult to obtain.^107,114,115^

In terms of the contribution of KynA to neurodegeneration, Alzheimer’s disease (AD) patients were observed to have higher CSF concentrations of KynA and picolinic acid (PA) than healthy controls. However, higher levels of KynA were associated with slower progression of the disease, suggesting that KynA may be an adaptive response to AD development.^116^ Similarly, elevated KynA levels in the brain can protect nigrostriatal dopamine neurons against QA-induced excitotoxic cell damage, suggesting a potential protective role in Parkinson’s disease (PD).^117^ However, while KynA levels were found to be lower in the serum and CSF of PD patients, these did not correlate with disease severity.^118,119^ Finally, in Huntington’s disease, mouse models showed that while pharmacological or genetic attenuation of kynurenine 3-monooxygenase (KMO) activity increases KynA production, reduces proinflammatory cytokines, and normalizes some electrophysiological parameters, it still fails to correct manifestations of disease progression.^120,121^ These studies are in contrast with a previous report suggesting more robust protection against HD-related neurodegeneration and mortality.^122^ Interestingly, whether HD is indeed associated with an imbalance in the KP is unclear.^123,124^

#### Kynurenic Acid Is a GPR35 Ligand

In addition to its established effects on glutamatergic signaling, KynA has also been proposed to interact with the G protein-coupled receptor GPR35, thereby influencing pathways involved in cellular survival and inflammation.^125,126^ GPR35 features a classical seven-transmembrane α-helix architecture and is expressed primarily in the small intestine, colon, and spleen in both humans and mice, with low-level expression in bone marrow, lymphoid tissue, liver, and brain observed in rodents. Rodent and human GPR35 orthologs share ∼70% homology and are activated by KynA, with EC_50_ values of ∼10 μM in rats and mice and ∼40 μM in humans.^127^ Importantly, the difference in potency between human and rodent orthologs was found to be significantly greater in other studies, calling into question the relevance of KynA as an endogenous ligand for human GPR35.^128^ Similarly, species-dependent specificity is observed for GPR35 antagonists such as CID-2745687 and ML-145, which are significantly more potent in humans than in rodents; as a result, *in vitro* or preclinical studies using CID-2745687 or ML-145 to infer the involvement of rodent GPR35 in a given pathway should be confirmed using more specific molecular approaches.^129^

In terms of pathophysiological relevance, KynA preserves mitochondrial function and suppresses apoptotic signaling in rat cardiomyocytes during ischemia–reperfusion (I/R) injury, possibly through GPR35 activation.^130,131^ Notably, these findings are consistent with work by Wyant et al., demonstrating that the cardioprotective effects of KynA in the setting of ischemic injury are lost in GPR35^–/–^ mice.^132^ GPR35 plays a dual role in regulating inflammation, acting as either a pro- or an anti-inflammatory, depending on the cell type and context. In activated neutrophils, GPR35 facilitates chemotaxis, trans-endothelial migration, infiltration of inflamed tissues, and bacterial clearance, while in macrophages, GPR35 signaling promotes the production of TNF.^133–135^ However, while the proinflammatory effects of GPR35 in mouse models are well-established, KynA has often been found to be a weak inducer of these responses.^133,134^ Furthermore, studies indicate that GPR35 activation by exogenous KynA can indeed attenuate inflammation by inhibiting the NLRP3 inflammasome, suppressing LPS-dependent IL-23 production in dendritic cells, and attenuating Th17 polarization.^136,137^ In line with these results, Agudelo et al. demonstrated that exogenous KynA promotes anti-inflammatory gene expression in the adipose tissue of mice.^138^ Notably, these effects were accompanied by increased whole-body energy expenditure and the upregulation of genes involved in fatty acid oxidation and thermogenesis. Furthermore, 2 weeks of KynA administration significantly decreased subcutaneous fat accumulation, circulating triglyceride levels, and improved glucose tolerance in mice subjected to a high-fat diet, but not in GPR35^–/–^ controls.^138^ Finally, KynA was found to ameliorate insulin resistance and palmitate-induced inflammation via the activation of GPR35–AMPK signaling and Sirtuin 6-dependent pathways in both adipose tissue and skeletal muscle.^139^

#### Kynurenic Acid–AhR Signaling Interaction

Similar to KYN, KynA promotes AhR-dependent signaling. However, KynA activates human GPR35 with 100-fold greater potency than its mouse ortholog (EC_25_ ∼ 0.1 and 10 μM, respectively).^83^ In this regard, not only do mouse and human AhR orthologs exhibit different sensitivity toward ligands, but differences are also detected among isoforms expressed in individual mouse strains.^140,141^ Interestingly, KynA cotreatment with IL-1β was shown to promote IL-6 expression in tumor cell lines, similar to previous observations with the canonical AhR ligand 2,3,7,8-tetrachlorodibenzo-*p*-dioxin (TCDD).^83^ In contrast, Wang et al. showed that AhR activation by KynA in human mesenchymal stem cells in the presence of IFN-γ and TNF-α results in the upregulation of TNF-stimulated gene 6 (TSG-6), which in turn decreases neutrophil and macrophage infiltration in a mouse model of acute lung injury.^142^ Taken together with the predominantly immunosuppressive effects of AhR engagement by KYN, these results indicate that the outcome of AhR activation is likely to be context-dependent. Furthermore, the potential presence of unrecognized bioactive metabolites can contribute to observed phenotypes via both AhR-dependent and AhR-independent pathways.^2,24,54,56^

### Anthranilic Acid

Anthranilic acid (AA) is produced by the enzyme kynureninase (KYNU), which catalyzes the hydrolytic cleavage of the amide bond of KYN to generate AA and l-alanine (Ala) in a pyridoxal-5′-phosphate-dependent manner.^143^ AA can be found in the CNS,^144^ and peripherally in urine,^145^ blood,^146,147^ and gastrointestinal tissues.^148^

#### Anthranilic Acid Signaling and Emerging Relevance in Disease

Compared with other KYN metabolites, AA signaling is incompletely characterized in humans. However, evidence has shown that AA may participate in cellular communication and transcriptional regulation in bacteria and fungi. In this regard, Song et al. demonstrated that AA regulates the transcription of quorum-sensing signal synthase-encoding genes *phcB* and *soll* in *Ralstonia solanacearum*.^149^ Similarly, in the fungus *Trichoderma*, AA has been shown to promote lateral root development through auxin signaling and *RBOHF*-induced endodermal cell wall remodeling.^150^ Interestingly, AA is emerging as a potential biomarker for disease. Elevated circulating levels of AA have been reported in depression,^144,151^ schizophrenia,^152^ rheumatoid arthritis,^153^ and type 1 diabetes.^146^ While the contribution of AA to these pathologies is unclear, these results suggest that increased AA may track with systemic inflammation, immune activation, or disruption of homeostatic KYN metabolism.

### Kynurenine-Carboxyketoalkene

Despite their clear pathophysiological relevance, the mechanisms responsible for the downstream effects of KYN are not fully defined.^154,155^ In this regard, genetic or pharmacologic modulation of rate-limiting enzymes (IDO1, TDO2) provides limited insight into the individual contributions of potentially overlapping mechanisms, such as local tryptophan depletion,^156^ activity-independent IDO1 signaling,^157^ alterations in NAD^+^ availability,^158^ and bioactive effects of individual kynurenine-derived metabolites.^159^ Notably, a significant body of literature mostly focused on UV-filter biology in the eye has demonstrated that elevated concentrations of kynurenine and glycosylated kynurenine derivatives are associated with the formation of cysteine-reactive products capable of modifying crystallin proteins.^160–165^

Molecules containing electron-withdrawing (i.e., electrophilic) groups, such as α,β-unsaturated carbonyls, react covalently with cysteine and noncysteine thiols, and to a lesser extent with amines.^166–168^ Covalent addition of electrophilic molecules to critical cysteine residues in transcription factors, transcriptional regulatory proteins, and enzymes regulates protein and cellular function. Highly sensitive cysteines are present in critical constituents of the TLR/NF-κB, NLRP3 inflammasome, and Keap1/Nrf2 signaling pathways.^166–170^ Notably, targeting these pathways *in vivo* has shown beneficial effects in both preclinical models and clinical studies.^170–173^ Thus, small molecule electrophiles have drawn pharmaceutical attention, leading to the development of compounds such as bardoxolone-methyl, nitro-oleic acid, and the FDA-approved antimultiple sclerosis drug, dimethylfumarate (Tecfidera).^173^

In view of the well-established mechanisms of redox-dependent cell signaling and the potential for KP-derived metabolites to generate cysteine-reactive metabolites, we hypothesized that a significant portion of the effects ascribed to KYN synthesis upregulation could be the result of the formation of the electrophilic derivative kynurenine-carboxyketoalkene (Kyn-CKA).^24,174,175^ The facile reaction of endogenous electrophiles with cysteine thiols, combined with substantial metabolic transformation, makes *in vivo* detection of these molecules in their free (nonadducted) form highly challenging.^166,176,177^ We approached this problem by using highly specific tandem (MS/MS) and high-resolution mass spectrometry (HRMS) to detect proximal metabolites such as unreactive reduction products and excreted urinary conjugates.^178–180^ Using this approach, we identified the following Kyn-CKA-specific metabolites: a reduced nonelectrophilic carboxyketoalkane derivative in plasma (Red-Kyn-CKA), *N*-acetyl-cysteine- and cysteine-Kyn-CKA adducts in urine (NAC-Kyn-CKA, Cys-Kyn-CKA), and an intracellular glutathione conjugate in the liver (GSH-Kyn-CKA).^24^ The formation of GSH conjugates of Kyn-CKA represents the first step in the mercapturic acid pathway, a conserved mechanism for cellular electrophile detoxification.^24,181^ Through this pathway, GSH-Kyn-CKA is exported through multidrug resistance proteins and processed by extracellular peptidases, resulting in the generation of cysteine- and *N*-acetyl-cysteine-conjugated Kyn-CKA products. Using these species as specific markers of Kyn-CKA formation, we showed that Kyn-CKA is present in cells capable of incorporating NFK or KYN, and that kynurenine administration *in vivo* results in increased levels of Kyn-CKA metabolites in plasma, tissues, and urine. Furthermore, we found that Kyn-CKA metabolites are elevated in clinical samples from both humanized sickle cell disease (SCD) mice and patients.^24^

#### Kynurenine-Carboxyketoalkene Signaling Mechanisms and Pathological Relevance

As mentioned previously, Kyn-CKA is an electrophile, meaning it can covalently modify cysteine thiols in transcription factors, signaling proteins, and enzymes, thereby altering protein and cellular function.^182^ In line with this reactivity, Kyn-CKA stabilizes and induces the expression of Nrf2 target genes, including heme oxygenase 1 (HO1), the regulatory subunit of glutamate-cysteine ligase (GCLM), and NAD­(P)­H:quinone oxidoreductase 1 (NQO1), likely via the modification of cysteine-151 in the Nrf2-inhibitory protein Keap1.^56^ In addition, Kyn-CKA directly inhibits the NF-κB pathway by reducing the nuclear localization of the p65 subunit and interfering with its binding to its DNA consensus sequence. Similarly, Kyn-CKA also inhibited the nonlike receptor pyrin domain-containing 3 (NLRP3) inflammasome and lowered pro-caspase-1 levels, thereby preventing the processing and secretion of mature IL-1β. In line with these observations, Kyn-CKA administration reduced NF-κB-dependent gene expression in tissues and attenuated proinflammatory cytokine levels in the circulation of LPS-treated mice. Furthermore, Kyn-CKA administration attenuated endotoxin-induced pulmonary microvascular vaso-occlusion in a humanized mouse SCD model.^24^ In addition to the modulation of Nrf2- and NF-κB-dependent gene expression, Kyn-CKA was found to activate AhR with 10–20-fold higher potency than KYN in a cell-based reporter assay, and to induce the expression of the AhR target genes *CYP1A1* and *CYP1B1.* ^24,183^ Notably, while AhR engagement is required for many of KYN’s immunosuppressive effects, this pathway was not required for the acute anti-inflammatory effects of Kyn-CKA in macrophages. In this regard, it was observed that Nrf2 was required for the inhibition of proinflammatory signaling by low doses of Kyn-CKA, with Nrf2-independent pathways becoming involved at higher doses.^183^

While the mechanism of Nrf2 stabilization by Kyn-CKA has been established,^56^ and it is likely that the inhibition of binding of p65 to DNA might result from covalent modification of Cysteine 38,^184–186^ the mechanism of AhR activation by Kyn-CKA is incompletely understood. In this regard, a limited structure–activity study demonstrated that the electrophilic moiety of Kyn-CKA is required for maximal AhR activation (unpublished), and that removal of the amino moiety from the benzene ring completely abrogates AhR activation.^56^ These findings suggest that the amino moiety may be necessary to stabilize Kyn-CKA within the ligand-binding pocket of the AhR. However, this could also imply that the proximal AhR ligand is not linear Kyn-CKA, but instead an intramolecularly cyclized intermediate.^24,54,59^ Overall, these findings provide evidence for a new route of KYN metabolism that is operative under basal conditions and further activated in the context of inflammation. Moreover, the formation of Kyn-CKA provides a mechanistic rationale for the observation of Nrf2-dependent gene expression secondary to increased endogenous or exogenous KYN formation.^30^

### 3-Hydroxykynurenine

One of the most studied metabolites in the KP is 3-hydroxykynurenine (3-HK), which commits KYN metabolism toward the oxidative branch of KP, and leads to 3-hydroxyanthranilic acid (3-HAA), xanthurenic acid (XA), quinolinic acid (QA), picolinic acid (PA), and NAD^+^ biosynthesis.^2^ The formation of 3-HK is catalyzed by KMO in an NADPH- and O_2_-dependent manner. The reduction of a FAD moiety by NADPH in the presence of molecular oxygen enables a 4a-peroxyflavin intermediate, which is subsequently dehydrated, leading to the release of 3-HK.^187,188^ KMO is expressed in tissues that undergo oxidative metabolism, such as the liver, immune cells (macrophages, B cells, monocytes, Kupffer cells), proximal tubule cells in the kidney, placental syncytiotrophoblasts, and microglia.^17,189^ In the CNS, the balance between the 3-HK arm of KYN metabolism, followed by microglia, and the KynA pathway favored by astrocytes can influence system homeostasis and dictate neuroprotection or neurotoxicity, as well as impact inflammation, infection, or metabolic stress.^190^

#### 3-Hydroxykynurenine: A Double-Edged Redox-Active Molecule

The *ο*-aminophenol structure in 3-HK can bind copper­(II) or iron­(III) ions, thus enabling the formation of a semiquinone-like species via a one-electron oxidation reaction. Furthermore, the subsequent reaction of the reduced metal ion with oxygen results in the formation of superoxide (O_2_^•–^), which in turn can dismutate to hydrogen peroxide (H_2_O_2_) or react with other paramagnetic substances.^191,192^ In addition, the two-electron oxidation of 3-HK results in the formation of an electrophilic quinone imine, which has the potential to covalently modify and cross-link proteins.^191,193,194^ Finally, 3-HK deamination has been shown to contribute to crystallin protein modification in the eye and the formation of several cyclic compounds, such as xanthommatin and 4,6-dihydroxyquinolinequinonecarboxylic acid (DHQCA).^195,196^ In line with its pro-oxidant activities, 3-HK induced cell death in primary cultured striatal neurons and in a neuronal hybrid cell line by generating reactive oxygen species (ROS).^197–199^ Additionally, 3-HK was shown to decrease mitochondrial membrane potential both in cultured rat cortical astrocytes and *in vivo.* ^200^ Interestingly, 3-HK has also been shown to exert protective antioxidant effects through scavenging of oxygen-derived radicals.^201–204^ Therefore, it is likely that the balance between the potentially protective and cytotoxic reactivities of 3-HK will be determined by its local concentration, the presence of chelatable metals, the oxygen tension, and the availability of cellular antioxidants.

#### 3-Hydroxykynurenine in Disease

Within the CNS, elevated 3-HK levels have been reported in clinical Huntington’s disease,^205^ AD,^206,207^ and Parkinson’s disease populations,^118,208^ as well as in animal models.^209–211^ Despite these associations, the specific contribution of 3-HK to neurodegeneration is unclear, and while pathological production of ROS is possible, the alterations in 3-HK levels could also reflect increased production of neurotoxic metabolites such as 3-hydroxyanthranilic and quinolinic acid.^212^ In addition to neurological conditions, a prospective clinical study of acute pancreatitis patients revealed that plasma 3-HK levels correlate with APACHE II scores of disease severity and markers of systemic inflammation.^213,214^ Consistent with the potential contribution of 3-HK overproduction to acute pancreatitis and multiorgan dysfunction syndrome, genetic or pharmacological KMO inhibition protected rodents against taurocholate-induced organ injury.^215^ However, in addition to virtually eliminating 3-HK from circulation, KMO disruption also led to very significant increases in serum kynurenine and kynurenic acid levels. As a result, it is possible that the protective effects of KMO inhibition could reflect the rerouting of the kynurenine pathway toward potentially cytoprotective metabolites.^24,81,82^

### Xanthurenic Acid

Xanthurenic acid (XA) is a downstream product of 3-HK metabolism that occurs through the transamination of 3-HK by KATI-IV and a 2-oxoacid cosubstrate. XA formation is highly concentrated in the brain, where it is proposed to function as a neuromodulator of glutamatergic transmission and contribute to neuroprotective signaling.^216,217^ In glutamatergic neurons, XA uptake has been shown to inhibit vesicular glutamate transport, suggesting a reduction in excitatory neurotransmission and a mitigating effect on excitotoxic injury.^218,219^ In addition, XA has been identified as an activator for the group II metabotropic glutamate receptors, mGlu2 and mGlu3.^220,221^ Finally, a recent study by Taleb et al. showed that administration of XA stimulates dopamine release in several brain regions, suggesting that XA plays a broad role in brain neuromodulation.^222^

## Route toward Nicotinamide/NAD^+^ Synthesis

The oxidative branch of the KP diverts metabolic flux toward the biosynthesis of nicotinamide adenine dinucleotide (NAD^+^). After KYN is committed to the 3-HK pathway, downstream metabolites lead to the formation of 3-HAA, the unstable intermediate α-amino-β-carboxymuconate-ε-semialdehyde (ACMS), which is further metabolized to either QA or PA, and, lastly, to NAD^+^. The following sections summarize the formation, signaling interactions, and pathologies associated with these intermediates.

### 3-Hydroxyanthranilic Acid

3-Hydroxyanthranilic acid (3-HAA) is predominantly formed from 3-HK via KYNU and is detected in the liver, kidney, macrophages, and the CNS.^223–226^ In the brain, AA hydroxylation by an unidentified enzyme has been described to result in 3-hydroxyanthranilic acid formation.^227,228^ Functionally, 3-HAA contains the same *ο*-aminophenol motif present in 3-HK and has been reported to act as a bioactive redox- and immune-modulating metabolite. At low physiologic concentrations, 3-HAA demonstrates antioxidant activity similar to 3-HK, as it can scavenge free radicals, inhibit plasma lipid peroxidation, and interact with iron molecules.^229–231^ In cultured human astrocytes, 3-HAA was identified as an antioxidant that suppressed glial cytokine and chemokine expression and reduced cytokine-induced death. It was also revealed that it can induce HO1 gene expression, an enzyme that induces anti-inflammatory signaling.^224^ Consistent with these findings, a recent study by Dang et al. showed that 3-HAA activates the Nrf2/SKN-1 oxidative stress response pathway and extends lifespan in both *Caenorhabditis elegans* and mice.^232^ In this regard, although the mechanism responsible for activating Nrf2-dependent genes remains unknown, two potential scenarios can be proposed. The first implies the direct modification of Keap1 by an electrophilic amino quinone resulting from the two-electron oxidation of 3-HAA, while the other mechanism entails the oxidation of Keap1 cysteine residues by ROS generated by metal-catalyzed 3-HAA autoxidation, both culminating in Nrf2 stabilization and nuclear translocation.^174,233^ Furthermore, 3-HAA is reported to protect the lungs against hyperoxia-induced injury by inhibiting ferroptosis, binding to ferritin heavy chain 1 (FTH1), and disrupting the interaction between nuclear receptor coactivator 4 and FTH1.^234^ In immune cells, 3-HAA suppresses T-cell proliferation by inhibiting dendritic cell-mediated activation, inducing T-cell apoptosis, and decreasing inflammatory cytokine production by interfering with NF-κB signaling.^235,236^ Interestingly, similar anti-inflammatory responses have been observed with cysteine-reactive electrophilic species.^237–239^ Finally, and in parallel to observations made with 3-HK, exposure to elevated levels of 3-HAA inhibits mitochondrial respiration, decreases mitochondrial membrane potential, and leads to downstream impairment of cellular function.^200,240^ These findings suggest that 3-HAA can promote cytoprotection at lower concentrations but can also impair cellular function in a context-dependent manner.

### Cinnabarinic Acid

Cinnabarinic acid (CA) is formed by 3-HAA dimerization following two consecutive two-electron oxidation reactions involving either low molecular weight oxidants or compound I-generating enzymes such as peroxidases and catalase.^230,241^ CA has been detected in multiple tissues and physiological fluids, including the brain, lungs, liver, spleen, spinal cord, immune cells, blood, and urine. However, its concentration under normal physiological conditions is lower than that of other KP metabolites.^242–244^ Fazio et al. identified CA as a partial agonist of mGlu4 receptors, reducing neurotransmitter release and, through this interaction, conferring neuroprotection. Notably, these authors also note that when mGlu4 is knocked out, CA can partially protect neurons against NMDA toxicity and inhibit cAMP accumulation at higher doses (100 μM), suggesting the existence of additional uncharacterized mechanisms of CA-dependent neuromodulation.^242^ Interestingly, CA has also been reported to inhibit IDO, suggesting that it may play a role in feedback regulation of the KP.^245,246^ As is the case with other intermediates in the kynurenine pathway and tryptophan metabolism in general, CA has been reported to be an endogenous AhR ligand. However, while CA induced IL-22 production in CD4^+^ T cells in an AhR-dependent manner, it was significantly less potent at inducing the AhR-regulated gene *Cyp1a1* than both KYN and KynA. Interestingly, neither KYN nor KynA elicited IL-22 production from CD4^+^ T cells.^244^ Similarly, while CA protected hepatocytes against apoptotic insults and lipotoxicity via increased stanniocalcin 2 (Stc2) expression—a response that was lost in AhR^–/–^ mice—canonical AhR ligands such as TCDD failed to elicit the same response.^243,247,248^ Therefore, these results suggest that additional AhR-independent mechanisms may contribute to the physiological actions of CA.^249^ Finally, CA also displays context-dependent redox effects by inhibiting mitochondrial respiration,^239^ promoting ROS formation, and inducing caspase-3-dependent apoptosis in thymocytes.^250^

### α-Amino-β-Carboxymuconate-ε-Semialdehyde (ACMS)

α-Amino-β-carboxymuconate-ε-semialdehyde (ACMS) is generated from 3-HAA through an oxidative ring cleavage reaction catalyzed by the nonheme iron-containing enzyme 3-hydroxyanthranilate 3,4-dioxygenase (3-HAO). This enzyme catalyzes the opening of the aromatic ring via cleavage of the C3–C4 bond of 3-HAA in an oxygen-dependent manner to eventually generate ACMS.^251,252^ The formation of ACMS occurs in several metabolically active tissues, including the brain, liver, and kidneys.^253^

The production of ACMS represents another branching point in the KP, leading to either the formation of picolinic acid (PA) or quinolinic acid (QA) and to NAD^+^ biosynthesis. The metabolic fate of ACMS is determined by ACMS decarboxylase (ACMSD). This enzyme catalyzes the decarboxylation of ACMS to 2-aminouconate 6-semialdehyde (AMS), which in turn can be converted nonenzymatically to PA via an intramolecular reaction or be oxidized by AMS dehydrogenase (AMSDH, aka ALDH8A1) to 2-aminomuconate (2-AM).^253–255^ Interestingly, 2-AM can be further metabolized to give rise to the formation of glutaryl-CoA and then crotonyl-CoA, which in turn has been proposed to regulate gene expression via enzyme-dependent covalent modification of histones.^256,257^ Finally, when ACMSD activity is low, ACMS undergoes a spontaneous cyclization reaction to generate QA.^258,259^

### Picolinic Acid

Picolinic acid (PA) arises when ACMS is decarboxylated by ACMSD to form AMS. AMS can then either enter the Krebs cycle or undergo nonenzymatic cyclization to form PA.^253,254,260^ The formation of PA is favored in tissues with high ACMSD activity, in particular, the liver and the kidney;^261^ however, it can also be detected in the brain,^262^ immune cells,^263^ CSF,^264^ plasma,^265^ and is excreted in urine.^266,267^

#### Picolinic Acid Signaling and Its Physiological Role

PA possesses high-affinity metal-chelating properties, as well as neuroprotective, immunomodulatory, and antiproliferative effects, although many of these findings were obtained using supraphysiological concentrations.^262,268^ In terms of metal-chelating properties, it was identified that it could chelate copper, iron, nickel, and zinc, often acting as a bidentate ligand involving one or more PA units per metal atom.^269^ The strong metal-binding activity of PA has been shown to inhibit iron uptake and suppress cell growth in human erythroleukemic cell lines.^270^ PA also demonstrates neuroprotective activity in cell culture and animal models by antagonizing QA-induced neurotoxicity through zinc chelation.^271,272^ Similarly, millimolar concentrations of PA enhance macrophage effector function by increasing INFγ-dependent nitric oxide synthase (NOS) gene expression and inducing the MIP1α and MIPβ to promote antimicrobial activity.^273–275^ Finally, PA suppresses the metabolic activity of CD4^+^ T cells, inhibits c-Myc activation, and exhibits broad antiviral activity *in vitro*, also at high concentrations.^263,276,277^ Clinically, reduced serum PA levels are associated with severe depressive states, suggesting a biomarker role in psychiatric disorders.^278^

### Quinolinic Acid and NAD^+^ Biosynthesis

Quinolinic acid (QA) is formed from ACMS through a spontaneous, nonenzymatic intramolecular cyclization reaction.^254,279^ Within the CNS, activated resident microglia and infiltrating macrophages are the predominant sources of QA, while peripherally, QA production occurs in the liver.^280–282^

#### Quinolinic Acid Signaling and Toxicity

QA is often cited as a potent agonist for the *N*-methyl-d-aspartate (NMDA) receptor, targeting receptor subunits NR2A and NR2B.^2,283^ As a result, sustained QA-NMDA receptor activation is proposed to cause neuronal excitotoxicity by inducing excessive Ca^2+^ influx into the cell, leading to cellular dysfunction and cell death.^284–286^ Additionally, QA inhibits vesicular and astrocyte glutamate uptake and increases basal glutamate release from synaptosomal preparations.^287,288^ However, whereas glutamate activates NMDA receptors with an EC_50_ of ∼2 μM, QA requires a 1000-fold higher concentration to exert comparable effects; similarly, the effects of QA on glutamate homeostasis also require millimolar concentrations. These observations call into question whether QA promotes glutamate-dependent neurological injury under physiologically relevant conditions.^289–291^

In addition to a potential role in excitotoxicity, QA can form a redox-active QA–Fe^2+^ complex, which can generate ROS to trigger lipid peroxidation and cause DNA damage.^109,292–295^ Interestingly, nanomolar to low micromolar concentrations of QA can increase the activity of the neuronal isoform of nitric oxide synthase in astrocytes and neurons, resulting in heightened poly­(ADP-ribose) polymerase (PARP) activation, NAD^+^ depletion, DNA damage, and ultimately cell death.^296^ QA can also contribute to neuroinflammation and immune activation. In this regard, intrastriatal injections of QA activate NF-κB to induce proinflammatory cytokines such as TNF-α and IL-6, promote neuronal and astrocytic apoptosis, and disrupt the integrity of the blood–brain barrier.^286,297–305^ Similarly, QA was observed to upregulate monocyte chemoattractant protein-1 (MCP-1), CCL5, IL-8, SDF-1α, CXCL9, and fractalkine (CX3CL1), as well as increase the expression of chemokine receptors CXCR4, CCR5, and CCR4 in fetal human astrocytes.^306^ Taken together, these findings suggest that direct pro-oxidant and proinflammatory effects might significantly contribute to the neurotoxic phenotype associated with QA overproduction or *in situ* administration.

#### Quinolinic Acid to NAD^+^ Biosynthesis

QA is converted to nicotinic acid mononucleotide (NAMN) by quinolinate phosphoribosyltransferase (QPRT) using 5-phosphoribosyl-1-pyrophosphate (PRPP) as a cosubstrate, which is the rate-limiting step in NAD^+^ biosynthesis. Subsequently, NAMN is adenylated by NAMN adenyltransferases (NMNATs) to generate nicotinic acid adenine dinucleotide (NAAD), which in turn is converted into NAD^+^ by glutamine-dependent NAD^+^ synthase (NADSYN).^307,308^ In this regard, the KP functions alongside the Preiss–Handler and the NAD^+^ salvage pathways to maintain NAD^+^ homeostasis.^309,310^

#### Quinolinic Acid in Pathology

In the CNS, QA accumulation has been implicated in neurodegeneration, neuroinflammation, and psychiatric disorders. Increased levels of QA have been reported in PD, AD, HD, amyotrophic lateral sclerosis, multiple sclerosis, and HIV-related cognitive decline.^311–313^ Similarly, in psychiatric disorders, QA levels are elevated in depression, schizophrenia, suicidal individuals, and autism.^286,314^ In the case of AD, QA was reported to be present in amyloid plaques and within dystrophic neurons, and to contribute to phosphorylated tau accumulation and cytoskeleton destabilization.^315,316^ Interestingly, while elevated systemic levels of QA are observed in liver disease and following injury, exogenous QA administration prevented hepatic lipid accumulation in a high-fat diet mouse model by suppressing lipoprotein lipase and fatty-acid translocase expression. These findings suggest the intriguing possibility that QA may function as an adaptive mediator in extracerebral tissues, potentially capable of protecting against hepatic injury.^282,317,318^ In cancer, exogenous administration of QA was shown to activate β-catenin and proliferation of human colon cancer cells and to enhance tumor growth.^319^ In glioma, QA accumulation has been shown to correlate with tumor stage, and treatment of glioma cells with QA has been shown to reduce glioma sensitivity to Temozolomide, in part by increasing NAD^+^ availability.^312^ In summary, excessive production or inadequate catabolism of QA can contribute to disease development through NMDA-dependent excitotoxicity, oxidative stress, and proinflammatory signaling.

## The Serotonin Pathway

The synthesis of serotonin (5-hydroxytryptamine) accounts for an estimated 2% of Trp metabolism, and while this pathway consumes a relatively minor fraction of this amino acid, it crucially links Trp catabolism to neurotransmission, circadian regulation, and neuroendocrine signaling (Figure 2).^4,10,11^ The serotonin pathway is regulated via the rate-limiting hydroxylation of Trp to 5-hydroxytryptophan (5-HTP) by tryptophan hydroxylases (TPH1 and TPH2). TPH1 is highly expressed in the pineal gland and peripheral tissues, including enterochromaffin cells (EC) of the gastrointestinal tract, while TPH2 is elevated in serotonergic neurons in the pons and brainstem raphe nuclei.^320–322^

**Figure 2:**
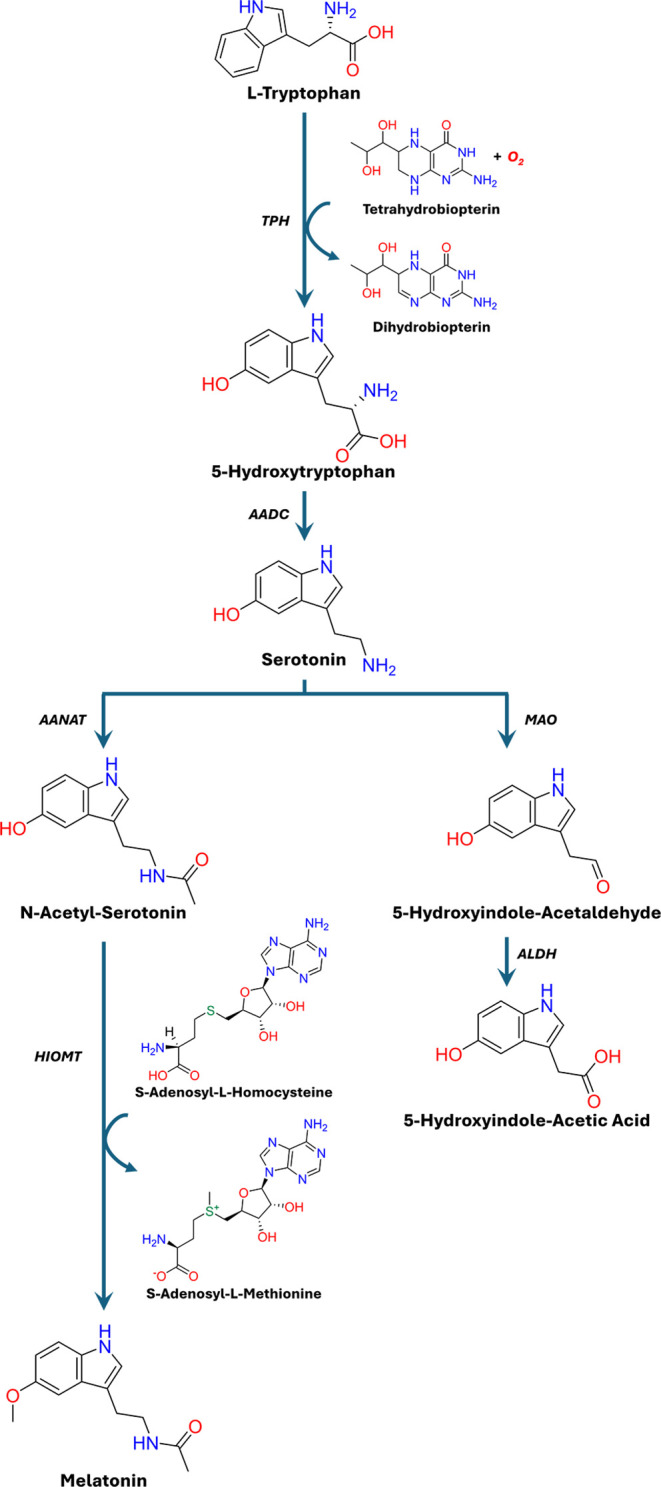
Overview of the serotonin pathway of l-tryptophan catabolism. l-Tryptophan is hydroxylated by tryptophan hydroxylase 1 or 2 (TPH1/TPH2) to produce 5-hydroxytryptophan (5-HTP), which is the rate-limiting step in serotonin synthesis. 5-HTP is then decarboxylated by aromatic l-amino decarboxylase (AADC) to form 5-hydroxytryptamine (serotonin, 5-HT). Serotonin can be further metabolized into various metabolites. In the melatonin pathway, serotonin is acetylated by arylalkylamine *N*-acetyltransferase (AANAT), the rate-limiting step in melatonin production, to produce *N*-acetylserotonin (NAS). NAS can then be methylated by hydroxyindole-O-methyltransferase (HIOMT) to form melatonin. In the 5-hydroxyindole–acetic acid pathway, serotonin undergoes oxidative deamination by monoamine oxidase-A (MAO-A) to generate 5-hydroxyindole-acetaldehyde, which is then metabolized by aldehyde dehydrogenase (ALDH) to 5-hydroxyindole–acetic acid, the primary circulating and urinary metabolite.

### Serotonin

The EC cells in the gastrointestinal tract are responsible for producing 95% of the total serotonin pool. The first step in serotonin synthesis is catalyzed by tryptophan hydroxylases, a family of aromatic amino acid hydroxylases that utilize tetrahydrobiopterin and molecular oxygen as cosubstrates and are activated by CaMKII- and PKA-dependent serine phosphorylation. Following TPH-mediated hydroxylation, 5-hydroxytryptophan is decarboxylated by aromatic l-amino acid decarboxylase (AADC) to form serotonin, which is then secreted into the bloodstream and incorporated by circulating platelets for release upon activation.^4,323–326^

Serotonin exerts well-established roles in neurotransmission, metabolic regulation, and immune signaling across the central and peripheral nervous systems, with most of these being dependent on the engagement of seven families of 5-HT receptors.^327–329^ 5-HT receptors are typically G-protein-coupled receptors (Gi/o, Gq/11, or Gs), which upon activation engage with intracellular effectors such as adenylate cyclase/PKA, Ca^2+^/calmodulin PKC, and phospholipase-C/diacylglycerol/IP3 to mediate downstream signaling.^330–333^ A notable exception is 5-HT3, which operates as a ligand-gated ion channel.^334^ In addition to receptor-dependent signaling, serotonin also signals through the covalent modification of glutamine residues via a reaction termed serotonylation. Serotonylation is an irreversible process catalyzed by transglutaminases, which occurs intracellularly following either serotonin uptake by SERT proteins or upon endogenous synthesis.^335–337^ Finally, Manzella et al. demonstrated that intracellular accumulation of serotonin in intestinal epithelial cells by SERT induces CYP1A1 expression through AhR, and that SERT deficiency *in vivo* impairs activation of AhR in the intestines.^338^

In the pancreas, serotonin is synthesized and secreted by β-cells, where it functions as an autocrine and paracrine regulator of insulin secretion. Serotonin signals through 5-HT3 and Gαq-coupled 5-HT2b receptors, as well as via receptor-independent serotonylation, to inhibit glucagon secretion from α-cells.^339–342^ Additionally, pancreatic β-cells express the serotonergic transcription factor Pet1/Fev, which helps to regulate β-cell function, the expression of insulin-related genes, and the glucose transporter Slc2a2 (GLUT2).^343,344^ In this regard, serotonin dysregulation has been associated with the development of obesity and diabetes.^345,346^

Within the GI, serotonin functions as a paracrine signal to regulate intestinal motility, epithelial secretion, peristaltic reflex, vasodilation, and sensory signaling via receptors in enteric neurons, smooth muscle, and epithelial cells.^347,348^ Serotonin has also been observed to influence bile acid homeostasis by enhancing the expression of the apical sodium-dependent bile salt transporter (ASBT), which in turn modulates bile acid uptake.^349^ In response to inflammation, serotonin contributes to immune regulation by activating NADPH oxidase 2 (Nox2) and promoting the recruitment of innate immune cells such as dendritic cells, monocytes, mast cells, and eosinophils.^350^ In addition, serotonin induces IL-8, IL-1β, and IL-6 secretion by activating the NF-κB pathway.^351^ Notably, and in contrast to the previous results in mouse models, serotonin was found to reduce TNF-induced inflammation and leukocyte recruitment in human cell-based intestinal organoids, thus suggesting critical differences between the immunomodulatory actions of serotonin in humans and mice.^352^ In this regard, dysregulated serotonin tone has been identified in the setting of gastrointestinal conditions such as IBS.^353,354^

In the cardiovascular system, serotonin functions as both a vasoactive mediator and a regulator of autonomic cardiac function. Mechanistically, serotonergic signaling from the raphe nuclei modulates both sympathetic and parasympathetic tone. Activation of the 5-HT1A receptor has been described to result in sympatho-inhibition and vagal bradycardia, whereas stimulation of 5-HT2A/B receptors leads to sympatho-excitation, elevated arterial blood pressure, and tachycardia.^355–358^ Serotonin contributes to heart valve dysfunction by upregulating ERK1/2 and MAPK signaling and TGFβ via the action of 5-HT2A receptors.^359^ 5-HT2A receptors drive serotonin-induced pulmonary artery adventitial fibroblast activation and phenotypic change by regulating the TGFβ-1/Smad3 signaling pathway.^360^ In addition, serotonin regulates vascular tone via interactions with 5-HT1B, 5-HT2A/b, 5-HT4, and 5-HT7 receptors expressed on smooth muscle and endothelial cells. 5-HT2A/B has been shown to mediate contraction or relaxation of the aorta and the mesenteric arteries, and 5-HT4 and 7 are found in the coronary arteries.^361,362^ Additionally, serotonin has been shown to promote blood vessel growth by increasing the proliferation of vascular endothelial and smooth muscle cells via 5-HT2A receptors.^363,364^ In the heart, serotonin exerts chronotropic and ionotropic effects through 5-HT4 receptors on cardiomyocytes, where it enhances pacemaker currents in atrial myocytes and increases intracellular calcium, which, as a result increases heart rate and contractile activity.^358,365–367^ Dysregulation of serotonin in the heart has been shown to be linked to chronic heart failure, coronary artery disease, and a defective mitral valve.^368–370^

Within the CNS, serotonin functions as a neurotransmitter and neuromodulator implicated in cognition, mood, impulse control, and motor function.^4,371–373^ In this location, serotonin is released by serotonergic neurons in the brainstem raphe nuclei and distributed throughout the brain.^374^ The 5-HT1A receptor signals through G protein-coupled receptors by triggering cAMP synthesis, which then activates PKA, directly or indirectly triggering phosphorylation of various transcription factors such as CREB and BDNF.^375^ Anxiety, depression, and the pathophysiology of cognitive function impairment have all been linked to the cAMP–PKA–CREB–BDNF signaling pathway, which is a crucial integrator of several neuromodulator activities. In SERT knockout mice, it was observed that there was a decrease in protein levels of CREB and BDNF and an increase in levels of anxiety and cognitive impairment.^376^ Serotonin also modulates the release of glutamate, GABA, dopamine, and norepinephrine, making it a central regulatory molecule that can influence a wide range of neural signaling networks.^11,377–379^ A discussion on how serotonin modulates the neural circuitry and emotion is beyond the scope of this review and is extensively covered in these references.^380–383^

### *N*-Acetylserotonin and Melatonin

*N*-Acetylserotonin (NAS) is primarily synthesized in the pineal gland through the acetylation of serotonin by arylalkylamine *N*-acetyltransferase (AANAT), which represents the rate-limiting step in melatonin production.^384,385^ Prior to its conversion to melatonin, NAS acts as an agonist of the tropomyosin receptor kinase B (TrkB), and through this interaction, it exhibits circadian antidepressant effects.^386^ Through TrkB, NAS promotes neuronal plasticity, supports neurogenesis, and enhances neuronal survival.^387^ Furthermore, NAS has been shown to confer neuroprotection by inhibiting mitochondrial death pathways and autophagic activation in ischemia models.^388^

The last step in melatonin synthesis is catalyzed by hydroxyindole-O-methyltransferase (HIOMT), which transfers a methyl group from *S*-adenosylmethionine to NAS.^389^ Melatonin is secreted by the pineal gland and retina and exerts pleiotropic effects through both receptor-activated and independent mechanisms. Melatonin signals through high-affinity GPR-associated melatonin type 1 and 2 receptors (MT1, MT2), to regulate glucose metabolism,^390^ inhibit stress-mediated cytochrome c release and caspase activation,^391^ reduce cAMP and inhibit insulin release in mouse islets and clonal insulin-secreting cells,^392^ and regulate circadian rhythm.^393–395^ In rabbits and hamsters, melatonin also binds to NAD­(P)H dehydrogenase:quinone 2 (NQO2, previously known as MT3 receptor), albeit with significantly lower affinity than to MT1 and MT2.^396,397^ Collectively, melatonin is implicated in blood pressure and autonomic cardiovascular regulation, immune tone, and potentially in the detoxification of free radicals.^396,398–405^ A detailed discussion of melatonin’s signaling actions can be found in this reference.^406^

Together, the NAS–melatonin axis links Trp catabolism to the circadian rhythm, tropic signaling, mitochondrial function, and redox control. In this regard, the dysregulation of NAS and melatonin has been linked to neurodegeneration,^407^ metabolic disease,^408^ and neuropsychiatric disorders,^409^ further highlighting the importance of the serotonin pathway in maintaining homeostasis.

### 5-Hydroxyindoleacetic Acid

5-Hydroxyindoleacetic acid (5-HIAA) is generated in a two-step process through the oxidative deamination of serotonin by monoamine oxidase A (MAO-A), forming 5-hydroxyindole acetaldehyde, which is then oxidized by aldehyde dehydrogenase (ALDH) to generate 5-HIAA, primarily in the liver and kidneys.^410^ Interestingly, 5-HIAA has recently been shown to act as a nanomolar-affinity ligand for GPR35, capable of stimulating neutrophil recruitment and trans-endothelial migration at sites of platelet and mast cell activation.^411^ Of note, 5-HIAA exhibited significantly higher potency for GPR35 activation than other putative endogenous ligands, such as kynurenic acid and 2-acyl lysophosphatidic acid.^411^ Clinically, 5-HIAA has been proposed as a biomarker for neuroendocrine tumors^410,412^ and septic shock.^413^

## The Microbial Indole Pathway of Tryptophan Metabolism

Approximately 4–6% of dietary Trp is metabolized by a variety of intestinal gut microbiota through the indole pathway (IP).^414^ Within the gut, microbial Trp metabolism can occur through four different mechanisms: the indole pathway, the indole-3–pyruvate pathway, the tryptamine pathway, and the indole-3–acetamide pathway (Figure 3). Together, their products have central roles in maintaining the mucosal barrier integrity, immune regulation, neuromodulation, and host–microbe communication, and can either complement or counterbalance KP metabolites.^5–9^

**Figure 3:**
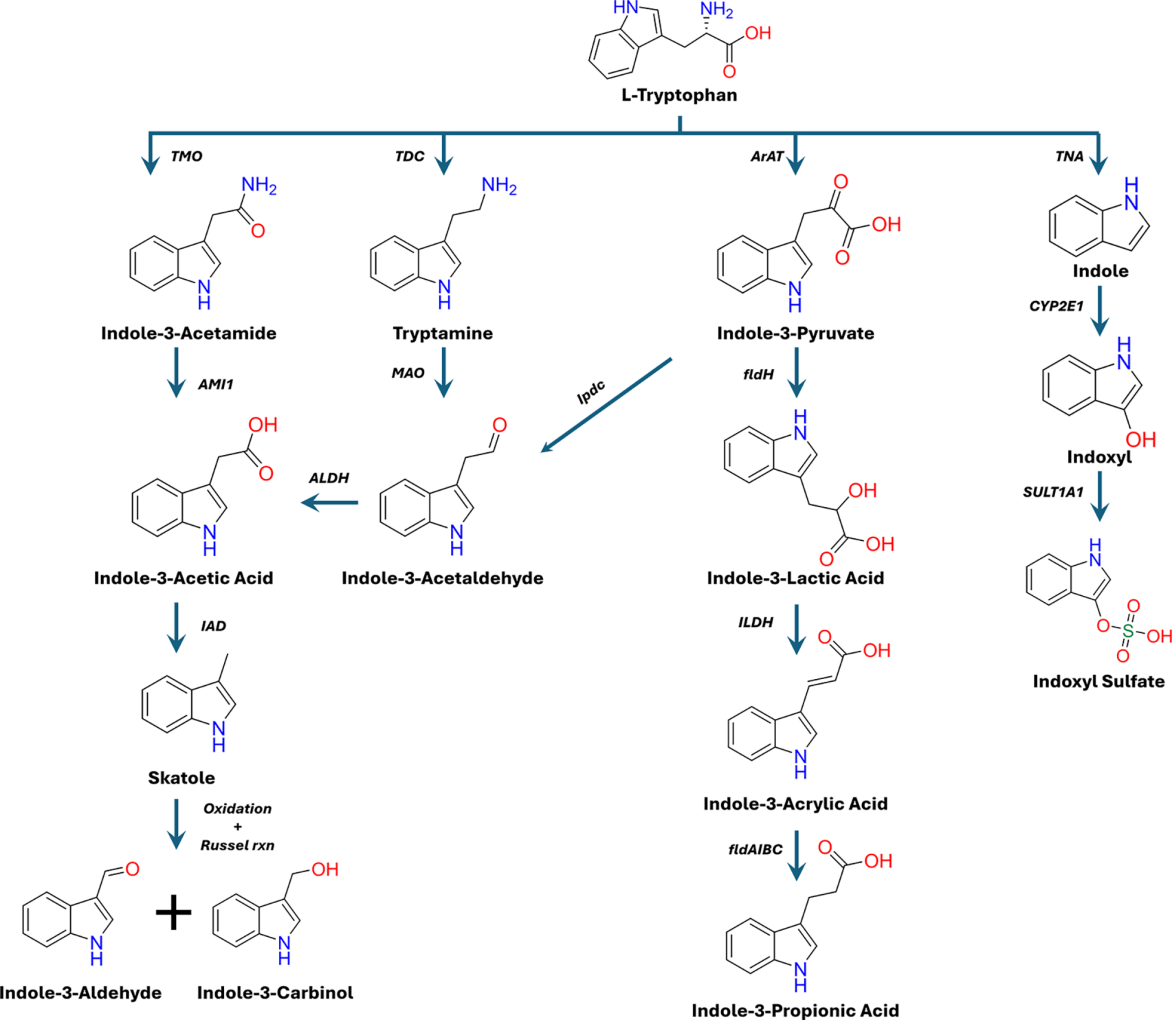
Overview of the gut microbial indole pathway (IP) of l-tryptophan catabolism. l-Tryptophan is metabolized by the intestinal microbiota into diverse indole derivatives via the Indole (IP), Indole-3–pyruvate (I3P), Tryptamine (Try), and Indole-3–acetamide (I3AM) pathways. In the IP, tryptophanase (TNA) converts l-tryptophan to indole, which can then be oxidized in the liver by cytochrome P450 2E1 (CYP2E1) to form indoxyl, which is subsequently converted to Indoxyl Sulfate (IS) by sulfotransferase-1A1 (SULT1A1). In the I3P pathway, aromatic amino acid aminotransferase (ArAT) catalyzes the transamination of l-tryptophan to I3P. I3P can then be decarboxylated by microbial indole-3-pyruvate decarboxylase (Ipdc) and shuttled into the Try pathway or reduced by microbial phenyllactate dehydrogenase (fldH) to Indole-3-Lactic Acid (I3L). I3L is then reduced to indole-3-acrylic acid (I3A) by indolelactic acid dehydrogenase (ILDH). I3A can then be further reduced to indole-3-propionic acid (I3PA) by the microbial phenyllactate dehydrogenase gene cluster AIBC (fldAIBC). In the Try pathway, the Try-derived indole is formed through the decarboxylation of l-tryptophan by tryptophan decarboxylase (*TDC*). Try is then metabolized by monoamine oxidases A and B (MAOs) to generate indole-3-acetaldehyde (I3AAld). I3AALD can also feed into the I3AM pathway via its conversion to Indole-3-Acetic Acid (I3AA) by indole-3-acetaldehyde dehydrogenase (ALDH). In the I3AM pathway, l-tryptophan is oxidized by tryptophan-2-monooxygenase (TMO) to form I3AM, which in turn is hydrolyzed by I3AM hydrolase (AMI1) to form I3AA. I3AA undergoes decarboxylation by I3AA decarboxylase (IAD) to form skatole, which can further decompose into indole-3-aldehyde (I3Ald) and Indole-3-Carbinol (I3C).

### Indole Pathway

Trp catabolism via the indole pathway is initiated by the bacterial enzyme tryptophanase (TnaA), which catalyzes the conversion of Trp to indole, pyruvate, and ammonia.^415^ This enzymatic activity is conserved among commensal and pathogenic bacteria, including *Escherichia coli*, *Clostridium*, *Bacteroides*, *Proteus*, and *Lactobacillus.* ^416^ In the gut, indole has multiple roles in microbial physiology, including bacterial quorum sensing, biofilm formation, motility, virulence gene expression, plasmid stability, and antibiotic resistance.^6,7^ Furthermore, indole also serves as a potent signaling molecule in the intestinal lumen by enhancing tight junction integrity, increasing mucin production, promoting epithelial differentiation to strengthen intestinal barriers, inhibiting microbial translocation, and reducing inflammation.^417–419^ Of note, the intracellular concentration of indole can reach up to 60 mM in *E. coli*, with human intestines reported to contain over 1 mM of this metabolite.^419^ From a vertebrate signaling perspective, indole activates human AhR but exhibits marginal potency for the mouse ortholog.^420^ Nevertheless, its elevated concentration in the intestine suggests that indole could still be a physiologically relevant AhR agonist in this compartment.^421^ Finally, and in addition to its potential role as an AhR ligand, indole has been shown to activate the pregnane X receptor (PXR) in intestinal cells with an EC_50_ ∼ 300 μM and to contribute to the regulation of intestinal barrier function.^422,423^ In this regard, exogenous administration of indole protects germ-free mice against experimental colitis.^424^

Indole can be further metabolized by host–microbes to generate indoxyl and its downstream conjugate, indoxyl sulfate (IS). In the liver, indoles are oxidized to indoxyl by cytochrome P450-dependent enzymes such as CYP2E1.^425,426^ While indoxyl’s signaling activity remains poorly understood, its downstream conjugate, indoxyl sulfate (IS), generated by sulfotransferase-1A1 (SULT1A1), is a biologically active metabolite.^427^ IS is a well-documented uremic toxin that accumulates in the body due to its inability to be excreted through urine and has been proposed to contribute to chronic kidney disease (CKD) and renal failure development.^428^ In this regard, IS has been shown to function as an endogenous AhR agonist, with EC_50_ values of ∼10 nM and 6 μM in reporter assays for the human and mouse orthologs, respectively.^429^ Through AhR signaling, IS synergizes with IL-1β and TNF to induce IL-6 and E-selectin expression in endothelial cells, thereby enhancing leukocyte–endothelial cell interactions and promoting leukocyte recruitment to the vascular wall.^429,430^ In addition, IS was found to promote proinflammatory conditions by activating human macrophages via Dll4-Notch signaling at concentrations consistent with those observed in severe CKD patients.^431^ Finally, IS has also been observed to affect vascular and cardiac function. In vascular smooth muscle cells, IS activates the epidermal growth factor receptor (EGFR) to enhance angiotensin-II signaling, leading to vascular dysfunction.^432^ Furthermore, in CKD rats, IS was shown to alter cardiac electrical signaling by reducing fast transient outward potassium channel currents via ROS production and MAPK/NF-κB signaling, as well as prolonging the QT interval, thereby increasing susceptibility to arrhythmia.^433^

### Indole-3–Pyruvate Pathway

Aromatic amino acid aminotransferase (ArAT) catalyzes the transamination of Trp to indole-3-pyruvate (I3P) in the gut.^3,434^ Through this pathway, several biologically active indole derivatives are formed, including indole-3-lactate (I3L), indole-3-acrylic acid (I3A), and indole-3-propionic acid (I3PA).^3^ I3P was initially identified as having the ability to activate AhR-dependent signaling, with subsequent work demonstrating that its spontaneous decomposition to di-indole species in aqueous solution is necessary to significantly engage this pathway.^435–437^ In this context, I3P administration protected mice against experimental colitis and was observed to increase the expression of IL-10-producing T cells in the intestinal lamina propria and promoted the generation of anti-inflammatory dendritic cells.^438^ Interestingly, I3P was shown to inhibit LPS-induced pro-IL-1β production by macrophages in an AhR-independent manner by inhibiting HIF1α stabilization, albeit at submillimolar and millimolar concentrations.^439^ I3P is enzymatically reduced by phenyllactate dehydrogenases (fldH) to give rise to the formation of I3L, which has been proposed to engage AhR to attenuate IL-8 expression, enhance neurite outgrowth, decrease neuroinflammation, and reduce amyloidopathy by promoting amyloid-β plaque clearance in microglia and astrocytes.^440–443^ Interestingly, a recent characterization of the relative potency and efficacy of different indole derivatives as human AhR agonists failed to reveal any significant activity for I3L.^421^

The reduction of I3L by microbial indolelactic acid dehydrogenase (ILDH) results in the formation of the α,β-unsaturated carbonyl-containing product I3A. This metabolite was discovered as a product of tryptophan metabolism by *Peptostreptococcus russellii*, a bacterium known to attenuate experimental colitis in mice.^444^ In addition to being a potent AhR ligand for both mouse and human isoforms, I3A increased the expression of IL-10 and attenuated the production of proinflammatory cytokines in activated macrophages, as well as promoted mucin production in goblet cells. Furthermore, I3A was found to potently upregulate the expression of Nrf2-controlled antioxidant genes.^421,444^ In this regard, while there is considerable overlap between AhR- and Nrf2-regulated genes, the presence of an electrophilic moiety in I3A strongly argues for a direct role in Nrf2 stabilization secondary to covalent Keap1 modification.^168,445,446^ Notably, I3A was equally potent at inhibiting proinflammatory signaling in mouse and human macrophages.

The terminal product of the I3P pathway is I3PA, generated by the reduction of I3A by the phenyllactate dehydratase *fldC.* ^447^ I3PA has been proposed to act as a hydroxyl radical scavenger, although the physiological relevance of these findings is unclear, given the essentially indiscriminate, diffusion-controlled reactivity of hydroxyl radicals toward most biological molecules.^448,449^ Similar to other indoles, I3PA is a weak agonist for human AhR but has been proposed to act as a species-specific activator of the pregnane X receptor (PXR).^421,423,450^ More specifically, while I3PA potently activated mouse PXR on its own, activation of the human isoform required coincubation with physiologically relevant millimolar concentrations of indole.^419,423^ Importantly, I3PA administration attenuated intestinal inflammation and maintained barrier integrity in an epithelial PXR-dependent manner.^423^ Furthermore, dietary I3PA inhibited gut dysbiosis, prevented intestinal barrier damage, and attenuated steatohepatitis in rats fed a high-fat diet.^451^ In patients, decreased circulating I3PA levels were shown to be associated with atherosclerotic cardiovascular disease, and I3PA supplementation alleviated plaque development in ApoE^–/–^ mice by promoting cholesterol efflux from macrophages to ApoA-I, suggesting important beneficial effects beyond the gut.^452,453^ Finally, I3PA was also found to have a direct anti-inflammatory effect in macrophages and to inhibit CD4^+^ T cell differentiation into proinflammatory Th1 and Th17 phenotypes.^450,451^ In this regard, while the mechanism responsible for the anti-inflammatory effects on macrophages remains to be elucidated, the effects of I3PA on CD4^+^ T cells were ascribed to a PPAR-β/δ-dependent increase in mitochondrial respiration secondary to higher rates of fatty acid and amino acid oxidation.^450,451^

Interestingly, while indole metabolism is usually associated with the microbiome, the gene product for interleukin 4-induced gene 1 (IL-4I1) has been shown to possess an l-amino acid oxidase activity capable of metabolizing tryptophan into I3P.^454^ Moreover, in human tumors, IL-4I1 was found to associate more frequently with AhR activity than either IDO1 or TDO2, suggesting a potential role in Trp-dependent immunomodulation in the tumor microenvironment.^455^ Notably, IL-4I1 is highly expressed in mature dendritic cells, but unlike IDO1, it is also actively secreted into the extracellular milieu. Moreover, as intracellular IL-4I1 is predominantly associated with vesicles and thus potentially physically separated from its substrates, it has been hypothesized that its primary actions are extracellular, where it can modulate T cell activation by promoting the synthesis of bioactive indoles.^454,456^ Of note, IL-4I1 expression leads to significant increases in several indole metabolites, including indole-3-acetic acid (IAA), indole-3-aldehyde (I3A), and indole-3-lactic acid (I3L),^455^ as well as potentially other bioactive I3P products.^436^

### Tryptamine Pathway

Tryptamine (Try) is a tryptophan-derived indole formed through the decarboxylation of Trp through bacterial enzyme tryptophan decarboxylase (TDC).^457^ This pathway generates Try as both a biologically active signaling molecule and a metabolic precursor that feeds into the indole-3–acetamide pathway via indole-3-acetaldehyde (I3AAld).^458^ Structurally, Try closely resembles 5-hydroxytryptamine (serotonin), allowing it to activate serotonin 5-HT4 receptors in the colon. Through these receptors, Try can enhance ionic flux across the intestinal epithelium and stimulate fluid secretion in the colon, thereby promoting gastrointestinal motility and luminal transit.^459,460^ In the CNS, Try functions as a neuromodulator, facilitating the release of serotonin, dopamine, and norepinephrine at presynaptic terminals, thereby influencing gut-brain signaling.^461–463^

Try is metabolized to I3AAld preferentially by monoamine oxidase-A (MAO-A), although MAO-B has some detectable activity.^464,465^ In addition, I3P can feed into the tryptamine pathway, as microbial I3P is converted to I3AAld by the microbial enzyme indole-3-pyruvate decarboxylase (*ipdC*).^466^ By containing an aldehyde moiety, I3AAld has the potential to react covalently with cellular nucleophiles such as amines and thiols and therefore regulate the activity of redox-sensitive enzymes and transcriptional regulators.^166^ However, most studies on aldehyde-containing indoles have been performed with indole-3-aldehyde (I3Ald, aka indole-carboxaldehyde).

### Indole-3–Acetamide Pathway

In this pathway, the bacterial enzyme tryptophan-2-monooxygenase (TMO) catalyzes the oxidative decarboxylation of Trp to indole-3–acetamide (I3AM), which can then be hydrolyzed by I3AM hydrolase (AMI1) to form indole-3-acetic acid (I3AA).^466,467^ In the I3P pathway, I3P can be decarboxylated by bacterial indole-3-pyruvate decarboxylase (*ipdC*) and further processed by ferredoxin oxido-reductases B and C (*ProB* and *C*) and flavin-containing monooxygenases (FMOs) to produce I3AA.^3,447,468,469^ Similarly, in the Try pathway, I3AAld is oxidized by I3AAld dehydrogenase (ALDH) to produce I3AA.^470^ Functionally, I3AA has been proposed to impact diverse physiological and pathophysiological processes, predominantly via AhR-dependent mechanisms.^471^ However, a detailed analysis of I3AA’s ability to act as an agonist of the human AhR ortholog failed to show significant activity at physiologically relevant concentrations.^421^ Notably, some of the biological effects of I3AA involve interactions between AhR and other transcription factors and signaling proteins, such as p38 MAPK-dependent NF-κB p50 nuclear translocation, without requiring direct binding of AhR to the promoter of target genes.^472–474^ These findings support the notion that traditional AhR agonism might not be required for indoles, or other ligands, to promote AhR-dependent effects. Furthermore, these effects can be mediated by the coactivation of additional signaling pathways, highlighting the context-dependent nature of AhR signaling.^37^ Finally, as with I3PA, I3AA has been shown to activate the human PXR in the presence of indole, but the biological implications of this finding have not been explored.^423^

I3AA can be further converted into either 3-methylindole (skatole), indole-3-aldehyde (I3Ald), or indole-3-carbinol (I3C) by bacterial heme peroxidases.^3^ In the Lactobacillus strain, a dominant component of the human intestinal microbiome, I3AA is metabolized by an oxygen-dependent dye-decolorizing peroxidase termed LfDyP.^475^ More specifically, I3AA is first oxidized by one electron to generate an I3AA^•+^ intermediate that spontaneously decarboxylates to a skatole radical. This unstable product can, in turn, be reduced to skatole by an unspecified electron donor or react with an additional oxygen molecule to generate two possible peroxyl radical-containing products. Dimerization of these products generates two different tetroxide intermediates, which then decompose via the Russell mechanism to produce I3Ald, I3C, and other indolic products.^475^

Skatole has been shown to activate AhR, PHR and MAPK signaling as well as p53. In addition, skatole is proposed to induce pulmonary toxicity via cytochrome P450-mediated metabolism, as well as dysbiosis in the human gut, although the contributions of skatole to human pathophysiology are incompletely understood.^476^ In contrast, the biological actions of I3Ald have been studied in greater detail.^477^ Despite being identified as a weak human AhR agonist, I3Ald has been shown to induce AhR activation and stimulate IL-22 production in mice, and to protect against experimental colitis and *Candidiasis* in the gut and vagina.^421,478,479^ Furthermore, exogenous I3Ald was shown to increase goblet cell numbers in the mouse colon via AhR-dependent IL-10 production and to improve colonic histopathology in a model of graft-versus-host disease.^480,481^ Outside its role in the gut, I3Ald has also been shown to alleviate lung inflammation in COPD and promote tumor immunogenicity via AhR-dependent mechanisms in mice.^482,483^

## Concluding Remarks

The catabolism of Trp results in the synthesis of numerous bioactive products that can impact metabolism, neurotransmission, immune responses, and barrier tissue integrity under both physiological and pathological conditions. Furthermore, the dysregulation of specific branches of Trp metabolism has been associated with numerous chronic diseases. Whereas the pathophysiological relevance of Trp metabolism is clear, a main challenge resides in the elucidation of the contributions of individual metabolites and signaling pathways to the observed effects. In this regard, many biological responses to changes in the concentration of a given Trp metabolite or to the engagement of a specific signaling pathway are context-dependent. For example, although increased KYN levels are associated with a higher probability of type 2 diabetes development and renal function impairment, the loss of IDO1 activity in the pancreas is associated with higher type 1 diabetes prevalence. In addition, it is possible that the upregulation of specific branches of Trp metabolism could constitute an adaptation to a disease, which might subsequently contribute to maladaptive responses. Considering this, although KA is often associated with neuroprotective responses through the inhibition of glutamate channels, the levels of this metabolite are elevated in schizophrenia and Alzheimer’s disease. Another example of context-dependent responses is the effects of metabolites such as 3-HK and 3-HAA, which can chelate metals and generate reactive species in an oxygen-dependent manner. Redox-active compounds often elicit hormetic responses in that they can activate cytoprotective gene expression programs at low concentrations but can promote toxicity when these pathways are overwhelmed by higher doses. In this context, whether a given concentration of 3-HK or 3-HAA is cytoprotective or toxic will be dependent on the oxygen tension, availability of chelatable metals, and presence of antioxidant mechanisms in the compartment of interest.

Importantly, these dichotomies are also observed in the context of specific pathway engagement, with outcomes significantly affected by the concomitant presence of inflammation or differences across cell types. In this regard, the activation of AhR by several metabolites in the kynurenine and indole pathways is a clear example. Furthermore, while some responses appear to require canonical AhR activation, as defined by its binding to xenobiotic response elements in the promoters of target genes, in other cases, the downstream responses appear to be regulated via AhR interactions with other transcription factors or regulatory proteins. These observations are further complicated by the often-observed difference between the ability of a given molecule to activate a receptor across species, with some ligands being significantly more potent toward either human or rodent orthologs. As a result, preclinical experiments performed in rodent models may not faithfully reflect the translational potential in humans.

A notable characteristic of many bioactive intermediates in the kynurenine and indole pathways is the possibility of engaging in independent signaling pathways. This is clearly exemplified by the anti-inflammatory actions of Kyn-CKA, which have recently been shown to depend on Nrf2 signaling at low concentrations but become Nrf2-independent as higher concentrations are used. Establishing physiologically relevant concentrations is often challenging, as many effects are compartment-specific and might require supraphysiological supplementation to achieve bioactive concentrations. Furthermore, since most Trp-derived products can be further metabolized by different tissues and by the microbiome upon administration, the identity of the bioactive intermediate(s) is not always readily apparent. Finally, particularly in the case of the kynurenine pathway, many of the proposed bioactive metabolites are unstable, raising the possibility that at least some of the observed biological effects could be due to the presence of bioactive degradation products.

In summary, new mechanisms of cellular signaling associated with Trp metabolism have continued to be discovered. In this regard, we propose that the KP serves as a previously underappreciated source of endogenous electrophilic signaling molecules, with Kyn-CKA representing a newly defined example. Kyn-CKA possesses an electrophilic α,β-unsaturated carbonyl moiety that enables covalent modification of cysteine residues, thereby introducing an additional layer of regulation through post-translational modification of protein targets. While Kyn-CKA is still being characterized in terms of its interactions, it is unlikely to be the sole electrophilic reactive species generated within this metabolic network. Several downstream intermediates of the KP possess redox-active scaffolds capable of generating electrophilic derivatives under oxidative conditions. Defining the chemical identities, formation mechanisms, and signaling targets of these electrophilic metabolites will be essential to fully understanding how tryptophan catabolism contributes to both physiological homeostasis and pathological processes. Together, these findings position tryptophan catabolism not just as a metabolic process but as an integrated signaling network in which receptor-, redox-, and electrophile-mediated pathways converge to shape cellular adaptation.

## References

[1] Palego L., Betti L., Rossi A., Giannaccini G. (2016). Tryptophan
Biochemistry: Structural, Nutritional, Metabolic, and Medical Aspects
in Humans. J. Amino Acids.

[2] Cervenka I., Agudelo L. Z., Ruas J. L. (2017). Kynurenines: Tryptophan’s
metabolites in exercise, inflammation, and mental health. Science.

[3] Li X., Zhang B., Hu Y., Zhao Y. (2021). New Insights Into Gut-Bacteria-Derived
Indole and Its Derivatives in Intestinal and Liver Diseases. Front. Pharmacol..

[4] Sahu A., Gopalakrishnan L., Gaur N., Chatterjee O., Mol P., Modi P. K., Dagamajalu S., Advani J., Jain S., Keshava
Prasad T. S. (2018). The 5-Hydroxytryptamine signaling map: an overview
of serotonin-serotonin receptor mediated signaling network. J. Cell. Commun. Signal..

[5] Van
der Leek A. P., Yanishevsky Y., Kozyrskyj A. L. (2017). The Kynurenine
Pathway As a Novel Link between Allergy and the Gut Microbiome. Front. Immunol..

[6] Ye X., Li H., Anjum K., Zhong X., Miao S., Zheng G., Liu W., Li L. (2022). Dual Role of Indoles Derived From Intestinal Microbiota
on Human Health. Front. Immunol..

[7] Gasaly N., de Vos P., Hermoso M. A. (2021). Impact
of Bacterial Metabolites on
Gut Barrier Function and Host Immunity: A Focus on Bacterial Metabolism
and Its Relevance for Intestinal Inflammation. Front. Immunol..

[8] Zhou Y., Chen Y., He H., Peng M., Zeng M., Sun H. (2023). The role of the indoles
in microbiota-gut-brain axis and potential
therapeutic targets: A focus on human neurological and neuropsychiatric
diseases. Neuropharmacology.

[9] Wei G. Z., Martin K. A., Xing P. Y., Agrawal R., Whiley L., Wood T. K., Hejndorf S., Ng Y. Z., Low J. Z. Y., Rossant J., Nechanitzky R., Holmes E., Nicholson J. K., Tan E. K., Matthews P. M., Pettersson S. (2021). Tryptophan-metabolizing
gut microbes regulate adult neurogenesis via the aryl hydrocarbon
receptor. Proc. Natl. Acad. Sci. U. S. A..

[10] Daut R. A., Fonken L. K. (2019). Circadian regulation of depression: A role for serotonin. Front Neuroendocrinol..

[11] Alex K. D., Pehek E. A. (2007). Pharmacologic mechanisms of serotonergic regulation
of dopamine neurotransmission. Pharmacol. Ther..

[12] Gao K., Mu C.-L., Farzi A., Zhu W.-Y. (2020). Tryptophan Metabolism:
A Link Between the Gut Microbiota and Brain. Adv. Nutr..

[13] Stone T. W., Williams R. O. (2024). Tryptophan metabolism as a ‘reflex’ feature
of neuroimmune communication: Sensor and effector functions for the
indoleamine-2, 3-dioxygenase kynurenine pathway. J. Neurochem..

[14] Theate I., van Baren N., Pilotte L., Moulin P., Larrieu P., Renauld J. C., Herve C., Gutierrez-Roelens I., Marbaix E., Sempoux C., Van den Eynde B. J. (2015). Extensive
profiling of the expression of the indoleamine 2,3-dioxygenase 1 protein
in normal and tumoral human tissues. Cancer
Immunol. Res..

[15] Orabona C., Pallotta M. T., Grohmann U. (2012). Different partners, opposite outcomes:
a new perspective of the immunobiology of indoleamine 2,3-dioxygenase. Mol. Med..

[16] Jusof F. F., Bakmiwewa S. M., Weiser S., Too L. K., Metz R., Prendergast G. C., Fraser S. T., Hunt N. H., Ball H. J. (2017). Investigation
of the Tissue Distribution and Physiological Roles of Indoleamine
2,3-Dioxygenase-2. Int. J. Tryptophan Res..

[17] Uhlen M., Fagerberg L., Hallstrom B. M., Lindskog C., Oksvold P., Mardinoglu A., Sivertsson A., Kampf C., Sjöstedt E., Asplund A., Olsson I. (2015). Tissue-based map of
the human proteome. Science.

[18] Klaessens S., Stroobant V., De Plaen E., Van den Eynde B. J. (2022). Systemic
tryptophan homeostasis. Front. Mol. Biosci..

[19] Stuehr D. J., Dai Y., Biswas P., Sweeny E. A., Ghosh A. (2022). New roles for GAPDH,
Hsp90, and NO in regulating heme allocation and hemeprotein function
in mammals. Biol. Chem..

[20] Wang X., Chen Z., Chen L., Qiu C. (2026). IDO family: the metabolic
crossroads connecting immunity, nerves and tumors. J. Transl. Med..

[21] Capece L., Lewis-Ballester A., Yeh S. R., Estrin D. A., Marti M. A. (2012). Complete
reaction mechanism of indoleamine 2,3-dioxygenase as revealed by QM/MM
simulations. J. Phys. Chem. B..

[22] Chung L. W., Li X., Sugimoto H., Shiro Y., Morokuma K. (2010). ONIOM study on a missing
piece in our understanding of heme chemistry: bacterial tryptophan
2,3-dioxygenase with dual oxidants. J. Am. Chem.
Soc..

[23] Badawy A.
A. (2017). Kynurenine
Pathway of Tryptophan Metabolism: Regulatory and Functional Aspects. Int. J. Tryptophan Res..

[24] Carreño M., Pires M. F., Woodcock S. R., Brzoska T., Ghosh S., Salvatore S. R., Chang F., Khoo N. K. H., Dunn M., Connors N. (2022). Immunomodulatory actions of a kynurenine-derived endogenous
electrophile. Sci. Adv..

[25] Marszalek-Grabska M., Walczak K., Gawel K., Wicha-Komsta K., Wnorowska S., Wnorowski A., Turski W. A. (2021). Kynurenine emerges
from the shadows - Current knowledge on its fate and function. Pharmacol. Ther..

[26] Mor A., Tankiewicz-Kwedlo A., Krupa A., Pawlak D. (2021). Role of Kynurenine
Pathway in Oxidative Stress during Neurodegenerative Disorders. Cells.

[27] Speciale C., Hares K., Schwarcz R., Brookes N. (1989). High-affinity
uptake
of L-kynurenine by a Na ± independent transporter of neutral
amino acids in astrocytes. J. Neurosci..

[28] Liu Y., Liang X., Dong W., Fang Y., Lv J., Zhang T., Fiskesund R., Xie J., Liu J., Yin X. (2018). Tumor-Repopulating Cells
Induce PD-1 Expression in
CD8­(+) T Cells by Transferring Kynurenine and AhR Activation. Cancer Cell..

[29] Sinclair L. V., Neyens D., Ramsay G., Taylor P. M., Cantrell D. A. (2018). Single
cell analysis of kynurenine and System L amino acid transport in T
cells. Nat. Commun..

[30] Fiore A., Zeitler L., Russier M., Gross A., Hiller M. K., Parker J. L., Stier L., Kocher T., Newstead S., Murray P. J. (2022). Kynurenine importation by SLC7A11
propagates anti-ferroptotic
signaling. Mol. Cell.

[31] Haidar R., Shabo R., Moeser M., Luch A., Kugler J. (2023). The nuclear
entry of the aryl hydrocarbon receptor (AHR) relies on the first nuclear
localization signal and can be negatively regulated through IMPalpha/beta
specific inhibitors. Sci. Rep..

[32] Perdew G. H. (1988). Association
of the Ah receptor with the 90-kDa heat shock protein. J. Biol. Chem..

[33] Kazlauskas A., Poellinger L., Pongratz I. (1999). Evidence that the co-chaperone
p23
regulates ligand responsiveness of the dioxin (Aryl hydrocarbon) receptor. J. Biol. Chem..

[34] Meyer B. K., Pray-Grant M. G., Vanden Heuvel J. P., Perdew G. H. (1998). Hepatitis B virus
X-associated protein 2 is a subunit of the unliganded aryl hydrocarbon
receptor core complex and exhibits transcriptional enhancer activity. Mol. Cell. Biol..

[35] Tsuji N., Fukuda K., Nagata Y., Okada H., Haga A., Hatakeyama S., Yoshida S., Okamoto T., Hosaka M., Sekine K., Ohtaka K., Yamamoto S., Otaka M., Grave E., Itoh H. (2014). The activation mechanism of the aryl
hydrocarbon receptor (AhR) by molecular chaperone HSP90. FEBS Open Bio..

[36] Schulte K. W., Green E., Wilz A., Platten M., Daumke O. (2017). Structural
Basis for Aryl Hydrocarbon Receptor-Mediated Gene Activation. Structure.

[37] Coumoul X., Barouki R., Esser C., Haarmann-Stemmann T., Lawrence B. P., Lehmann J., Moura-Alves P., Murray I. A., Opitz C. A., Perdew G. H., Bourguet W. (2026). The aryl hydrocarbon
receptor: structure, signaling, physiology and pathology. Signal Transduction Targeted Ther..

[38] Gutierrez-Vazquez C., Quintana F. J. (2018). Regulation of the Immune Response
by the Aryl Hydrocarbon
Receptor. Immunity.

[39] Boule L. A., Burke C. G., Jin G. B., Lawrence B. P. (2018). Aryl hydrocarbon
receptor signaling modulates antiviral immune responses: ligand metabolism
rather than chemical source is the stronger predictor of outcome. Sci. Rep..

[40] Riaz F., Pan F., Wei P. (2022). Aryl hydrocarbon
receptor: The master regulator of
immune responses in allergic diseases. Front.
Immunol..

[41] Huang W., Rui K., Wang X., Peng N., Zhou W., Shi X., Lu L., Hu D., Tian J. (2023). The aryl hydrocarbon receptor in
immune regulation and autoimmune pathogenesis. J. Autoimmun..

[42] Sondermann N. C., Fassbender S., Hartung F., Hatala A. M., Rolfes K. M., Vogel C. F. A., Haarmann-Stemmann T. (2023). Functions of the aryl hydrocarbon
receptor (AHR) beyond the canonical AHR/ARNT signaling pathway. Biochem. Pharmacol..

[43] Li K., Li K., He Y., Liang S., Shui X., Lei W. (2023). Aryl hydrocarbon
receptor: A bridge linking immuno-inflammation and metabolism in atherosclerosis. Biochem. Pharmacol..

[44] Vogel C. F., Khan E. M., Leung P. S., Gershwin M. E., Chang W. L., Wu D., Haarmann-Stemmann T., Hoffmann A., Denison M. S. (2014). Cross-talk
between aryl hydrocarbon receptor and the inflammatory response: a
role for nuclear factor-kappaB. J. Biol. Chem..

[45] Granados J. C., Falah K., Koo I., Morgan E. W., Perdew G. H., Patterson A. D., Jamshidi N., Nigam S. K. (2022). AHR is a master
regulator of diverse pathways in endogenous metabolism. Sci. Rep..

[46] Tanos R., Murray I. A., Smith P. B., Patterson A., Perdew G. H. (2012). Role of the Ah receptor in homeostatic
control of fatty
acid synthesis in the liver. Toxicol. Sci..

[47] Yu M., Wang Q., Ma Y., Li L., Yu K., Zhang Z., Chen G., Li X., Xiao W., Xu P., Yang H. (2018). Aryl Hydrocarbon Receptor
Activation Modulates Intestinal
Epithelial Barrier Function by Maintaining Tight Junction Integrity. Int. J. Biol. Sci..

[48] Metidji A., Omenetti S., Crotta S., Li Y., Nye E., Ross E., Li V., Maradana M. R., Schiering C., Stockinger B. (2019). The Environmental Sensor AHR Protects from Inflammatory
Damage by Maintaining Intestinal Stem Cell Homeostasis and Barrier
Integrity. Immunity.

[49] Liu Y., Chen Y., Sha R., Li Y., Xu T., Hu X., Xu L., Xie Q., Zhao B. (2021). A new insight into
the role of aryl hydrocarbon receptor (AhR) in the migration of glioblastoma
by AhR-IL24 axis regulation. Environ. Int..

[50] Diao X., Shang Q., Guo M., Huang Y., Zhang M., Chen X., Liang Y., Sun X., Zhou F., Zhuang J. (2025). Structural basis for
the ligand-dependent activation
of heterodimeric AHR-ARNT complex. Nat. Commun..

[51] Gruszczyk J., Grandvuillemin L., Lai-Kee-Him J., Paloni M., Savva C. G., Germain P., Grimaldi M., Boulahtouf A., Kwong H. S., Bous J. (2022). Cryo-EM structure of
the agonist-bound Hsp90-XAP2-AHR cytosolic complex. Nat. Commun..

[52] Kwong H. S., Paloni M., Grandvuillemin L., Sirounian S., Ancelin A., Lai-Kee-Him J., Grimaldi M., Carivenc C., Lancey C., Ragan T. J., Hesketh E. L., Balaguer P., Barducci A., Gruszczyk J., Bourguet W. (2024). Structural Insights
into the Activation of Human Aryl Hydrocarbon Receptor by the Environmental
Contaminant Benzo­[a]­pyrene and Structurally Related Compounds. J. Mol. Biol..

[53] Badawy A. A.-B., Dawood S. (2024). Molecular Insights into the Interaction of Tryptophan
Metabolites with the Human Aryl Hydrocarbon Receptor in Silico: Tryptophan
as Antagonist and no Direct Involvement of Kynurenine. Front Biosci..

[54] Seok S. H., Ma Z. X., Feltenberger J. B., Chen H., Chen H., Scarlett C., Lin Z., Satyshur K. A., Cortopassi M., Jefcoate C. R., Ge Y., Tang W., Bradfield C. A., Xing Y. (2018). Trace derivatives of
kynurenine potently activate the aryl hydrocarbon
receptor (AHR. J. Biol. Chem..

[55] Avilla M. N., Malecki K. M. C., Hahn M. E., Wilson R. H., Bradfield C. A. (2020). The Ah
Receptor: Adaptive Metabolism, Ligand Diversity, and the Xenokine
Model. Chem. Res. Toxicol..

[56] Feng J., Carreno M., Jung H., Dayalan Naidu S., Arroyo-Diaz N., Ang A. D., Kulkarni B., Kisielewski D., Suzuki T., Yamamoto M., Hayes J. D., Honda T., Wilson L., Leon-Ruiz B., Eggler A. L., Vitturi D. A., Dinkova-Kostova A. T. (2026). The electrophilic
metabolite of kynurenine, kynurenine-CKA,
requires C151 in Keap1 to derepress Nrf2. Redox
Biol..

[57] Mezrich J. D., Fechner J. H., Zhang X., Johnson B. P., Burlingham W. J., Bradfield C. A. (2010). An interaction
between kynurenine and the aryl hydrocarbon
receptor can generate regulatory T cells. J.
Immunol..

[58] Nguyen N. T., Kimura A., Nakahama T., Chinen I., Masuda K., Nohara K., Fujii-Kuriyama Y., Kishimoto T. (2010). Aryl hydrocarbon
receptor negatively regulates dendritic cell immunogenicity via a
kynurenine-dependent mechanism. Proc. Natl.
Acad. Sci. U. S. A..

[59] Bessede A., Gargaro M., Pallotta M. T., Matino D., Servillo G., Brunacci C., Bicciato S., Mazza E. M., Macchiarulo A., Vacca C., Iannitti R., Tissi L., Volpi C., Belladonna M. L., Orabona C., Bianchi R., Lanz T. V., Platten M., Della Fazia M. A., Piobbico D., Zelante T., Funakoshi H., Nakamura T., Gilot D., Denison M. S., Guillemin G. J., DuHadaway J. B., Prendergast G. C., Metz R., Geffard M., Boon L., Pirro M., Iorio A., Veyret B., Romani L., Grohmann U., Fallarino F., Puccetti P. (2014). Aryl hydrocarbon receptor control
of a disease tolerance defence pathway. Nature.

[60] Gargaro M., Scalisi G., Manni G., Briseno C. G., Bagadia P., Durai V., Theisen D. J., Kim S., Castelli M., Xu C. A. (2022). Indoleamine 2,3-dioxygenase
1 activation in mature
cDC1 promotes tolerogenic education of inflammatory cDC2 via metabolic
communication. Immunity.

[61] Schlichtner S., Yasinska I. M., Klenova E., Abooali M., Lall G. S., Berger S. M., Ruggiero S., Cholewa D., Milosevic M., Gibbs B. F. (2023). L-Kynurenine
participates in cancer immune
evasion by downregulating hypoxic signaling in T lymphocytes. Oncoimmunology.

[62] Campesato L. F., Budhu S., Tchaicha J., Weng C. H., Gigoux M., Cohen I. J., Redmond D., Mangarin L., Pourpe S., Liu C. (2020). Blockade
of the AHR restricts a Treg-macrophage suppressive
axis induced by L-Kynurenine. Nat. Commun..

[63] Rad
Pour S., Morikawa H., Kiani N. A., Yang M., Azimi A., Shafi G., Shang M., Baumgartner R., Ketelhuth D. F. J., Kamleh M. A. (2019). Exhaustion of CD4+ T-cells
mediated by the Kynurenine Pathway in Melanoma. Sci. Rep..

[64] Boulet M. M., Chevrier G., Grenier-Larouche T., Pelletier M., Nadeau M., Scarpa J., Prehn C., Marette A., Adamski J., Tchernof A. (2015). Alterations of plasma
metabolite
profiles related to adipose tissue distribution and cardiometabolic
risk. Am. J. Physiol. Endocrinol. Metab..

[65] Wolowczuk I., Hennart B., Leloire A., Bessede A., Soichot M., Taront S., Caiazzo R., Raverdy V., Pigeyre M., Consortium A. (2012). Tryptophan metabolism activation by indoleamine
2,3-dioxygenase in adipose tissue of obese women: an attempt to maintain
immune homeostasis and vascular tone. Am. J.
Physiol. Regul. Integr. Comp. Physiol..

[66] Pertovaara M., Raitala A., Juonala M., Lehtimaki T., Huhtala H., Oja S. S., Jokinen E., Viikari J. S., Raitakari O. T., Hurme M. (2007). Indoleamine 2,3-dioxygenase enzyme
activity correlates with risk factors for atherosclerosis: the Cardiovascular
Risk in Young Finns Study. Clin. Exp. Immunol..

[67] Niinisalo P., Raitala A., Pertovaara M., Oja S. S., Lehtimaki T., Kahonen M., Reunanen A., Jula A., Moilanen L., Kesaniemi Y. A., Nieminen M. S., Hurme M. (2008). Indoleamine 2,3-dioxygenase
activity associates with cardiovascular risk factors: the Health 2000
study. Scand J. Clin Lab Invest..

[68] Huang T., Song J., Gao J., Cheng J., Xie H., Zhang L., Wang Y. H., Gao Z., Wang Y., Wang X. (2022). Adipocyte-derived kynurenine
promotes obesity and insulin
resistance by activating the AhR/STAT3/IL-6 signaling. Nat. Commun..

[69] Laurans L., Venteclef N., Haddad Y., Chajadine M., Alzaid F., Metghalchi S., Sovran B., Denis R. G. P., Dairou J., Cardellini M., Moreno-Navarrete J. M., Straub M., Jegou S., McQuitty C., Viel T., Esposito B., Tavitian B., Callebert J., Luquet S. H., Federici M., Fernandez-Real J. M., Burcelin R., Launay J. M., Tedgui A., Mallat Z., Sokol H., Taleb S. (2018). Genetic deficiency of indoleamine
2,3-dioxygenase promotes gut microbiota-mediated metabolic health. Nat. Med..

[70] Liu J. J., Ching J., Wee H. N., Liu S., Gurung R. L., Lee J. M. Y., Zheng H., Lee L. S., Ang K., Shao Y. M. (2023). Plasma Tryptophan-Kynurenine Pathway Metabolites
and
Risk for Progression to End-Stage Kidney Disease in Patients With
Type 2 Diabetes. Diabetes Care.

[71] Rebnord E. W., Strand E., Midttun O., Svingen G. F. T., Christensen M. H. E., Ueland P. M., Mellgren G., Njolstad P. R., Tell G. S., Nygard O. K., Pedersen E. R. (2017). The kynurenine: tryptophan ratio
as a predictor of incident type 2 diabetes mellitus in individuals
with coronary artery disease. Diabetologia.

[72] Rhee E. P., Clish C. B., Ghorbani A., Larson M. G., Elmariah S., McCabe E., Yang Q., Cheng S., Pierce K., Deik A., Souza A. L., Farrell L., Domos C., Yeh R. W., Palacios I., Rosenfield K., Vasan R. S., Florez J. C., Wang T. J., Fox C. S., Gerszten R. E. (2013). A combined epidemiologic and metabolomic
approach improves
CKD prediction. J. Am. Soc. Nephrol..

[73] Anquetil F., Mondanelli G., Gonzalez N., Rodriguez Calvo T., Zapardiel Gonzalo J., Krogvold L., Dahl-Jorgensen K., Van den Eynde B., Orabona C., Grohmann U., von Herrath M. G. (2018). Loss of
IDO1 Expression From Human Pancreatic beta-Cells Precedes Their Destruction
During the Development of Type 1 Diabetes. Diabetes.

[74] Pallotta M. T., Orabona C., Bianchi R., Vacca C., Fallarino F., Belladonna M. L., Volpi C., Mondanelli G., Gargaro M., Allegrucci M., Talesa V. N., Puccetti P., Grohmann U. (2014). Forced IDO1 expression
in dendritic cells restores
immunoregulatory signalling in autoimmune diabetes. J. Cell. Mol. Med.

[75] Orabona C., Mondanelli G., Pallotta M. T., Carvalho A., Albini E., Fallarino F., Vacca C., Volpi C., Belladonna M. L., Berioli M. G. (2018). Deficiency of immunoregulatory indoleamine
2,3-dioxygenase 1in juvenile diabetes. JCI Insight.

[76] Mondanelli G., Albini E., Pallotta M. T., Volpi C., Chatenoud L., Kuhn C., Fallarino F., Matino D., Belladonna M. L., Bianchi R. (2017). The Proteasome
Inhibitor Bortezomib Controls Indoleamine
1 Breakdown and Restores Immune Regulation in Autoimmune Diabetes. Front. Immunol..

[77] Takikawa O., Yoshida R., Kido R., Hayaishi O. (1986). Tryptophan degradation
in mice initiated by indoleamine 2,3-dioxygenase. J. Biol. Chem.

[78] Schefold J. C., Zeden J. P., Fotopoulou C., von Haehling S., Pschowski R., Hasper D., Volk H. D., Schuett C., Reinke P. (2009). Increased indoleamine 2,3-dioxygenase
(IDO) activity
and elevated serum levels of tryptophan catabolites in patients with
chronic kidney disease: a possible link between chronic inflammation
and uraemic symptoms. Nephrol. Dial. Transplant.

[79] Vanholder R., De Smet R., Glorieux G., Argiles A., Baurmeister U., Brunet P., Clark W., Cohen G., De Deyn P. P., Deppisch R., Descamps-Latscha B., Henle T., Jorres A., Lemke H. D., Massy Z. A., Passlick-Deetjen J., Rodriguez M., Stegmayr B., Stenvinkel P., Tetta C., Wanner C., Zidek W. (2003). Review on uremic toxins:
classification, concentration, and interindividual variability. And European Uremic Toxin Work, G..

[80] Castro-Portuguez R., Sutphin G. L. (2020). Kynurenine pathway,
NAD­(+) synthesis, and mitochondrial
function: Targeting tryptophan metabolism to promote longevity and
healthspan. Exp. Gerontol..

[81] Joisten N., Ruas J. L., Braidy N., Guillemin G. J., Zimmer P. (2021). The kynurenine pathway in chronic diseases: a compensatory
mechanism or a driving force?. Trends Mol. Med..

[82] Wirthgen E., Hoeflich A., Rebl A., Gunther J. (2018). Kynurenic Acid: The
Janus-Faced Role of an Immunomodulatory Tryptophan Metabolite and
Its Link to Pathological Conditions. Front.
Immunol..

[83] DiNatale B. C., Murray I. A., Schroeder J. C., Flaveny C. A., Lahoti T. S., Laurenzana E. M., Omiecinski C. J., Perdew G. H. (2010). Kynurenic acid is
a potent endogenous aryl hydrocarbon receptor ligand that synergistically
induces interleukin-6 in the presence of inflammatory signaling. Toxicol. Sci..

[84] Baran H., Amann G., Lubec B., Lubec G. (1997). Kynurenic acid and
kynurenine aminotransferase in heart. Pediatr.
Res..

[85] Okuno E., Nakamura M., Schwarcz R. (1991). Two kynurenine aminotransferases
in human brain. Brain Res..

[86] Guidetti P., Okuno E., Schwarcz R. (1997). Characterization
of rat brain kynurenine
aminotransferases I and II. J. Neurosci. Res..

[87] Swartz K. J., Matson W. R., MacGarvey U., Ryan E. A., Beal M. F. (1990). Measurement
of kynurenic acid in mammalian brain extracts and cerebrospinal fluid
by high-performance liquid chromatography with fluorometric and coulometric
electrode array detection. Anal. Biochem..

[88] Rejdak R., Zarnowski T., Turski W. A., Kocki T., Zagorski Z., Zrenner E., Schuettauf F. (2003). Alterations of kynurenic acid content
in the retina in response to retinal ganglion cell damage. Vision Res..

[89] O’Reilly K., O’Farrell K., Midttun O., Rakovets Y., David-Bercholz J., Harkin A. (2021). Kynurenic Acid Protects Against Reactive
Glial-associated
Reductions in the Complexity of Primary Cortical Neurons. J. Neuroimmune Pharmacol..

[90] Guillemin G. J., Cullen K. M., Lim C. K., Smythe G. A., Garner B., Kapoor V., Takikawa O., Brew B. J. (2007). Characterization
of the kynurenine pathway in human neurons. J. Neurosci..

[91] Walczak K., Wnorowski A., Turski W. A., Plech T. (2020). Kynurenic acid and
cancer: facts and controversies. Cell. Mol.
Life Sci..

[92] Stazka J., Luchowski P., Wielosz M., Kleinrok Z., Urbanska E. M. (2002). Endothelium-dependent
production and liberation of kynurenic acid by rat aortic rings exposed
to L-kynurenine. Eur. J. Pharmacol..

[93] Matysik-Wozniak A., Junemann A., Turski W. A., Wnorowski A., Jozwiak K., Paduch R., Okuno E., Moneta-Wielgos J., Choragiewicz T., Maciejewski R. (2017). The presence of kynurenine
aminotransferases in the human cornea: Evidence from bioinformatics
analysis of gene expression and immunohistochemical staining. Mol. Vis..

[94] Walczak K., Dabrowski W., Langner E., Zgrajka W., Pilat J., Kocki T., Rzeski W., Turski W. A. (2011). Kynurenic acid synthesis
and kynurenine aminotransferases expression in colon derived normal
and cancer cells. Scand. J. Gastroenterol..

[95] Liu J. J., Raynal S., Bailbe D., Gausseres B., Carbonne C., Autier V., Movassat J., Kergoat M., Portha B. (2015). Expression of the kynurenine pathway
enzymes in the
pancreatic islet cells. Activation by cytokines and glucolipotoxicity. Biochim. Biophys. Acta..

[96] Jones S. P., Franco N. F., Varney B., Sundaram G., Brown D. A., de Bie J., Lim C. K., Guillemin G. J., Brew B. J. (2015). Expression of the Kynurenine Pathway in Human Peripheral
Blood Mononuclear Cells: Implications for Inflammatory and Neurodegenerative
Disease. PLoS One.

[97] Agudelo L. Z., Femenia T., Orhan F., Porsmyr-Palmertz M., Goiny M., Martinez-Redondo V., Correia J. C., Izadi M., Bhat M., Schuppe-Koistinen I., Pettersson A. T., Ferreira D. M. S., Krook A., Barres R., Zierath J. R., Erhardt S., Lindskog M., Ruas J. L. (2014). Skeletal
muscle
PGC-1alpha1 modulates kynurenine metabolism and mediates resilience
to stress-induced depression. Cell.

[98] Hartai Z., Juhasz A., Rimanoczy A., Janaky T., Donko T., Dux L., Penke B., Toth G. K., Janka Z., Kalman J. (2007). Decreased
serum and red blood cell kynurenic acid levels in Alzheimer’s
disease. Neurochem. Int..

[99] Milart P., Urbanska E. M., Turski W. A., Paszkowski T., Sikorski R. (1999). Intrapartum levels of endogenous
glutamate antagonist
kynurenic acid in amniotic fluid, umbilical and maternal blood. Neurosci. Res. Commun..

[100] Milart P., Paluszkiewicz P., Dobrowolski P., Tomaszewska E., Smolinska K., Debinska I., Gawel K., Walczak K., Bednarski J., Turska M. (2019). Kynurenic
acid as the neglected ingredient of commercial baby formulas. Sci. Rep..

[101] Parada-Turska J., Rzeski W., Zgrajka W., Majdan M., Kandefer-Szerszen M., Turski W. (2006). Kynurenic acid, an
endogenous constituent
of rheumatoid arthritis synovial fluid, inhibits proliferation of
synoviocytes in vitro. Rheumatol. Int..

[102] Kuc D., Rahnama M., Tomaszewski T., Rzeski W., Wejksza K., Urbanik-Sypniewska T., Parada-Turska J., Wielosz M., Turski W. A. (2006). Kynurenic
acid in human saliva–does it influence oral microflora?. Pharmacol. Rep..

[103] Paluszkiewicz P., Zgrajka W., Saran T., Schabowski J., Piedra J. L., Fedkiv O., Rengman S., Pierzynowski S. G., Turski W. A. (2009). High concentration of kynurenic acid in bile and pancreatic
juice. Amino Acids..

[104] Fathi M., Vakili K., Yaghoobpoor S., Tavasol A., Jazi K., Hajibeygi R., Shool S., Sodeifian F., Klegeris A., McElhinney A., Tavirani M. R., Sayehmiri F. (2022). Dynamic changes in metabolites of
the kynurenine pathway in Alzheimer’s disease, Parkinson’s
disease, and Huntington’s disease: A systematic Review and
meta-analysis. Front. Immunol..

[105] Parsons C. G., Danysz W., Quack G., Hartmann S., Lorenz B., Wollenburg C., Baran L., Przegalinski E., Kostowski W., Krzascik P., Chizh B., Headley P. M. (1997). Novel systemically
active antagonists of the glycine site of the N-methyl-D-aspartate
receptor: electrophysiological, biochemical and behavioral characterization. J. Pharmacol. Exp. Ther..

[106] Stone T. W. (1993). Neuropharmacology of quinolinic and kynurenic acids. Pharmacol. Rev..

[107] Stone T. W. (2020). Does kynurenic acid act on nicotinic receptors? An
assessment of the evidence. J. Neurochem..

[108] Wu H. Q., Guidetti P., Goodman J. H., Varasi M., Ceresoli-Borroni G., Speciale C., Scharfman H. E., Schwarcz R. (2000). Kynurenergic manipulations influence excitatory synaptic
function and excitotoxic vulnerability in the rat hippocampus in vivo. Neuroscience.

[109] St’astny F., Lisy V., Mares V., Lisa V., Balcar V. J., Santamaria A. (2004). Quinolinic
acid induces NMDA receptor-mediated
lipid peroxidation in rat brain microvessels. Redox Rep..

[110] Stone T. W., Darlington L. G. (2013). The kynurenine pathway as a therapeutic
target in cognitive and neurodegenerative disorders. Br. J. Pharmacol..

[111] Kozak R., Campbell B. M., Strick C. A., Horner W., Hoffmann W. E., Kiss T., Chapin D. S., McGinnis D., Abbott A. L., Roberts B. M., Fonseca K., Guanowsky V., Young D. A., Seymour P. A., Dounay A., Hajos M., Williams G. V., Castner S. A. (2014). Reduction of brain kynurenic acid
improves cognitive function. J. Neurosci..

[112] Prescott C., Weeks A. M., Staley K. J., Partin K. M. (2006). Kynurenic
acid has a dual action on AMPA receptor responses. Neurosci. Lett..

[113] Hilmas C., Pereira E. F., Alkondon M., Rassoulpour A., Schwarcz R., Albuquerque E. X. (2001). The brain metabolite kynurenic acid
inhibits alpha7 nicotinic receptor activity and increases non-alpha7
nicotinic receptor expression: physiopathological implications. J. Neurosci..

[114] Albuquerque E. X., Schwarcz R. (2013). Kynurenic acid as an
antagonist of
alpha7 nicotinic acetylcholine receptors in the brain: facts and challenges. Biochem. Pharmacol..

[115] Schwarcz R., Stone T. W. (2017). The kynurenine pathway and the brain:
Challenges, controversies and promises. Neuropharmacology.

[116] Knapskog A. B., Aksnes M., Edwin T. H., Ueland P. M., Ulvik A., Fang E. F., Eldholm R. S., Halaas N. B., Saltvedt I., Giil L. M., Watne L. O. (2023). Higher
concentrations
of kynurenic acid in CSF are associated with the slower clinical progression
of Alzheimer’s disease. Alzheimers Dement..

[117] Miranda A. F., Boegman R. J., Beninger R. J., Jhamandas K. (1997). Protection
against quinolinic acid-mediated excitotoxicity in nigrostriatal dopaminergic
neurons by endogenous kynurenic acid. Neuroscience.

[118] Ogawa T., Matson W. R., Beal M. F., Myers R. H., Bird E. D., Milbury P., Saso S. (1992). Kynurenine
pathway
abnormalities in Parkinson’s disease. Neurology.

[119] Sorgdrager F. J. H., Vermeiren Y., Van Faassen M., van der Ley C., Nollen E. A. A., Kema I. P., De Deyn P. P. (2019). Age- and
disease-specific changes of the kynurenine pathway in Parkinson’s
and Alzheimer’s disease. J. Neurochem..

[120] Bondulich M. K., Fan Y., Song Y., Giorgini F., Bates G. P. (2021). Ablation of kynurenine 3-monooxygenase
rescues plasma
inflammatory cytokine levels in the R6/2 mouse model of Huntington’s
disease. Sci. Rep..

[121] Beaumont V., Mrzljak L., Dijkman U., Freije R., Heins M., Rassoulpour A., Tombaugh G., Gelman S., Bradaia A., Steidl E., Gleyzes M., Heikkinen T., Lehtimaki K., Puolivali J., Kontkanen O., Javier R. M., Neagoe I., Deisemann H., Winkler D., Ebneth A., Khetarpal V., Toledo-Sherman L., Dominguez C., Park L. C., Munoz-Sanjuan I. (2016). The novel
KMO inhibitor CHDI-340246 leads to a restoration of electrophysiological
alterations in mouse models of Huntington’s disease. Exp. Neurol..

[122] Zwilling D., Huang S. Y., Sathyasaikumar K. V., Notarangelo F. M., Guidetti P., Wu H. Q., Lee J., Truong J., Andrews-Zwilling Y., Hsieh E. W., Louie J. Y., Wu T., Scearce-Levie K., Patrick C., Adame A., Giorgini F., Moussaoui S., Laue G., Rassoulpour A., Flik G., Huang Y., Muchowski J. M., Masliah E., Schwarcz R., Muchowski P. J. (2011). Kynurenine
3-monooxygenase inhibition in blood ameliorates neurodegeneration. Cell.

[123] Khetarpal V., Herbst T., Dominguez C., Munoz-Sanjuan I., Sampaio C., Marks B., Miller D. L., Farnham J., Ledvina A., Anglehart H., Rehmani I., LaFayette A., Spridco N., Langbehn D., Wild E. J., Pacifici R. (2025). Lack of Evidence
for Kynurenine Pathway
Dysfunction in Huntington’s Disease: CSF and Plasma Analyses
from the HDClarity Study. J. Huntingtons. Dis..

[124] Rodrigues F. B., Byrne L. M., Lowe A. J., Tortelli R., Heins M., Flik G., Johnson E. B., De Vita E., Scahill R. I., Giorgini F., Wild E. J. (2021). Kynurenine
pathway
metabolites in cerebrospinal fluid and blood as potential biomarkers
in Huntington’s disease. J. Neurochem..

[125] Wu Y., Zhang P., Fan H., Zhang C., Yu P., Liang X., Chen Y. (2023). GPR35 acts
a dual role and therapeutic
target in inflammation. Front. Immunol..

[126] Nógrádi-Halmi D., Erdélyi-Furka B., Csóré D., Plechl É., Igaz N., Juhász L., Poles M. Z., Nógrádi B., Patai R., Polgár T. F. (2025). Kynurenic Acid Protects Against Myocardial
Ischemia/Reperfusion Injury by Activating GPR35 Receptors and Preserving
Mitochondrial Structure and Function. Biomolecules.

[127] Yang Y., Guan W. (2025). From orphan receptor to inflammation
regulation: The role of GPR35 in the pathogenesis of depression. Biochem. Pharmacol..

[128] Jenkins L., Alvarez-Curto E., Campbell K., de Munnik S., Canals M., Schlyer S., Milligan G. (2011). Agonist activation
of the G protein-coupled receptor GPR35 involves transmembrane domain
III and is transduced via Galpha(1)(3) and beta-arrestin-2. Br. J. Pharmacol..

[129] Jenkins L., Harries N., Lappin J. E., MacKenzie A. E., Neetoo-Isseljee Z., Southern C., McIver E. G., Nicklin S. A., Taylor D. L., Milligan G. (2012). Antagonists of GPR35
display high
species ortholog selectivity and varying modes of action. J. Pharmacol. Exp. Ther..

[130] Gaspar R., Nogradi-Halmi D., Demjan V., Dioszegi P., Igaz N., Vincze A., Pipicz M., Kiricsi M., Vecsei L., Csont T. (2024). Kynurenic
acid protects against ischemia/reperfusion
injury by modulating apoptosis in cardiomyocytes. Apoptosis.

[131] Nogradi-Halmi D., Erdelyi-Furka B., Csore D., Plechl E., Igaz N., Juhasz L., Poles M. Z., Nogradi B., Patai R., Polgar T. F. (2025). Kynurenic Acid Protects
Against Myocardial Ischemia/Reperfusion Injury by Activating GPR35
Receptors and Preserving Mitochondrial Structure and Function. Biomolecules.

[132] Wyant G. A., Yu W., Doulamis I. P., Nomoto R. S., Saeed M. Y., Duignan T., McCully J. D., Kaelin W. G. (2022). Mitochondrial
remodeling and ischemic protection by G protein-coupled receptor 35
agonists. Science.

[133] De Giovanni M., Tam H., Valet C., Xu Y., Looney M. R., Cyster J. G. (2022). GPR35 promotes neutrophil recruitment
in response to serotonin metabolite 5-HIAA. Cell.

[134] Kaya B., Donas C., Wuggenig P., Diaz O. E., Morales R. A., Melhem H., Swiss I. B. D., Hernandez P. P., Kaymak T., Das S. (2020). Lysophosphatidic Acid-Mediated
GPR35 Signaling in CX3CR1­(+) Macrophages Regulates Intestinal Homeostasis. Cell Rep..

[135] Barth M. C., Ahluwalia N., Anderson T. J., Hardy G. J., Sinha S., Alvarez-Cardona J. A., Pruitt I. E., Rhee E. P., Colvin R. A., Gerszten R. E. (2009). Kynurenic
acid triggers firm arrest
of leukocytes to vascular endothelium under flow conditions. J. Biol. Chem..

[136] Sun T., Xie R., He H., Xie Q., Zhao X., Kang G., Cheng C., Yin W., Cong J., Li J., Wang X. (2022). Kynurenic acid ameliorates NLRP3 inflammasome activation
by blocking calcium mobilization via GPR35. Front. Immunol.

[137] Salimi Elizei S., Poormasjedi-Meibod M.
S., Wang X., Kheirandish M., Ghahary A. (2017). Kynurenic acid downregulates IL-17/1L-23
axis in vitro. Mol. Cell. Biochem..

[138] Agudelo L. Z., Ferreira D. M. S., Cervenka I., Bryzgalova G., Dadvar S., Jannig P. R., Pettersson-Klein A. T., Lakshmikanth T., Sustarsic E. G., Porsmyr-Palmertz M. (2018). Kynurenic Acid and Gpr35 Regulate Adipose Tissue Energy Homeostasis
and Inflammation. Cell Metab..

[139] Jung T. W., Park J., Sun J. L., Ahn S. H., Abd El-Aty A. M., Hacimuftuoglu A., Kim H. C., Shim J. H., Shin S., Jeong J. H. (2020). Administration
of kynurenic acid
reduces hyperlipidemia-induced inflammation and insulin resistance
in skeletal muscle and adipocytes. Mol. Cell.
Endocrinol..

[140] Ema M., Ohe N., Suzuki M., Mimura J., Sogawa K., Ikawa S., Fujii-Kuriyama Y. (1994). Dioxin binding
activities of polymorphic
forms of mouse and human arylhydrocarbon receptors. J. Biol. Chem..

[141] Poland A., Palen D., Glover E. (1994). Analysis of the four
alleles of the murine aryl hydrocarbon receptor. Mol. Pharmacol..

[142] Wang G., Cao K., Liu K., Xue Y., Roberts A. I., Li F., Han Y., Rabson A. B., Wang Y., Shi Y. (2025). Correction: Kynurenic acid, an IDO
metabolite, controls TSG-6-mediated immunosuppression of human mesenchymal
stem cells. Cell Death Differ..

[143] Phillips R. S. (2014). Structure and mechanism of kynureninase. Arch. Biochem. Biophys..

[144] Pawlowski T., Pawlak D., Inglot M., Zalewska M., Marciniak D., Bugajska J., Janocha-Litwin J., Malyszczak K. (2021). The role of anthranilic acid in the increase of depressive
symptoms and major depressive disorder during treatment for hepatitis
C with pegylated interferon-alpha2a and oral ribavirin. J. Psychiatry Neurosci..

[145] Mikami E., Goto T., Ohno T., Matsumoto H., Inagaki K., Ishihara H., Nishida M. (2000). Simultaneous analysis
of anthranilic acid derivatives in pharmaceuticals and human urine
by high-performance liquid chromatography with isocratic elution. J. Chromatogr. B: biomed. Sci. Appl..

[146] Oxenkrug G., van der Hart M., Summergrad P. (2015). Elevated anthranilic
acid plasma concentrations in type 1 but not type 2 diabetes mellitus. Integr Mol. Med..

[147] Tankiewicz A., Pawlak D., Pawlak K., Szewc D., Mysliwiec M., Buczko W. (2005). Anthranilic acid-uraemic toxin damaged
red cell’s membrane. Int. Urol. Nephrol..

[148] Varnavas A., Lassiani L., Valenta V. (2000). Synthesis
of new anthranilic
acid dimer derivatives and their evaluation on CCK receptors. Farmaco..

[149] Song S., Sun X., Guo Q., Cui B., Zhu Y., Li X., Zhou J., Zhang L. H., Deng Y. (2022). An anthranilic
acid-responsive transcriptional regulator controls the physiology
and pathogenicity of Ralstonia solanacearum. PLoS Pathog..

[150] Chen Y., Fu Y., Xia Y., Miao Y., Shao J., Xuan W., Liu Y., Xun W., Yan Q., Shen Q., Zhang R. (2024). Trichoderma-secreted anthranilic
acid promotes lateral root development via auxin signaling and RBOHF-induced
endodermal cell wall remodeling. Cell Rep..

[151] Sakurai M., Yamamoto Y., Kanayama N., Hasegawa M., Mouri A., Takemura M., Matsunami H., Miyauchi T., Tokura T., Kimura H. (2020). Serum
Metabolic Profiles of the Tryptophan-Kynurenine Pathway in the high
risk subjects of major depressive disorder. Sci. Rep..

[152] Oxenkrug G., van der Hart M., Roeser J., Summergrad P. (2016). Anthranilic
Acid: A Potential Biomarker and Treatment Target for Schizophrenia. Ann. Psychiatry Ment Health.

[153] Igari T., Tsuchizawa M., Shimamura T. (1987). Alteration
of tryptophan metabolism in the synovial fluid of patients with rheumatoid
arthritis and osteoarthritis. Tohoku J. Exp.
Med..

[154] Platten M., Nollen E. A. A., Rohrig U. F., Fallarino F., Opitz C. A. (2019). Tryptophan metabolism as a common therapeutic target
in cancer, neurodegeneration and beyond. Nat.
Rev. Drug Discovery.

[155] Boros F. A., Vecsei L. (2019). Immunomodulatory Effects of Genetic
Alterations Affecting the Kynurenine Pathway. Front. Immunol..

[156] Munn D. H., Sharma M. D., Baban B., Harding H. P., Zhang Y., Ron D., Mellor A. L. (2005). GCN2 kinase
in T
cells mediates proliferative arrest and anergy induction in response
to indoleamine 2,3-dioxygenase. Immunity.

[157] Pallotta M. T., Orabona C., Volpi C., Vacca C., Belladonna M. L., Bianchi R., Servillo G., Brunacci C., Calvitti M., Bicciato S., Mazza E. M., Boon L., Grassi F., Fioretti M. C., Fallarino F., Puccetti P., Grohmann U. (2011). Indoleamine 2,3-dioxygenase is a
signaling protein in long-term tolerance by dendritic cells. Nat. Immunol..

[158] Minhas P. S., Liu L., Moon P. K., Joshi A. U., Dove C., Mhatre S., Contrepois K., Wang Q., Lee B. A., Coronado M., Bernstein D., Snyder M. P., Migaud M., Majeti R., Mochly-Rosen D., Rabinowitz J. D., Andreasson K. I. (2019). Macrophage
de novo NAD­(+) synthesis
specifies immune function in aging and inflammation. Nat. Immunol..

[159] Stone T. W., Stoy N., Darlington L. G. (2013). An expanding
range of targets for kynurenine metabolites of tryptophan. Trends Pharmacol. Sci..

[160] Garner B., Vazquez S., Griffith R., Lindner R. A., Carver J. A., Truscott R. J. (1999). Identification of glutathionyl-3-hydroxykynurenine
glucoside as a novel fluorophore associated with aging of the human
lens. J. Biol. Chem..

[161] Hood B. D., Garner B., Truscott R. J. (1999). Human lens
coloration
and aging. Evidence for Crystallin modification by the major ultraviolet
filter, 3-hydroxy-kynurenine O-beta-D-glucoside. J. Biol. Chem..

[162] Kopylova L. V., Snytnikova O. A., Chernyak E. I., Morozov S. V., Forbes M. D., Tsentalovich Y. P. (2009). Kinetics and mechanism of thermal
decomposition of kynurenines and biomolecular conjugates: ramifications
for the modification of mammalian eye lens proteins. Org. Biomol. Chem..

[163] Kopylova L. V., Snytnikova O. A., Chernyak E. I., Morozov S. V., Tsentalovich Y. P. (2007). UV filter
decomposition. A study of reactions of 4-(2-aminophenyl)-4-oxocrotonic
acid with amino acids and antioxidants present in the human lens. Exp. Eye Res..

[164] Taylor L. M., Andrew Aquilina J., Jamie J. F., Truscott R. J. (2002). UV filter
instability: consequences for the human lens. Exp. Eye Res..

[165] Vazquez S., Aquilina J. A., Jamie J. F., Sheil M. M., Truscott R. J. (2002). Novel protein modification by kynurenine in human lenses. J. Biol. Chem..

[166] Schopfer F. J., Cipollina C., Freeman B. A. (2011). Formation and signaling
actions of electrophilic lipids. Chem. Rev..

[167] Parvez S., Long M. J. C., Poganik J. R., Aye Y. (2018). Redox Signaling
by Reactive Electrophiles and Oxidants. Chem.
Rev..

[168] Schopfer, F. J. ; Vitturi, D. A. Chapter 9 - Thiol modification and signaling by biological electrophiles. In Redox Chemistry and Biology of Thiols, Alvarez, B. ; Comini, M. A. ; Salinas, G. ; Trujillo, M. , Eds.; Elsevier, 2022; pp. 177–196.

[169] Greaney A. J., Maier N. K., Leppla S. H., Moayeri M. (2016). Sulforaphane
inhibits multiple inflammasomes through an Nrf2-independent mechanism. J. Leukoc. Biol..

[170] Long M. J. C., Aye Y. (2017). Privileged Electrophile Sensors:
A Resource for Covalent Drug Development. Cell
Chem. Biol..

[171] Schopfer F. J., Vitturi D. A., Jorkasky D. K., Freeman B. A. (2018). Nitro-fatty
acids: New drug candidates for chronic inflammatory and fibrotic diseases. Nitric Oxide.

[172] Bauer R. A. (2015). Covalent inhibitors in drug discovery: from accidental
discoveries to avoided liabilities and designed therapies. Drug Discov. Today.

[173] Cuadrado A., Manda G., Hassan A., Alcaraz M. J., Barbas C., Daiber A., Ghezzi P., Leon R., Lopez M. G., Oliva B., Pajares M., Rojo A. I., Robledinos-Anton N., Valverde A. M., Guney E., Schmidt H. (2018). Transcription
Factor NRF2 as a Therapeutic Target for Chronic Diseases: A Systems
Medicine Approach. Pharmacol. Rev..

[174] Hayes J. D., Dayalan Naidu S., Dinkova-Kostova A. T. (2025). Regulating
Nrf2 activity: ubiquitin ligases and signaling molecules in redox
homeostasis. Trends Biochem. Sci..

[175] Dinkova-Kostova A. T., Hakomaki H., Levonen A. L. (2024). Electrophilic
metabolites
targeting the KEAP1/NRF2 partnership. Curr.
Opin. Chem. Biol..

[176] Turell L., Vitturi D. A., Coitiño E. L., Lebrato L., Möller M. N., Sagasti C., Salvatore S. R., Woodcock S. R., Alvarez B., Schopfer F. J. (2017). The Chemical Basis
of Thiol Addition to Nitro-Conjugated Linoleic Acid, a Protective
Cell-Signaling Lipid. J. Biol. Chem..

[177] Rudolph V., Schopfer F. J., Khoo N. K., Rudolph T. K., Cole M. P., Woodcock S. R., Bonacci G., Groeger A. L., Golin-Bisello F., Chen C. S., Baker P. R., Freeman B. A. (2009). Nitro-fatty
acid metabolome: saturation, desaturation, beta-oxidation, and protein
adduction. J. Biol. Chem..

[178] Salvatore S. R., Vitturi D. A., Baker P. R., Bonacci G., Koenitzer J. R., Woodcock S. R., Freeman B. A., Schopfer F. J. (2013). Characterization
and quantification of endogenous fatty acid nitroalkene metabolites
in human urine. J. Lipid Res..

[179] Salvatore S. R., Vitturi D. A., Fazzari M., Jorkasky D. K., Schopfer F. J. (2017). Evaluation of 10-Nitro Oleic Acid
Bio-Elimination in
Rats and Humans. Sci. Rep..

[180] Vitturi D. A., Chen C. S., Woodcock S. R., Salvatore S. R., Bonacci G., Koenitzer J. R., Stewart N. A., Wakabayashi N., Kensler T. W., Freeman B. A., Schopfer F. J. (2013). Modulation of nitro-fatty
acid signaling: prostaglandin reductase-1 is a nitroalkene reductase. J. Biol. Chem..

[181] Hanna P. E., Anders M. W. (2019). The mercapturic acid pathway. Crit. Rev. Toxico..

[182] Schopfer, F. J. ; Vitturi, D. A. Thiol modification and signaling by biological electrophiles. In Redox Chemistry and Biology of Thiols, Alvarez, B. ; Comini, M. A. ; Salinas, G. ; Trujillo, M. , Eds.; Elsevier, 2022; pp. 177–196.

[183] Feng, J. ; Carreño, M. ; Jung, H. ; Naidu, S. D. ; Diaz, N. A. ; Ang, A. D. ; Kulkarni, B. ; Kisielewski, D. ; Suzuki, T. ; Yamamoto, M. The electrophilic metabolite of kynurenine, kynurenine-CKA, targets C151 in Keap1 to derepress Nrf2. bioRxiv 2025, 10.1101/2025.11.18.689077. PMC12828764 41534303

[184] Straus D. S., Pascual G., Li M., Welch J. S., Ricote M., Hsiang C. H., Sengchanthalangsy L. L., Ghosh G., Glass C. K. (2000). 15-deoxy-delta 12,14-prostaglandin
J2 inhibits multiple steps in the NF-kappa B signaling pathway. Proc. Natl. Acad. Sci. U. S. A..

[185] Cui T., Schopfer F. J., Zhang J., Chen K., Ichikawa T., Baker P. R., Batthyany C., Chacko B. K., Feng X., Patel R. P. (2006). Nitrated
fatty acids: Endogenous anti-inflammatory
signaling mediators. J. Biol. Chem..

[186] Garcia-Pineres A. J., Castro V., Mora G., Schmidt T. J., Strunck E., Pahl H. L., Merfort I. (2001). Cysteine 38
in p65/NF-kappaB
plays a crucial role in DNA binding inhibition by sesquiterpene lactones. J. Biol. Chem..

[187] Crozier-Reabe K. R., Phillips R. S., Moran G. R. (2008). Kynurenine 3-monooxygenase
from Pseudomonas fluorescens: substrate-like inhibitors both stimulate
flavin reduction and stabilize the flavin-peroxo intermediate yet
result in the production of hydrogen peroxide. Biochemistry.

[188] Phillips R. S., Iradukunda E. C., Hughes T., Bowen J. P. (2019). Modulation
of Enzyme Activity in the Kynurenine Pathway by Kynurenine Monooxygenase
Inhibition. Front. Mol. Biosci..

[189] Garrison A. M., Parrott J. M., Tunon A., Delgado J., Redus L., O’Connor J. C. (2018). Kynurenine pathway metabolic balance
influences microglia activity: Targeting kynurenine monooxygenase
to dampen neuroinflammation. Psychoneuroendocrinology.

[190] Xue C., Li G., Zheng Q., Gu X., Shi Q., Su Y., Chu Q., Yuan X., Bao Z., Lu J., Li L. (2023). Tryptophan metabolism in health and
disease. Cell Metab..

[191] Goldstein L. E., Leopold M. C., Huang X., Atwood C. S., Saunders A. J., Hartshorn M., Lim J. T., Faget K. Y., Muffat J. A., Scarpa R. C. (2000). 3-Hydroxykynurenine
and 3-hydroxyanthranilic acid generate hydrogen peroxide and promote
alpha-Crystallin cross-linking by metal ion reduction. Biochemistry.

[192] Winterbourn C. C. (2020). Biological
chemistry of superoxide radicals. ChemTexts.

[193] Colin-Gonzalez A. L., Maldonado P. D., Santamaria A. (2013). 3-Hydroxykynurenine:
an intriguing molecule exerting dual actions in the central nervous
system. Neurotoxicology.

[194] Aquilina J. A., Carver J. A., Truscott R. J. (1999). Elucidation
of a
novel polypeptide cross-link involving 3-hydroxykynurenine. Biochemistry.

[195] Bova L. M., Wood A. M., Jamie J. F., Truscott R. J. (1999). UV filter
compounds in human lenses: the origin of 4-(2-amino-3-hydroxyphenyl)-4-oxobutanoic
acid O-beta-D-glucoside. Invest Ophthalmol Vis
Sci..

[196] Vazquez S., Garner B., Sheil M. M., Truscott R. J. (2000). Characterisation
of the major autoxidation products of 3-hydroxykynurenine under physiological
conditions. Free Radic Res..

[197] Eastman C. L., Guilarte T. R. (1989). Cytotoxicity of
3-hydroxykynurenine
in a neuronal hybrid cell line. Brain Res..

[198] Okuda S., Nishiyama N., Saito H., Katsuki H. (1996). Hydrogen peroxide-mediated
neuronal cell death induced by an endogenous neurotoxin, 3-hydroxykynurenine. Proc. Natl. Acad. Sci. U. S. A..

[199] Okuda S., Nishiyama N., Saito H., Katsuki H. (1998). 3-Hydroxykynurenine,
an endogenous oxidative stress generator, causes neuronal cell death
with apoptotic features and region selectivity. J. Neurochem..

[200] Reyes-Ocampo J., Ramirez-Ortega D., Cervantes G. I., Pineda B., Balderas P. M., Gonzalez-Esquivel D., Sanchez-Chapul L., Lugo-Huitron R., Silva-Adaya D., Rios C. (2015). Mitochondrial dysfunction related to cell damage induced
by 3-hydroxykynurenine and 3-hydroxyanthranilic acid: Non-dependent-effect
of early reactive oxygen species production. Neurotoxicology.

[201] Christen S., Peterhans E., Stocker R. (1990). Antioxidant activities
of some tryptophan metabolites: possible implication for inflammatory
diseases. Proc. Natl. Acad. Sci. U. S. A..

[202] Leipnitz G., Schumacher C., Dalcin K. B., Scussiato K., Solano A., Funchal C., Dutra-Filho C. S., Wyse A. T., Wannmacher C. M., Latini A., Wajner M. (2007). In vitro evidence
for an antioxidant role of 3-hydroxykynurenine and 3-hydroxyanthranilic
acid in the brain. Neurochem. Int..

[203] Goda K., Hamane Y., Kishimoto R., Ogishi Y. (1999). Radical scavenging properties of tryptophan metabolites.
Estimation of their radical reactivity. Adv.
Exp. Med. Biol..

[204] Goshima N., Wadano A., Miura K. (1986). 3-Hydroxykynurenine
as O2-. scavenger in the blowfly, Aldrichina grahami. Biochem. Biophys. Res. Commun..

[205] Pearson S. J., Reynolds G. P. (1992). Increased brain
concentrations of
a neurotoxin, 3-hydroxykynurenine, in Huntington’s disease. Neurosci. Lett..

[206] Duleu S., Mangas A., Sevin F., Veyret B., Bessede A., Geffard M. (2010). Circulating Antibodies to IDO/THO
Pathway Metabolites in Alzheimer’s Disease. Int. J. Alzheimers. Dis..

[207] Schwarz M. J., Guillemin G. J., Teipel S. J., Buerger K., Hampel H. (2013). Increased 3-hydroxykynurenine
serum concentrations
differentiate Alzheimer’s disease patients from controls. Eur. Arch. Psychiatry Clin. Neurosci..

[208] Lewitt P. A., Li J., Lu M., Beach T. G., Adler C. H., Guo L., The Arizona
Parkinson’s
Disease Consortium (2013). 3-hydroxykynurenine
and other Parkinson’s disease biomarkers discovered by metabolomic
analysis. Mov. Disord..

[209] Guidetti P., Bates G. P., Graham R. K., Hayden M. R., Leavitt B. R., MacDonald M. E., Slow E. J., Wheeler V. C., Woodman B., Schwarcz R. (2006). Elevated brain 3-hydroxykynurenine
and quinolinate levels in Huntington disease mice. Neurobiol. Dis..

[210] Guidetti P., Reddy P. H., Tagle D. A., Schwarcz R. (2000). Early kynurenergic
impairment in Huntington’s disease and in a transgenic animal
model. Neurosci. Lett..

[211] Sathyasaikumar K. V., Stachowski E. K., Amori L., Guidetti P., Muchowski P. J., Schwarcz R. (2010). Dysfunctional kynurenine pathway
metabolism in the R6/2 mouse model of Huntington’s disease. J. Neurochem..

[212] Bakker L., Kohler S., Eussen S., Choe K., van den Hove D. L. A., Kenis G., Rutten B. P. F., Ulvik A., Ueland P. M., Verhey F. R. J., Ramakers I. (2023). Correlations
between
kynurenines in plasma and CSF, and their relation to markers of Alzheimer’s
disease pathology. Brain. Behav. Immun..

[213] Mole D. J., McFerran N. V., Collett G., O’Neill C., Diamond T., Garden O. J., Kylanpaa L., Repo H., Deitch E. A. (2008). Tryptophan catabolites in mesenteric
lymph may contribute
to pancreatitis-associated organ failure. Br.
J. Surg..

[214] Skouras C., Zheng X., Binnie M., Homer N. Z., Murray T. B., Robertson D., Briody L., Paterson F., Spence H., Derr L. (2016). Increased levels of
3-hydroxykynurenine parallel disease severity in human acute pancreatitis. Sci. Rep..

[215] Mole D. J., Webster S. P., Uings I., Zheng X., Binnie M., Wilson K., Hutchinson J. P., Mirguet O., Walker A., Beaufils B., Ancellin N., Trottet L., Beneton V., Mowat C. G., Wilkinson M., Rowland P., Haslam C., McBride A., Homer N. Z., Baily J. E., Sharp M. G., Garden O. J., Hughes J., Howie S. E., Holmes D. S., Liddle J., Iredale J. P. (2016). Kynurenine-3-monooxygenase
inhibition prevents multiple organ failure in rodent models of acute
pancreatitis. Nat. Med..

[216] Maitre M., Taleb O., Jeltsch-David H., Klein C., Mensah-Nyagan A. G. (2024). Xanthurenic acid: A role in brain
intercellular signaling. J. Neurochem..

[217] Sathyasaikumar K. V., Tararina M., Wu H. Q., Neale S. A., Weisz F., Salt T. E., Schwarcz R. (2017). Xanthurenic
Acid Formation
from 3-Hydroxykynurenine in the Mammalian Brain: Neurochemical Characterization
and Physiological Effects. Neuroscience.

[218] Bartlett R. D., Esslinger C. S., Thompson C. M., Bridges R. J. (1998). Substituted
quinolines as inhibitors of L-glutamate transport into synaptic vesicles. Neuropharmacology.

[219] Neale S. A., Copeland C. S., Uebele V. N., Thomson F. J., Salt T. E. (2013). Modulation of hippocampal synaptic transmission by
the kynurenine pathway member xanthurenic acid and other VGLUT inhibitors. Neuropsychopharmacology.

[220] Copeland C. S., Neale S. A., Salt T. E. (2013). Actions of Xanthurenic
acid, a putative endogenous Group II metabotropic glutamate receptor
agonist, on sensory transmission in the thalamus. Neuropharmacology.

[221] Fazio F., Lionetto L., Curto M., Iacovelli L., Cavallari M., Zappulla C., Ulivieri M., Napoletano F., Capi M., Corigliano V. (2015). Xanthurenic Acid Activates
mGlu2/3 Metabotropic Glutamate Receptors and is a Potential Trait
Marker for Schizophrenia. Sci. Rep..

[222] Taleb O., Maammar M., Klein C., Maitre M., Mensah-Nyagan A. G. (2021). A Role
for Xanthurenic Acid in the Control of Brain
Dopaminergic Activity. Int. J. Mol. Sci..

[223] Xue C., Gu X., Zheng Q., Shi Q., Yuan X., Chu Q., Jia J., Su Y., Bao Z., Lu J. (2023). Effects of 3-HAA on HCC by Regulating the Heterogeneous
Macrophages-A
scRNA-Seq Analysis. Adv. Sci..

[224] Krause D., Suh H. S., Tarassishin L., Cui Q. L., Durafourt B. A., Choi N., Bauman A., Cosenza-Nashat M., Antel J. P., Zhao M. L., Lee S. C. (2011). The tryptophan
metabolite 3-hydroxyanthranilic acid plays anti-inflammatory and neuroprotective
roles during inflammation: role of hemeoxygenase-1. Am. J. Pathol..

[225] Tan Q., Deng S., Xiong L. (2025). Role of Kynurenine
and Its Derivatives
in Liver Diseases: Recent Advances and Future Clinical Perspectives. Int. J. Mol. Sci..

[226] Pawlak K., Kowalewska A., Mysliwiec M., Pawlak D. (2010). 3-hydroxyanthranilic acid is independently
associated
with monocyte chemoattractant protein-1 (CCL2) and macrophage inflammatory
protein-1beta (CCL4) in patients with chronic kidney disease. Clin. Biochem..

[227] Baran H., Schwarcz R. (1991). Evidence for the preferential
production
of 3-hydroxyanthranilic acid from anthranilic acid in the rat brain. Adv. Exp. Med. Biol..

[228] Darlington L. G., Forrest C. M., Mackay G. M., Smith R. A., Smith A. J., Stoy N., Stone T. W. (2010). On the
Biological
Importance of the 3-hydroxyanthranilic Acid: Anthranilic Acid Ratio. Int. J. Tryptophan Res..

[229] Thomas S. R., Witting P. K., Stocker R. (1996). 3-Hydroxyanthranilic
acid is an efficient, cell-derived co-antioxidant for alpha-tocopherol,
inhibiting human low density lipoprotein and plasma lipid peroxidation. J. Biol. Chem..

[230] Christen S., Southwell-Keely P. T., Stocker R. (1992). Oxidation of 3-hydroxyanthranilic
acid to the phenoxazinone cinnabarinic acid by peroxyl radicals and
by compound I of peroxidases or catalase. Biochemistry.

[231] Chobot V., Hadacek F., Weckwerth W., Kubicova L. (2015). Iron chelation and redox chemistry of anthranilic acid
and 3-hydroxyanthranilic acid: A comparison of two structurally related
kynurenine pathway metabolites to obtain improved insights into their
potential role in neurological disease development. J. Organomet. Chem..

[232] Dang H., Castro-Portuguez R., Espejo L., Backer G., Freitas S., Spence E., Meyers J., Shuck K., Gardea E. A., Chang L. M., Balsa J., Thorns N., Corban C., Liu T., Bean S., Sheehan S., Korstanje R., Sutphin G. L. (2023). On the benefits of the tryptophan
metabolite 3-hydroxyanthranilic acid in Caenorhabditis elegans and
mouse aging. Nat. Commun..

[233] Benavente M. G., Truscott R. J. (1991). Modification of proteins by 3-hydroxyanthranilic
acid: the role of lysine residues. Arch. Biochem.
Biophys..

[234] Ruan Q., Peng Y., Yi X., Yang J., Ai Q., Liu X., He Y., Shi Y. (2025). The tryptophan metabolite
3-hydroxyanthranilic acid alleviates hyperoxia-induced bronchopulmonary
dysplasia via inhibiting ferroptosis. Redox
Biol..

[235] Lee W. S., Lee S. M., Kim M. K., Park S. G., Choi I. W., Choi I., Joo Y. D., Park S. J., Kang S. W., Seo S. K. (2013). The tryptophan metabolite
3-hydroxyanthranilic
acid suppresses T cell responses by inhibiting dendritic cell activation. Int. Immunopharmacol..

[236] Hayashi T., Mo J. H., Gong X., Rossetto C., Jang A., Beck L., Elliott G. I., Kufareva I., Abagyan R., Broide D. H., Lee J., Raz E. (2007). 3-Hydroxyanthranilic
acid inhibits PDK1 activation and suppresses experimental asthma by
inducing T cell apoptosis. Proc. Natl. Acad.
Sci. U. S. A..

[237] Bago A., Cayuela M. L., Gil A., Calvo E., Vazquez J., Queiro A., Schopfer F. J., Radi R., Serrador J. M., Iniguez M. A. (2023). Nitro-oleic acid regulates T cell
activation through post-translational modification of calcineurin. Proc. Natl. Acad. Sci. U. S. A..

[238] Muri J., Wolleb H., Broz P., Carreira E. M., Kopf M. (2020). Electrophilic Nrf2 activators and itaconate inhibit inflammation
at low dose and promote IL-1beta production and inflammatory apoptosis
at high dose. Redox Biol..

[239] Peng H., Guerau-de-Arellano M., Mehta V. B., Yang Y., Huss D. J., Papenfuss T. L., Lovett-Racke A. E., Racke M. K. (2012). Dimethyl fumarate inhibits dendritic cell maturation
via nuclear factor kappaB (NF-kappaB) and extracellular signal-regulated
kinase 1 and 2 (ERK1/2) and mitogen stress-activated kinase 1 (MSK1)
signaling. J. Biol. Chem..

[240] Quagliariello E., Papa S., Saccone C., Alifano A. (1964). Effect of
3-hydroxyanthranilic acid on the mitochondrial respiratory system. Biochem. J..

[241] Gawel K. (2024). A Review on
the Role and Function of Cinnabarinic Acid, a “Forgotten”
Metabolite of the Kynurenine Pathway. Cells.

[242] Fazio F., Lionetto L., Molinaro G., Bertrand H. O., Acher F., Ngomba R. T., Notartomaso S., Curini M., Rosati O., Scarselli P., Di Marco R., Battaglia G., Bruno V., Simmaco M., Pin J. P., Nicoletti F., Goudet C. (2012). Cinnabarinic acid,
an endogenous metabolite of the kynurenine pathway, activates type
4 metabotropic glutamate receptors. Mol. Pharmacol..

[243] Patil N. Y., Rus I., Downing E., Mandala A., Friedman J. E., Joshi A. D. (2022). Cinnabarinic Acid
Provides Hepatoprotection
Against Nonalcoholic Fatty Liver Disease. J.
Pharmacol. Exp. Ther..

[244] Lowe M. M., Mold J. E., Kanwar B., Huang Y., Louie A., Pollastri M. P., Wang C., Patel G., Franks D. G., Schlezinger J., Sherr D. H., Silverstone A. E., Hahn M. E., McCune J. M. (2014). Identification of cinnabarinic acid
as a novel endogenous aryl hydrocarbon receptor ligand that drives
IL-22 production. PLoS One.

[245] Pasceri R., Siegel D., Ross D., Moody C. J. (2013). Aminophenoxazinones
as inhibitors of indoleamine 2,3-dioxygenase (IDO). Synthesis of exfoliazone
and chandrananimycin A. J. Med. Chem..

[246] Carr G., Tay W., Bottriell H., Andersen S. K., Mauk A. G., Andersen R. J. (2009). Plectosphaeroic
acids A, B, and C, indoleamine 2,3-dioxygenase inhibitors produced
in culture by a marine isolate of the fungus Plectosphaerella cucumerina. Org. Lett..

[247] Joshi A. D., Carter D. E., Harper T. A., Elferink C. J. (2015). Aryl hydrocarbon
receptor-dependent stanniocalcin 2 induction by cinnabarinic acid
provides cytoprotection against endoplasmic reticulum and oxidative
stress. J. Pharmacol. Exp. Ther..

[248] Harper T. A., Joshi A. D., Elferink C. J. (2013). Identification
of stanniocalcin 2 as a novel aryl hydrocarbon receptor target gene. J. Pharmacol. Exp. Ther..

[249] Qie S., Sang N. (2022). Stanniocalcin 2 (STC2):
a universal tumour biomarker
and a potential therapeutical target. J. Exp.
Clin. Cancer Res..

[250] Hiramatsu R., Hara T., Akimoto H., Takikawa O., Kawabe T., Isobe K., Nagase F. (2008). Cinnabarinic
acid generated
from 3-hydroxyanthranilic acid strongly induces apoptosis in thymocytes
through the generation of reactive oxygen species and the induction
of caspase. J. Cell. Biochem..

[251] Wang Y., Liu K. F., Yang Y., Davis I., Liu A. (2020). Observing 3-hydroxyanthranilate-3,4-dioxygenase
in action through
a crystalline lens. Proc. Natl. Acad. Sci. U.
S. A..

[252] Li T., Ma J. K., Hosler J. P., Davidson V. L., Liu A. (2007). Detection
of transient intermediates in the metal-dependent nonoxidative decarboxylation
catalyzed by alpha-amino-beta-carboxymuconate-epsilon-semialdehyde
decarboxylase. J. Am. Chem. Soc..

[253] Pucci L., Perozzi S., Cimadamore F., Orsomando G., Raffaelli N. (2007). Tissue expression and biochemical
characterization of human 2-amino 3-carboxymuconate 6-semialdehyde
decarboxylase, a key enzyme in tryptophan catabolism. FEBS J..

[254] Huo L., Liu F., Iwaki H., Li T., Hasegawa Y., Liu A. (2015). Human alpha-amino-beta-carboxymuconate-epsilon-semialdehyde
decarboxylase
(ACMSD): a structural and mechanistic unveiling. Proteins.

[255] Davis I., Yang Y., Wherritt D., Liu A. (2018). Reassignment
of the human aldehyde dehydrogenase ALDH8A1 (ALDH12) to the kynurenine
pathway in tryptophan catabolism. J. Biol. Chem..

[256] Sabari B. R., Tang Z., Huang H., Yong-Gonzalez V., Molina H., Kong H. E., Dai L., Shimada M., Cross J. R., Zhao Y., Roeder R. G., Allis C. D. (2015). Intracellular
crotonyl-CoA stimulates transcription through p300-catalyzed histone
crotonylation. Mol. Cell.

[257] Tan M., Luo H., Lee S., Jin F., Yang J. S., Montellier E., Buchou T., Cheng Z., Rousseaux S., Rajagopal N., Lu Z., Ye Z., Zhu Q., Wysocka J., Ye Y., Khochbin S., Ren B., Zhao Y. (2011). Identification of 67 histone marks and histone lysine crotonylation
as a new type of histone modification. Cell.

[258] Martynowski D., Eyobo Y., Li T., Yang K., Liu A., Zhang H. (2006). Crystal structure of
alpha-amino-beta-carboxymuconate-epsilon-semialdehyde
decarboxylase: insight into the active site and catalytic mechanism
of a novel decarboxylation reaction. Biochemistry.

[259] Li T., Iwaki H., Fu R., Hasegawa Y., Zhang H., Liu A. (2006). Alpha-amino-beta-carboxymuconic-epsilon-semialdehyde
decarboxylase
(ACMSD) is a new member of the amidohydrolase superfamily. Biochemistry.

[260] Yang Y., Borel T., de Azambuja F., Johnson D., Sorrentino J. P., Udokwu C., Davis I., Liu A., Altman R. A. (2021). Diflunisal
Derivatives as Modulators of ACMS Decarboxylase
Targeting the Tryptophan-Kynurenine Pathway. J. Med. Chem..

[261] Statter M., Krieger I. (1983). Picolinic carboxylase activity in
rat liver and kidney. I. Influence of growth, sex, gestation, lactation,
and nutritional imbalance. J. Pediatr. Gastroenterol.
Nutr..

[262] Grant R. S., Coggan S. E., Smythe G. A. (2009). The physiological
action of picolinic Acid in the human brain. Int. J. Tryptophan Res..

[263] Prodinger J., Loacker L. J., Schmidt R. L., Ratzinger F., Greiner G., Witzeneder N., Hoermann G., Jutz S., Pickl W. F., Steinberger P., Marculescu R., Schmetterer K. G. (2016). The tryptophan metabolite picolinic
acid suppresses
proliferation and metabolic activity of CD4+ T cells and inhibits
c-Myc activation. J. Leukoc. Biol..

[264] Coggan S. E., Smythe G. A., Bilgin A., Grant R. S. (2009). Age and
circadian influences on picolinic acid concentrations in human cerebrospinal
fluid. J. Neurochem..

[265] Zuwala-Jagiello J., Pazgan-Simon M., Simon K., Warwas M. (2012). Picolinic
acid in patients with chronic hepatitis C infection: a preliminary
report. Mediators Inflamm..

[266] Gargas M. L., Norton R. L., Paustenbach D. J., Finley B. L. (1994). Urinary excretion
of chromium by humans following ingestion
of chromium picolinate. Implications for biomonitoring. Drug Metab. Dispos..

[267] Seal C. J., Heaton F. W. (1985). Effect of dietary picolinic acid
on the metabolism of exogenous and endogenous zinc in the rat. J. Nutr..

[268] Wang X., Davis I., Liu A., Miller A., Shamsi S. A. (2013). Improved separation and detection of picolinic acid
and quinolinic acid by capillary electrophoresis-mass spectrometry:
application to analysis of human cerebrospinal fluid. J. Chromatogr. A..

[269] Suzuki K., Yasuda M., Yamasaki K. (1957). Stability Constants
of Picolinic and Quinaldic Acid Chelates of Bivalent Metals. J. Phys. Chem..

[270] Testa U., Louache F., Titeux M., Thomopoulos P., Rochant H. (1985). The iron-chelating agent picolinic acid enhances transferrin
receptors expression in human erythroleukaemic cell lines. Br. J. Hamaetol..

[271] Jhamandas K. H., Boegman R. J., Beninger R. J., Flesher S. (1998). Role of zinc
in blockade of excitotoxic action of quinolinic acid by picolinic
acid. Amino Acids..

[272] Kalisch B. E., Jhamandas K., Boegman R. J., Beninger R. J. (1994). Picolinic
acid protects against quinolinic acid-induced depletion of NADPH diaphorase
containing neurons in the rat striatum. Brain
Res..

[273] Melillo G., Cox G. W., Biragyn A., Sheffler L. A., Varesio L. (1994). Regulation
of nitric-oxide synthase mRNA expression
by interferon-gamma and picolinic acid. J. Biol.
Chem..

[274] Varesio L., Clayton M., Blasi E., Ruffman R., Radzioch D. (1990). Picolinic
acid, a catabolite of tryptophan, as the
second signal in the activation of IFN-gamma-primed macrophages. J. Immunol..

[275] Bosco M. C., Rapisarda A., Massazza S., Melillo G., Young H., Varesio L. (2000). The tryptophan catabolite picolinic
acid selectively induces the chemokines macrophage inflammatory protein-1
alpha and −1 beta in macrophages. J.
Immunol..

[276] Fernandez-Pol J. A., Johnson G. S. (1977). Selective toxicity induced by picolinic
acid in simian virus 40-transformed cells in tissue culture. Cancer Res..

[277] Fernandez-Pol J. A., Klos D. J., Hamilton P. D. (2001). Antiviral, cytotoxic
and apoptotic activities of picolinic acid on human immunodeficiency
virus-1 and human herpes simplex virus-2 infected cells. Anticancer Res..

[278] Savita V., Pal V. S., Bagul K., Mudgal V. (2023). A Comparative
Study of Picolinic Acid Levels in Patients of Severe Depression with
and without Suicidality. Arch. Med. Health Sci..

[279] Fukuoka S., Ishiguro K., Yanagihara K., Tanabe A., Egashira Y., Sanada H., Shibata K. (2002). Identification
and expression of a cDNA encoding human alpha-amino-beta-carboxymuconate-epsilon-semialdehyde
decarboxylase (ACMSD). A key enzyme for the tryptophan-niacine pathway
and “quinolinate hypothesis. J. Biol.
Chem..

[280] Guillemin G. J., Kerr S. J., Smythe G. A., Smith D. G., Kapoor V., Armati P. J., Croitoru J., Brew B. J. (2001). Kynurenine
pathway metabolism in human astrocytes: a paradox for neuronal protection. J. Neurochem..

[281] Heyes M. P., Achim C. L., Wiley C. A., Major E. O., Saito K., Markey S. P. (1996). Human microglia
convert l-tryptophan
into the neurotoxin quinolinic acid. Biochem.
J..

[282] Lahdou I., Sadeghi M., Oweira H., Fusch G., Daniel V., Mehrabi A., Jung G., Elhadedy H., Schmidt J., Sandra-Petrescu F., Iancu M., Opelz G., Terness P., Schefold J. C. (2013). Increased
serum levels of quinolinic
acid indicate enhanced severity of hepatic dysfunction in patients
with liver cirrhosis. Hum. Immunol..

[283] de Carvalho L. P., Bochet P., Rossier J. (1996). The endogenous
agonist
quinolinic acid and the non endogenous homoquinolinic acid discriminate
between NMDAR2 receptor subunits. Neurochem.
Int..

[284] Perkins M. N., Stone T. W. (1983). Pharmacology and
regional variations
of quinolinic acid-evoked excitations in the rat central nervous system. J. Pharmacol. Exp. Ther..

[285] Schwarcz R., Kohler C. (1983). Differential vulnerability
of central
neurons of the rat to quinolinic acid. Neurosci.
Lett..

[286] Lugo-Huitron R., Ugalde Muniz P., Pineda B., Pedraza-Chaverri J., Rios C., Perez-de la Cruz V. (2013). Quinolinic acid: an endogenous neurotoxin
with multiple targets. Oxid. Med. Cell. Longev..

[287] Tavares R. G., Tasca C. I., Santos C. E., Wajner M., Souza D. O., Dutra-Filho C. S. (2000). Quinolinic acid inhibits glutamate
uptake into synaptic vesicles from rat brain. Neuroreport.

[288] Tavares R. G., Tasca C. I., Santos C. E., Alves L. B., Porciuncula L. O., Emanuelli T., Souza D. O. (2002). Quinolinic acid
stimulates synaptosomal glutamate release and inhibits glutamate uptake
into astrocytes. Neurochem. Int..

[289] Obrenovitch T. P. (2001). Quinolinic acid accumulation during
neuroinflammation.
Does it imply excitotoxicity?. Ann. N.Y. Acad.
Sci..

[290] Patneau D. K., Mayer M. L. (1990). Structure-activity relationships
for amino acid transmitter candidates acting at N-methyl-D-aspartate
and quisqualate receptors. J. Neurosci..

[291] Stone T. W. (2001). Kynurenines in the CNS: from endogenous
obscurity to
therapeutic importance. Prog. Neurobiol..

[292] Goda K., Kishimoto R., Shimizu S., Hamane Y., Ueda M. (1996). Quinolinic acid and
active oxygens. Possible contribution of active
Oxygens during cell death in the brain. Adv.
Exp. Med. Biol..

[293] Iwahashi H., Kawamori H., Fukushima K. (1999). Quinolinic
acid, alpha-picolinic acid, fusaric acid, and 2,6-pyridinedicarboxylic
acid enhance the Fenton reaction in phosphate buffer. Chem. Biol. Interact..

[294] Rios C., Santamaria A. (1991). Quinolinic acid is a potent lipid
peroxidant in rat brain homogenates. Neurochem.
Res..

[295] Stipek S., Stastny F., Platenik J., Crkovska J., Zima T. (1997). The effect
of quinolinate on rat brain lipid peroxidation is dependent
on iron. Neurochem. Int..

[296] Braidy N., Grant R., Adams S., Guillemin G. J. (2010). Neuroprotective
effects of naturally occurring polyphenols on quinolinic acid-induced
excitotoxicity in human neurons. FEBS J..

[297] Block F., Schwarz M. (1994). Expression of GFAP
in the striatum
and its projection areas in response to striatal quinolinic acid lesion
in rats. Neuroreport.

[298] Schiefer J., Topper R., Schmidt W., Block F., Heinrich P. C., Noth J., Schwarz M. (1998). Expression
of interleukin
6 in the rat striatum following stereotaxic injection of quinolinic
acid. J. Neuroimmunol..

[299] Macaya A., Munell F., Gubits R. M., Burke R. E. (1994). Apoptosis
in substantia nigra following developmental striatal excitotoxic injury. Proc. Natl. Acad. Sci. U. S. A..

[300] Jeon B. S., Kholodilov N. G., Oo T. F., Kim S. Y., Tomaselli K. J., Srinivasan A., Stefanis L., Burke R. E. (1999). Activation
of caspase-3 in developmental models of programmed cell death in neurons
of the substantia nigra. J. Neurochem..

[301] Guillemin G. J., Wang L., Brew B. J. (2005). Quinolinic
acid
selectively induces apoptosis of human astrocytes: potential role
in AIDS dementia complex. J. Neuroinflammation..

[302] Qin Z., Wang Y., Chasea T. N. (2000). A caspase-3-like
protease is involved
in NF-kappaB activation induced by stimulation of N-methyl-D-aspartate
receptors in rat striatum. Brain Res. Mol. Brain
Res..

[303] Wang Y., Dong X. X., Cao Y., Liang Z. Q., Han R., Wu J. C., Gu Z. L., Qin Z. H. (2009). induction contributes
to excitotoxic neuronal death in rat striatum through apoptotic and
autophagic mechanisms. Eur. J. Neurosci..

[304] Schwarcz R., Whetsell W. O., Mangano R. M. (1983). Quinolinic
acid:
an endogenous metabolite that produces axon-sparing lesions in rat
brain. Science.

[305] Reynolds D. S., Morton A. J. (1998). Changes in blood-brain
barrier permeability
following neurotoxic lesions of rat brain can be visualised with trypan
blue. J. Neurosci. Methods..

[306] Guillemin G. J., Croitoru-Lamoury J., Dormont D., Armati P. J., Brew B. J. (2003). Quinolinic acid
upregulates chemokine production and
chemokine receptor expression in astrocytes. Glia.

[307] Katsyuba E., Mottis A., Zietak M., De Franco F., van der Velpen V., Gariani K., Ryu D., Cialabrini L., Matilainen O., Liscio P., Giacche N., Stokar-Regenscheit N., Legouis D., de Seigneux S., Ivanisevic J., Raffaelli N., Schoonjans K., Pellicciari R., Auwerx J. (2018). De novo NAD­(+) synthesis enhances mitochondrial function
and improves health. Nature.

[308] Marletta A. S., Massarotti A., Orsomando G., Magni G., Rizzi M., Garavaglia S. (2015). Crystal structure
of human nicotinic acid phosphoribosyltransferase. FEBS Open Bio..

[309] Abdellatif M., Sedej S., Kroemer G. (2021). NAD­(+) Metabolism in
Cardiac Health, Aging, and Disease. Circulation.

[310] Xie N., Zhang L., Gao W., Huang C., Huber P. E., Zhou X., Li C., Shen G., Zou B. (2020). NAD­(+) metabolism:
pathophysiologic mechanisms and therapeutic potential. Signal Transduction Targeted Ther..

[311] Hestad K., Alexander J., Rootwelt H., Aaseth J. O. (2022). The Role
of Tryptophan Dysmetabolism and Quinolinic Acid in Depressive and
Neurodegenerative Diseases. Biomolecules.

[312] Sahm F., Oezen I., Opitz C. A., Radlwimmer B., von Deimling A., Ahrendt T., Adams S., Bode H. B., Guillemin G. J., Wick W., Platten M. (2013). The endogenous tryptophan
metabolite and NAD+ precursor quinolinic acid confers resistance of
gliomas to oxidative stress. Cancer Res..

[313] Heyes M. P., Brew B. J., Saito K., Quearry B. J., Price R. W., Lee K., Bhalla R. B., Der M., Markey S. P. (1992). Inter-relationships between quinolinic acid, neuroactive
kynurenines, neopterin and beta 2-microglobulin in cerebrospinal fluid
and serum of HIV-1-infected patients. J. Neuroimmunol..

[314] Cathomas F., Guetter K., Seifritz E., Klaus F., Kaiser S. (2021). Quinolinic acid is associated with
cognitive deficits
in schizophrenia but not major depressive disorder. Sci. Rep..

[315] Editors P. O. (2024). Expression of Concern: The Excitotoxin
Quinolinic Acid
Induces Tau Phosphorylation in Human Neurons. PLoS One.

[316] Rahman A., Ting K., Cullen K. M., Braidy N., Brew B. J., Guillemin G. J. (2009). The excitotoxin
quinolinic acid induces
tau phosphorylation in human neurons. PLoS One.

[317] Ohashi H., Saito K., Fujii H., Wada H., Furuta N., Takemura M., Maeda S., Seishima M. (2004). Changes in
quinolinic acid production and its related enzymes following D-galactosamine
and lipopolysaccharide-induced hepatic injury. Arch. Biochem. Biophys..

[318] Wang B., Zhen D., Wei J., Ding L., Ding W., Portha B., Liu J. (2025). Quinolinic acid protects
mouse liver from high-fat diet induced MASLD by inhibiting lipid uptake
gene expression. Eur. J. Pharmacol..

[319] Thaker A. I., Rao M. S., Bishnupuri K. S., Kerr T. A., Foster L., Marinshaw J. M., Newberry R. D., Stenson W. F., Ciorba M. A. (2013). IDO1 metabolites
activate beta-catenin signaling to promote cancer cell proliferation
and colon tumorigenesis in mice. Gastroenterology.

[320] Nakamura K., Sugawara Y., Sawabe K., Ohashi A., Tsurui H., Xiu Y., Ohtsuji M., Lin Q. S., Nishimura H., Hasegawa H., Hirose S. (2006). Late developmental
stage-specific role of tryptophan hydroxylase 1 in brain serotonin
levels. J. Neurosci..

[321] Zill P., Buttner A., Eisenmenger W., M?ller H. J., Ackenheil M., Bondy B. (2007). Analysis of tryptophan
hydroxylase I and II mRNA expression in the human brain: a post-mortem
study. J. Psychiatr. Res..

[322] Lim J. E., Pinsonneault J., Sadee W., Saffen D. (2007). Tryptophan
hydroxylase 2 (TPH2) haplotypes predict levels of TPH2 mRNA expression
in human pons. Mol. Psychiatry.

[323] Liu N., Sun S., Wang P., Sun Y., Hu Q., Wang X. (2021). The Mechanism of Secretion and Metabolism
of Gut-Derived 5-Hydroxytryptamine. Int J Mol
Sci..

[324] Mercado C. P., Kilic F. (2010). Molecular mechanisms
of SERT in platelets:
regulation of plasma serotonin levels. Mol.
Interv..

[325] Kuhn D. M., Sakowski S. A., Geddes T. J., Wilkerson C., Haycock J. W. (2007). Phosphorylation and activation of
tryptophan hydroxylase
2: identification of serine-19 as the substrate site for calcium,
calmodulin-dependent protein kinase II. J. Neurochem..

[326] Lehmann I. T., Bobrovskaya L., Gordon S. L., Dunkley P. R., Dickson P. W. (2006). Differential regulation
of the human tyrosine hydroxylase
isoforms via hierarchical phosphorylation. J.
Biol. Chem..

[327] Kalinichenko L. S., Kornhuber J., Sinning S., Haase J., Müller C. P. (2024). Serotonin
Signaling through Lipid Membranes. ACS Chem.
Neurosci..

[328] Li G., Dong S., Liu C., Yang J., Rensen P. C. N., Wang Y. (2024). Serotonin signaling
to regulate energy metabolism:
a gut microbiota perspective. Life Metabolism.

[329] Herr N., Bode C., Duerschmied D. (2017). The Effects
of Serotonin in Immune Cells. Front Cardiovasc
Med..

[330] Ferguson S. S., Caron M. G. (1998). G protein-coupled receptor adaptation
mechanisms. Semin Cell Dev Biol..

[331] Lopez-Gimenez J. F., Gonzalez-Maeso J. (2017). Hallucinogens
and Serotonin 5-HT­(2A)
Receptor-Mediated Signaling Pathways. Curr.
Top Behav. Neurosci..

[332] Polter A. M., Li X. (2010). 5-HT1A receptor-regulated signal
transduction pathways in brain. Cell. Signal..

[333] Masson J., Emerit M. B., Hamon M., Darmon M. (2012). Serotonergic
signaling: multiple effectors and pleiotropic effects. Wiley Interdiscip. Rev.: membr. Transp. Signal..

[334] Derkach V., Surprenant A., North R. A. (1989). 5-HT3 receptors
are membrane ion channels. Nature.

[335] Muma N. A., Mi Z. (2015). Serotonylation and
Transamidation
of Other Monoamines. ACS Chem. Neurosci..

[336] Dai Y., Dudek N. L., Patel T. B., Muma N. A. (2008). Transglutaminase-catalyzed
transamidation: a novel mechanism for Rac1 activation by 5-hydroxytryptamine2A
receptor stimulation. J. Pharmacol. Exp. Ther..

[337] Walther D. J., Peter J. U., Winter S., Holtje M., Paulmann N., Grohmann M., Vowinckel J., Alamo-Bethencourt V., Wilhelm C. S., Ahnert-Hilger G., Bader M. (2003). Serotonylation of small GTPases is a signal transduction pathway
that triggers platelet alpha-granule release. Cell.

[338] Manzella C., Singhal M., Alrefai W. A., Saksena S., Dudeja P. K., Gill R. K. (2018). Serotonin is an endogenous regulator
of intestinal CYP1A1 via AhR. Sci. Rep..

[339] Ohara-Imaizumi M., Kim H., Yoshida M., Fujiwara T., Aoyagi K., Toyofuku Y., Nakamichi Y., Nishiwaki C., Okamura T., Uchida T., Fujitani Y., Akagawa K., Kakei M., Watada H., German M. S., Nagamatsu S. (2013). Serotonin regulates glucose-stimulated insulin secretion
from pancreatic beta cells during pregnancy. Proc. Natl. Acad. Sci. U. S. A..

[340] Kim K., Oh C. M., Ohara-Imaizumi M., Park S., Namkung J., Yadav V. K., Tamarina N. A., Roe M. W., Philipson L. H., Karsenty G., Nagamatsu S., German M. S., Kim H. (2015). Functional
role of serotonin in insulin secretion in a diet-induced insulin-resistant
state. Endocrinology.

[341] Almaca J., Molina J., Menegaz D., Pronin A. N., Tamayo A., Slepak V., Berggren P. O., Caicedo A. (2016). Human Beta
Cells Produce and Release Serotonin to Inhibit Glucagon Secretion
from Alpha Cells. Cell Rep..

[342] Paulmann N., Grohmann M., Voigt J. P., Bert B., Vowinckel J., Bader M., Skelin M., Jevsek M., Fink H., Rupnik M., Walther D. J. (2009). Intracellular
serotonin
modulates insulin secretion from pancreatic beta-cells by protein
serotonylation. PLoS Biol..

[343] Ohta Y., Kosaka Y., Kishimoto N., Wang J., Smith S. B., Honig G., Kim H., Gasa R. M., Neubauer N., Liou A., Tecott L. H., Deneris E. S., German M. S. (2011). Convergence of the insulin and serotonin
programs in the pancreatic beta-cell. Diabetes.

[344] Ono Y., Kataoka K. (2025). Glucocorticoids reduce
Slc2a2 (GLUT2) gene expression
through HNF1 in pancreatic beta-cells. J. Mol.
Endocrinol..

[345] Cai Y., Li X., Zhou H., Zhou J. (2022). The serotonergic system
dysfunction in diabetes mellitus. Front Cell
Neurosci..

[346] Oh C. M., Park S., Kim H. (2016). Serotonin
as a New
Therapeutic Target for Diabetes Mellitus and Obesity. Diabetes Metab. J..

[347] Guzel T., Mirowska-Guzel D. (2022). The Role of Serotonin Neurotransmission
in Gastrointestinal Tract and Pharmacotherapy. Molecules.

[348] Mawe G. M., Hoffman J. M. (2013). Serotonin signalling
in the gut–functions,
dysfunctions and therapeutic targets. Nat. Rev.
Gastroenterol. Hepatol..

[349] Watanabe H., Akasaka D., Ogasawara H., Sato K., Miyake M., Saito K., Takahashi Y., Kanaya T., Takakura I., Hondo T., Chao G., Rose M. T., Ohwada S., Watanabe K., Yamaguchi T., Aso H. (2010). Peripheral serotonin enhances lipid metabolism by accelerating bile
acid turnover. Endocrinology.

[350] Regmi S. C., Park S. Y., Ku S. K., Kim J. A. (2014). Serotonin
regulates innate immune responses of colon epithelial cells through
Nox2-derived reactive oxygen species. Free Rad.
Biol. Med..

[351] Cloez-Tayarani I., Petit-Bertron A. F., Venters H. D., Cavaillon J. M. (2003). Differential
effect of serotonin on cytokine production in lipopolysaccharide-stimulated
human peripheral blood mononuclear cells: involvement of 5-hydroxytryptamine2A
receptors. Int. Immunol..

[352] Bosakova V., Papatheodorou I., Kafka F., Tomasikova Z., Kolovos P., Hortova
Kohoutkova M., Fric J. (2025). Serotonin attenuates
tumor necrosis factor-induced intestinal inflammation by interacting
with human mucosal tissue. Exp. Mol. Med..

[353] Crowell M. D. (2004). Role of serotonin in the pathophysiology
of the irritable
bowel syndrome. Br. J. Pharmacol..

[354] Gros M., Gros B., Mesonero J. E., Latorre E. (2021). Neurotransmitter
Dysfunction in Irritable Bowel Syndrome: Emerging Approaches for Management. J. Clin. Med..

[355] Yusuf S., Al-Saady N., Camm A. J. (2003). 5-hydroxytryptamine
and atrial fibrillation: how significant is this piece in the puzzle?. J. Cardiovasc. Electrophysiol..

[356] Jones B. J., Blackburn T. P. (2002). The medical
benefit of 5-HT research. Pharmacol., Biochem.
Behav..

[357] Ramage A. G. (2001). Central
cardiovascular regulation and 5-hydroxytryptamine
receptors. Brain Res. Bull..

[358] Cote F., Fligny C., Fromes Y., Mallet J., Vodjdani G. (2004). Recent advances in understanding
serotonin regulation
of cardiovascular function. Trends Mol. Med..

[359] Jian B., Xu J., Connolly J., Savani R. C., Narula N., Liang B., Levy R. J. (2002). Serotonin
mechanisms
in heart valve disease I: serotonin-induced up-regulation of transforming
growth factor-beta1 via G-protein signal transduction in aortic valve
interstitial cells. Am. J. Pathol..

[360] Chen C., Han X., Fan F., Liu Y., Wang T., Wang J., Hu P., Ma A., Tian H. (2014). Serotonin drives the activation of pulmonary artery adventitial fibroblasts
and TGF-beta1/Smad3-mediated fibrotic responses through 5-HT­(2A) receptors. Mol. Cell. Biochem..

[361] Nilsson T., Longmore J., Shaw D., Pantev E., Bard J. A., Branchek T., Edvinsson L. (1999). Characterisation
of 5-HT receptors in human coronary arteries by molecular and pharmacological
techniques. Eur. J. Pharmacol..

[362] Yildiz O., Smith J. R., Purdy R. E. (1998). Serotonin
and vasoconstrictor
synergism. Life Sci..

[363] Pakala R., Willerson J. T., Benedict C. R. (1997). Effect of serotonin,
thromboxane A2, and specific receptor antagonists on vascular smooth
muscle cell proliferation. Circulation.

[364] Kwon D., Kohar Y., Stafford N., Oceandy D. (2017). 176 Serotonin
receptor 2b (5-ht2b) modulates cardiomyocyte proliferation by regulating
the hippo pathway. Heart.

[365] Birkeland J. A., Swift F., Tovsrud N., Enger U., Lunde P. K., Qvigstad E., Levy F. O., Sejersted O. M., Sjaastad I. (2007). Serotonin increases L-type Ca2+ current
and SR Ca2+
content through 5-HT4 receptors in failing rat ventricular cardiomyocytes. Am. J. Physiol. Heart Circ. Physiol..

[366] Qvigstad E., Brattelid T., Sjaastad I., Andressen K. W., Krobert K. A., Birkeland J. A., Sejersted O. M., Kaumann A. J., Skomedal T., Osnes J. -B. (2005). Appearance
of a ventricular 5-HT4 receptor-mediated inotropic response to serotonin
in heart failure. Cardiovasc. Res..

[367] Kaumann A. J. (2013). Surprises from a cardiac 5-HT4 TG
mouse: spontaneous
atrial arrhythmias by endogenous 5-HT of atrial origin? Different
mechanism of arrhythmias through 5-HT4 receptors and β-adrenoceptors?. Naunyn Schmiedebergs Arch Pharmacol..

[368] Selim A. M., Sarswat N., Kelesidis I., Iqbal M., Chandra R., Zolty R. (2017). Plasma Serotonin in
Heart Failure: Possible Marker and Potential Treatment Target. Heart Lung Circ..

[369] Sarswat N., Chandra R., Vittorio T. J., Zolty R. (2009). Elevated Serotonin
Levels in Patients with Systolic Heart Failure. J. Card. Failure.

[370] Vikenes K., Farstad M., Nordrehaug J. E. (1999). Serotonin
Is Associated with Coronary Artery Disease and Cardiac Events. Circulation.

[371] Celada P., Puig M. V., Artigas F. (2013). Serotonin modulation
of cortical neurons and networks. Front. Integr.
Neurosci..

[372] Daubert E. A., Condron B. G. (2010). Serotonin: a regulator of neuronal
morphology and circuitry. Trends Neurosci..

[373] Kawashima T. (2018). The role of the serotonergic system
in motor control. Neurosci. Res..

[374] Berger M., Gray J. A., Roth B. L. (2009). The expanded
biology
of serotonin. Annu. Rev. Med..

[375] Hoyer D., Schoeffter P. (1991). 5-HT receptors:
subtypes and second
messengers. J. Recept Res..

[376] Wang X., Wang K., Wu X., Huang W., Yang L. (2022). Role of the cAMP-PKA-CREB-BDNF pathway
in abnormal behaviours of
serotonin transporter knockout mice. Behav Brain
Res..

[377] Ciranna L. (2006). Serotonin as a modulator of glutamate-
and GABA-mediated
neurotransmission: implications in physiological functions and in
pathology. Curr. Neuropharmacol..

[378] Xie G., Zuo W., Wu L., Li W., Wu W., Bekker A., Ye J. -H. (2016). Serotonin modulates
glutamatergic
transmission to neurons in the lateral habenula. Sci. Rep..

[379] Hulsey D. R., Shedd C. M., Sarker S. F., Kilgard M. P., Hays S. A. (2019). Norepinephrine and serotonin are
required for vagus
nerve stimulation directed cortical plasticity. Exp. Neurol..

[380] Airan R. D., Meltzer L. A., Roy M., Gong Y., Chen H., Deisseroth K. (2007). High-speed imaging reveals neurophysiological
links to behavior in an animal model of depression. Science.

[381] Canli T., Lesch K. P. (2007). Long story short: the serotonin transporter
in emotion regulation and social cognition. Nat. Neurosci..

[382] Roth B. L., Hanizavareh S. M., Blum A. E. (2004). Serotonin receptors
represent highly favorable molecular targets for cognitive enhancement
in schizophrenia and other disorders. Psychopharmacology.

[383] Carhart-Harris R. L., Nutt D. J. (2017). Serotonin and brain
function: a tale
of two receptors. J. Psychopharmacol..

[384] Tosini G., Ye K., Iuvone P. M. (2012). N-acetylserotonin:
neuroprotection, neurogenesis, and the sleepy brain. Neuroscientist.

[385] Wadas B., Borjigin J., Huang Z., Oh J. H., Hwang C. S., Varshavsky A. (2016). Degradation of Serotonin N-Acetyltransferase,
a Circadian Regulator, by the N-end Rule Pathway. J. Biol. Chem..

[386] Jang S. W., Liu X., Pradoldej S., Tosini G., Chang Q., Iuvone P. M., Ye K. (2010). N-acetylserotonin
activates TrkB receptor in a circadian rhythm. Proc. Natl. Acad. Sci. U. S. A..

[387] Sompol P., Liu X., Baba K., Paul K. N., Tosini G., Iuvone P. M., Ye K. (2011). *N*-acetylserotonin
promotes hippocampal neuroprogenitor cell proliferation in sleep-deprived
mice. Proc. Natl. Acad. Sci. U. S. A..

[388] Zhou H., Wang J., Jiang J., Stavrovskaya I. G., Li M., Li W., Wu Q., Zhang X., Luo C., Zhou S., Sirianni A. C., Sarkar S., Kristal B. S., Friedlander R. M., Wang X. (2014). *N*-Acetyl-Serotonin
Offers Neuroprotection through Inhibiting Mitochondrial Death Pathways
and Autophagic Activation in Experimental Models of Ischemic Injury. J. Neurosci..

[389] Liu T., Borjigin J. (2005). N-acetyltransferase
is not the rate-limiting enzyme
of melatonin synthesis at night. J. Pineal Res..

[390] Owino S., Contreras-Alcantara S., Baba K., Tosini G. (2016). Melatonin
Signaling Controls the Daily Rhythm in Blood Glucose Levels Independent
of Peripheral Clocks. PLoS One.

[391] Suofu Y., Li W., Jean-Alphonse F. G., Jia J., Khattar N. K., Li J., Baranov S. V., Leronni D., Mihalik A. C., He Y. (2017). Dual role of mitochondria
in producing melatonin and driving GPCR signaling to block cytochrome
c release. Proc. Natl. Acad. Sci. U. S. A..

[392] Tuomi T., Nagorny C. L. F., Singh P., Bennet H., Yu Q., Alenkvist I., Isomaa B., Ostman B., Soderstrom J., Pesonen A. K., Martikainen S., Raikkonen K., Forsen T., Hakaste L., Almgren P., Storm P., Asplund O., Shcherbina L., Fex M., Fadista J., Tengholm A., Wierup N., Groop L., Mulder H. (2016). Increased
Melatonin Signaling Is a Risk Factor for Type 2 Diabetes. Cell Metab..

[393] Ekmekcioglu C. (2006). Melatonin receptors in humans: biological role and
clinical relevance. Biomed. Pharmacother..

[394] Morgan P. J., Barrett P., Howell H. E., Helliwell R. (1994). Melatonin
receptors: localization, molecular pharmacology and physiological
significance. Neurochem. Int..

[395] Liu J., Clough S. J., Hutchinson A. J., Adamah-Biassi E. B., Popovska-Gorevski M., Dubocovich M. L. (2016). MT1 and
MT2Melatonin Receptors: A
Therapeutic Perspective. Annu. Rev. Pharmacol.
Toxicol..

[396] Jockers R., Maurice P., Boutin J. A., Delagrange P. (2008). Melatonin
receptors, heterodimerization, signal transduction and binding sites:
what’s new?. Br. J. Pharmacol..

[397] Slominski R. M., Reiter R. J., Schlabritz-Loutsevitch N., Ostrom R. S., Slominski A. T. (2012). Melatonin membrane receptors in peripheral
tissues: distribution and functions. Mol. Cell.
Endocrinol..

[398] Karasek M., Winczyk K. (2006). Melatonin in humans. J. Physiol.
Pharmacol..

[399] Arangino S., Cagnacci A., Angiolucci M., Vacca A. M., Longu G., Volpe A., Melis G. B. (1999). Effects
of melatonin on vascular reactivity, catecholamine levels, and blood
pressure in healthy men. Am. J. Cardiol..

[400] Cagnacci A., Arangino S., Angiolucci M., Maschio E., Melis G. B. (1998). Influences of melatonin administration
on the circulation of women. Am. J. Physiol..

[401] Doolen S., Krause D. N., Dubocovich M. L., Duckles S. P. (1998). Melatonin mediates two distinct responses in vascular
smooth muscle. Eur. J. Pharmacol..

[402] Iuvone P. M., Tosini G., Pozdeyev N., Haque R., Klein D. C., Chaurasia S. S. (2005). Circadian
clocks, clock networks,
arylalkylamine N-acetyltransferase, and melatonin in the retina. Prog. Retin. Eye Res..

[403] Nishiyama K., Yasue H., Moriyama Y., Tsunoda R., Ogawa H., Yoshimura M., Kugiyama K. (2001). Acute effects of melatonin
administration on cardiovascular autonomic regulation in healthy men. Am. Heart J..

[404] Scheer F. A., Van Montfrans G. A., van Someren E. J., Mairuhu G., Buijs R. M. (2004). Daily nighttime melatonin reduces
blood pressure in male patients with essential hypertension. Hypertension.

[405] Carrillo-Vico A., Guerrero J. M., Lardone P. J., Reiter R. J. (2005). A review
of the multiple actions of melatonin on the immune system. Endocrine.

[406] Cecon E., Oishi A., Jockers R. (2018). Melatonin receptors:
molecular pharmacology and signalling in the context of system bias. Br. J. Pharmacol..

[407] Feybesse C., Chokron S., Tordjman S. (2023). Melatonin in Neurodevelopmental
Disorders: A Critical Literature Review. Antioxidants.

[408] Hohor S., Mandanach C., Maftei A., Zugravu C. A., Otelea M. R. (2023). Impaired Melatonin Secretion, Oxidative Stress and
Metabolic Syndrome in Night Shift Work. Antioxidants.

[409] Pagan C., Delorme R., Callebert J., Goubran-Botros H., Amsellem F., Drouot X., Boudebesse C., Le Dudal K., Ngo-Nguyen N., Laouamri H., Gillberg C., Leboyer M., Bourgeron T., Launay J. M. (2014). The serotonin-N-acetylserotonin-melatonin
pathway as a biomarker for autism spectrum disorders. Transl. Psychiatry.

[410] Ewang-Emukowhate M., Nair D., Caplin M. (2019). The Role of 5-Hydroxyindoleacetic
Acid in Neuroendocrine Tumors: The Journey So Far. Int. J. Endocr. Oncol..

[411] De Giovanni M., Tam H., Valet C., Xu Y., Looney M. R., Cyster J. G. (2022). GPR35 promotes neutrophil recruitment
in response to serotonin metabolite 5-HIAA. Cell.

[412] Joy T., Walsh G., Tokmakejian S., Van Uum S. H. (2008). Increase of urinary
5-hydroxyindoleacetic acid excretion but not serum chromogranin A
following over-the-counter 5-hydroxytryptophan intake. Can. J. Gastroenterol..

[413] Tanaka T., Mori M., Sekino M., Higashijima U., Takaki M., Yamashita Y., Kakiuchi S., Tashiro M., Morimoto K., Tasaki O. (2021). Impact of plasma 5-hydroxyindoleacetic
acid, a serotonin metabolite, on clinical outcome in septic shock,
and its effect on vascular permeability. Sci.
Rep..

[414] Taleb S. (2019). Tryptophan Dietary Impacts Gut Barrier
and Metabolic Diseases. Front. Immunol..

[415] Lee Y. -S., Kim T. -Y., Kim Y., Lee S. -H., Kim S., Kang S. W., Yang J. -Y., Baek I. -J., Sung Y. H., Park Y. -Y. (2018). Microbiota-Derived Lactate Accelerates Intestinal
Stem-Cell-Mediated Epithelial Development. Cell
Host Microbe.

[416] Su X., Gao Y., Yang R. (2022). Gut Microbiota-Derived Tryptophan
Metabolites Maintain Gut and Systemic Homeostasis. Cells.

[417] Zarkan A., Cano-Muniz S., Zhu J., Nahas K. A., Cama J., Keyser U. F., Summers D. K. (2019). Indole Pulse Signalling
Regulates the Cytoplasmic pH of E. coli in a Memory-Like Manner. Sci. Rep..

[418] Cui B., Chen X., Guo Q., Song S., Wang M., Liu J., Deng Y. (2022). The Cell-Cell
Communication Signal Indole Controls
the Physiology and Interspecies Communication of Acinetobacter baumannii. Microbiol. Spectrum..

[419] Kim J., Park W. (2015). Indole: a signaling molecule or a
mere metabolic byproduct
that alters bacterial physiology at a high concentration?. J. Microbiol..

[420] Hubbard T. D., Murray I. A., Bisson W. H., Lahoti T. S., Gowda K., Amin S. G., Patterson A. D., Perdew G. H. (2015). Adaptation of the human aryl hydrocarbon receptor to
sense microbiota-derived indoles. Sci. Rep..

[421] Vyhlidalova B., Krasulova K., Pecinkova P., Marcalikova A., Vrzal R., Zemankova L., Vanco J., Travnicek Z., Vondracek J., Karasova M. (2020). Gut Microbial Catabolites of Tryptophan Are
Ligands and Agonists of the Aryl Hydrocarbon Receptor: A Detailed
Characterization. Int. J. Mol. Sci..

[422] Illes P., Krasulova K., Vyhlidalova B., Poulikova K., Marcalikova A., Pecinkova P., Sirotova N., Vrzal R., Mani S., Dvorak Z. (2020). Indole microbial
intestinal metabolites expand the repertoire of ligands and agonists
of the human pregnane X receptor. Toxicol. Lett..

[423] Venkatesh M., Mukherjee S., Wang H., Li H., Sun K., Benechet A. P., Qiu Z., Maher L., Redinbo M. R., Phillips R. S., Fleet J. C., Kortagere S., Mukherjee P., Fasano A., Le Ven J., Nicholson J. K., Dumas M. E., Khanna K. M., Mani S. (2014). Symbiotic
bacterial
metabolites regulate gastrointestinal barrier function via the xenobiotic
sensor PXR and Toll-like receptor 4. Immunity.

[424] Shimada Y., Kinoshita M., Harada K., Mizutani M., Masahata K., Kayama H., Takeda K. (2013). Commensal bacteria-dependent
indole production enhances epithelial barrier function in the colon. PLoS One.

[425] Gillam E. M. J., Notley L. M., Cai H., De Voss J. J., Guengerich F. P. (2000). Oxidation of Indole by Cytochrome P450 Enzymes. Biochemistry.

[426] Banoglu E., Jha G., King R. (2001). Hepatic microsomal
metabolism of indole to indoxyl, a precursor of indoxyl sulfate. Eur. J. Drug Metab. Pharmacokinet.

[427] Banoglu E., King R. S. (2002). Sulfation of indoxyl
by human and
rat aryl (phenol) sulfotransferases to form indoxyl sulfate. Eur. J. Drug Metab. Pharmacokinet.

[428] Deguchi T., Ohtsuki S., Otagiri M., Takanaga H., Asaba H., Mori S., Terasaki T. (2002). Major role
of organic
anion transporter 3 in the transport of indoxyl sulfate in the kidney. Kidney Int..

[429] Schroeder J. C., Dinatale B. C., Murray I. A., Flaveny C. A., Liu Q., Laurenzana E. M., Lin J. M., Strom S. C., Omiecinski C. J., Amin S., Perdew G. H. (2010). The uremic toxin
3-indoxyl sulfate is a potent endogenous agonist for the human aryl
hydrocarbon receptor. Biochemistry.

[430] Ito S., Osaka M., Edamatsu T., Itoh Y., Yoshida M. (2016). Crucial Role
of the Aryl Hydrocarbon Receptor (AhR) in Indoxyl Sulfate-Induced
Vascular Inflammation. J. Atheroscler Thromb..

[431] Nakano T., Katsuki S., Chen M., Decano J. L., Halu A., Lee L. H., Pestana D. V. S., Kum A. S. T., Kuromoto R. K., Golden W. S., Boff M. S., Guimaraes G. C., Higashi H., Kauffman K. J., Maejima T., Suzuki T., Iwata H., Barabási A. L., Aster J. C., Anderson D. G., Sharma A., Singh S. A., Aikawa E., Aikawa M. (2019). Uremic Toxin
Indoxyl Sulfate Promotes Proinflammatory Macrophage Activation Via
the Interplay of OATP2B1 and Dll4-Notch Signaling. Circulation.

[432] Shimizu H., Hirose Y., Goto S., Nishijima F., Zrelli H., Zghonda N., Niwa T., Miyazaki H. (2012). Indoxyl sulfate
enhances angiotensin II signaling through upregulation of epidermal
growth factor receptor expression in vascular smooth muscle cells. Life Sci..

[433] Yang J., Li H., Zhang C., Zhou Y. (2022). Indoxyl sulfate
reduces Ito,f by activating ROS/MAPK and NF-kappaB signaling pathways. JCI Insight.

[434] Sato A., Soeno K., Kikuchi R., Narukawa-Nara M., Yamazaki C., Kakei Y., Nakamura A., Shimada Y. (2022). Indole-3-pyruvic
acid regulates TAA1 activity, which plays a key role in coordinating
the two steps of auxin biosynthesis. Proc. Natl.
Acad. Sci. U. S. A..

[435] Bittinger M. A., Nguyen L. P., Bradfield C. A. (2003). Aspartate
aminotransferase generates proagonists of the aryl hydrocarbon receptor. Mol. Pharmacol..

[436] Chowdhury G., Dostalek M., Hsu E. L., Nguyen L. P., Stec D. F., Bradfield C. A., Guengerich F. P. (2009). Structural
identification of Diindole agonists of the aryl hydrocarbon receptor
derived from degradation of indole-3-pyruvic acid. Chem. Res. Toxicol..

[437] Nguyen L. P., Hsu E. L., Chowdhury G., Dostalek M., Guengerich F. P., Bradfield C. A. (2009). D-amino
acid oxidase generates agonists of the aryl hydrocarbon receptor from
D-tryptophan. Chem. Res. Toxicol..

[438] Aoki R., Aoki-Yoshida A., Suzuki C., Takayama Y. (2018). Indole-3-Pyruvic
Acid, an Aryl Hydrocarbon Receptor Activator, Suppresses Experimental
Colitis in Mice. J. Immunol..

[439] McGettrick A. F., Corcoran S. E., Barry P. J., McFarland J., Cres C., Curtis A. M., Franklin E., Corr S. C., Mok K. H., Cummins E. P. (2016). Trypanosoma
brucei metabolite
indolepyruvate decreases HIF-1alpha and glycolysis in macrophages
as a mechanism of innate immune evasion. Proc.
Natl. Acad. Sci. U. S. A..

[440] Meng D., Sommella E., Salviati E., Campiglia P., Ganguli K., Djebali K., Zhu W., Walker W. A. (2020). Indole-3-lactic
acid, a metabolite of tryptophan, secreted by Bifidobacterium longum
subspecies infantis is anti-inflammatory in the immature intestine. Pediatr. Res..

[441] Wong C. B., Tanaka A., Kuhara T., Xiao J. Z. (2020). Potential
Effects of Indole-3-Lactic Acid, a Metabolite of Human Bifidobacteria,
on NGF-induced Neurite Outgrowth in PC12 Cells. Microorganisms.

[442] Qian X., Li Q., Zhu H., Chen Y., Lin G., Zhang H., Chen W., Wang G., Tian P. (2024). Bifidobacteria
with indole-3-lactic acid-producing capacity exhibit psychobiotic
potential via reducing neuroinflammation. Cell
Rep. Med..

[443] Kim H., Lee E., Park M., Min K., Diep Y. N., Kim J., Ahn H., Lee E., Kim S., Kim Y., Kang Y. J., Jung J. H., Byun M. S., Joo Y., Jeong C., Lee D. Y., Cho H., Park H., Kim T. (2024). Microbiome-derived indole-3-lactic acid reduces amyloidopathy through
aryl-hydrocarbon receptor activation. Brain.
Behav. Immun..

[444] Wlodarska M., Luo C., Kolde R., d’Hennezel E., Annand J. W., Heim C. E., Krastel P., Schmitt E. K., Omar A. S., Creasey E. A. (2017). Indoleacrylic Acid Produced
by Commensal Peptostreptococcus Species Suppresses Inflammation. Cell Host Microbe..

[445] Nault R., Doskey C. M., Fader K. A., Rockwell C. E., Zacharewski T. (2018). Comparison of Hepatic NRF2 and Aryl Hydrocarbon Receptor
Binding in 2,3,7,8-Tetrachlorodibenzo-p-dioxin-Treated Mice Demonstrates
NRF2-Independent PKM2 Induction. Mol. Pharmacol..

[446] Wakabayashi N., Slocum S. L., Skoko J. J., Shin S., Kensler T. W. (2010). When NRF2
talks, who’s listening?. Antioxid. Redox
Signal..

[447] Dodd D., Spitzer M. H., Van Treuren W., Merrill B. D., Hryckowian A. J., Higginbottom S. K., Le A., Cowan T. M., Nolan G. P., Fischbach M. A., Sonnenburg J. L. (2017). A gut bacterial pathway metabolizes
aromatic amino
acids into nine circulating metabolites. Nature.

[448] Chyan Y. J., Poeggeler B., Omar R. A., Chain D. G., Frangione B., Ghiso J., Pappolla M. A. (1999). Potent neuroprotective
properties against the Alzheimer beta-amyloid by an endogenous melatonin-related
indole structure, indole-3-propionic acid. J.
Biol. Chem..

[449] Pryor W. A. (1986). Oxy-radicals
and related species: their formation,
lifetimes, and reactions. Annu. Rev. Physiol..

[450] Li Q., de Oliveira Formiga R., Puchois V., Creusot L., Ahmad A. H., Amouyal S., Campos-Ribeiro M. A., Zhao Y., Harris D. M. M., Lasserre F. (2025). Microbial
metabolite indole-3-propionic acid drives mitochondrial respiration
in CD4­(+) T cells to confer protection against intestinal inflammation. Nat. Metab..

[451] Zhao Z. H., Xin F. Z., Xue Y., Hu Z., Han Y., Ma F., Zhou D., Liu X. L., Cui A., Liu Z., Liu Y., Gao J., Pan Q., Li Y., Fan J. G. (2019). Indole-3-propionic acid inhibits gut dysbiosis and
endotoxin leakage to attenuate steatohepatitis in rats. Exp. Mol. Med..

[452] Xue H., Chen X., Yu C., Deng Y., Zhang Y., Chen S., Chen X., Chen K., Yang Y., Ling W. (2022). Gut Microbially Produced Indole-3-Propionic
Acid Inhibits Atherosclerosis
by Promoting Reverse Cholesterol Transport and Its Deficiency Is Causally
Related to Atherosclerotic Cardiovascular Disease. Circ. Res..

[453] Zhang B., Jiang M., Zhao J., Song Y., Du W., Shi J. (2022). The Mechanism Underlying the Influence of Indole-3-Propionic
Acid: A Relevance to Metabolic Disorders. Front.
Endocrinol..

[454] Zeitler L., Murray P. J. (2023). IL4i1 and IDO1:
Oxidases that control
a tryptophan metabolic nexus in cancer. J. Biol.
Chem..

[455] Sadik A., Somarribas Patterson L. F., Ozturk S., Mohapatra S. R., Panitz V., Secker P. F., Pfander P., Loth S., Salem H., Prentzell M. T. (2020). IL4I1 Is a Metabolic Immune Checkpoint that Activates the AHR and
Promotes Tumor Progression. Cell.

[456] Boulland M. L., Marquet J., Molinier-Frenkel V., M?ller P., Guiter C., Lasoudris F., Copie-Bergman C., Baia M., Gaulard P., Leroy K. (2007). Human IL4I1 is a secreted L-phenylalanine oxidase expressed by mature
dendritic cells that inhibits T-lymphocyte proliferation. Blood.

[457] Williams B. B., Van Benschoten A. H., Cimermancic P., Donia M. S., Zimmermann M., Taketani M., Ishihara A., Kashyap P. C., Fraser J. S., Fischbach M. A. (2014). Discovery
and characterization of gut microbiota decarboxylases that can produce
the neurotransmitter tryptamine. Cell Host Microbe..

[458] Hou Y., Li J., Ying S. (2023). Tryptophan
Metabolism and Gut Microbiota:
A Novel Regulatory Axis Integrating the Microbiome, Immunity, and
Cancer. Metabolites.

[459] Bhattarai Y., Williams B. B., Battaglioli E. J., Whitaker W. R., Till L., Grover M., Linden D. R., Akiba Y., Kandimalla K. K., Zachos N. C. (2018). Gut
Microbiota-Produced Tryptamine Activates an Epithelial G-Protein-Coupled
Receptor to Increase Colonic Secretion. Cell
Host Microbe.

[460] Benech N., Rolhion N., Sokol H. (2021). Tryptophan metabolites
get the gut moving. Cell Host Microbe.

[461] Marien M. R., Gerber R., Boyar W. C., Altar C. A. (1987). Injections
of deuterated tryptamine into the nucleus accumbens of the rat: effects
on locomotor activity and monoamine metabolism. Neuropharmacology.

[462] Leonard B. E., Shallice S. A. (1972). The effects of some tryptamine derivatives
on brain monoamines and their precursor amino acids. Neuropharmacology.

[463] Herrera F., Martin V., Carrera P., Garcia-Santos G., Rodriguez-Blanco J., Rodriguez C., Antolin I. (2006). Tryptamine induces
cell death with ultrastructural features of autophagy in neurons and
glia: Possible relevance for neurodegenerative disorders. Anat Rec A Discov Mol. Cell Evol Biol..

[464] Jones R. S. (1982). Tryptamine: a neuromodulator or neurotransmitter in
mammalian brain?. Prog. Neurobiol..

[465] Yu A. M., Granvil C. P., Haining R. L., Krausz K. W., Corchero J., Küpfer A., Idle J. R., Gonzalez F. J. (2003). The relative
contribution of monoamine oxidase and cytochrome p450 isozymes to
the metabolic deamination of the trace amine tryptamine. J. Pharmacol. Exp. Ther..

[466] Bunsangiam S., Sakpuntoon V., Srisuk N., Ohashi T., Fujiyama K., Limtong S. (2019). Biosynthetic
Pathway of Indole-3-Acetic
Acid in Basidiomycetous Yeast Rhodosporidiobolus fluvialis. Mycobiology.

[467] Bar T., Okon Y. (1993). Tryptophan Conversion to Indole-3-Acetic-Acid
Via Indole-3-Acetamide
in Azospirillum-Brasilense Sp7. Can. J. Microbiol..

[468] Malhotra M., Srivastava S. (2008). Organization
of the ipdC region regulates
IAA levels in different Azospirillum brasilense strains: molecular
and functional analysis of ipdC in strain SM. Environ. Microbiol..

[469] Dai X., Mashiguchi K., Chen Q., Kasahara H., Kamiya Y., Ojha S., DuBois J., Ballou D., Zhao Y. (2013). The biochemical
mechanism of auxin biosynthesis by an arabidopsis YUCCA flavin-containing
monooxygenase. J. Biol. Chem..

[470] Shah A., Mathur Y., Hazra A. B. (2021). Double
agent indole-3-acetic
acid: mechanistic analysis of indole-3-acetaldehyde dehydrogenase
AldA that synthesizes IAA, an auxin that aids bacterial virulence. Biosci. Rep..

[471] Shaheen N., Miao J., Xia B., Zhao Y., Zhao J. (2025). Multifaceted Role of Microbiota-Derived
Indole-3-Acetic Acid in Human
Diseases and Its Potential Clinical Application. FASEB J..

[472] Addi T., Poitevin S., McKay N., El Mecherfi K. E., Kheroua O., Jourde-Chiche N., de Macedo A., Gondouin B., Cerini C., Brunet P., Dignat-George F., Burtey S., Dou L. (2019). Mechanisms of tissue
factor induction
by the uremic toxin indole-3 acetic acid through aryl hydrocarbon
receptor/nuclear factor-kappa B signaling pathway in human endothelial
cells. Arch. Toxicol..

[473] Dou L., Sallee M., Cerini C., Poitevin S., Gondouin B., Jourde-Chiche N., Fallague K., Brunet P., Calaf R., Dussol B., Mallet B., Dignat-George F., Burtey S. (2015). The cardiovascular
effect of the uremic solute indole-3
acetic acid. J. Am. Soc. Nephrol..

[474] Motojima M., Hosokawa A., Yamato H., Muraki T., Yoshioka T. (2003). Uremic toxins of organic anions up-regulate
PAI-1 expression
by induction of NF-kappaB and free radical in proximal tubular cells. Kidney Int..

[475] Cheng J., Wang N. X., Yu L., Luo Y. M., Liu A. K., Tang S., Xu J. Y., Wang Y. S., Zhu J. P., Lebedev A., Tian C. L., Tan R. X. (2024). Molecular
Insights into the One-Carbon Loss Oxidation of Indole-3-acetic Acid. ACS Catal..

[476] Zgarbova E., Vrzal R. (2023). Skatole: A thin red line between
its benefits and toxicity. Biochimie.

[477] Zelante T., Puccetti M., Giovagnoli S., Romani L. (2021). Regulation of host physiology and immunity by microbial
indole-3-aldehyde. Curr. Opin. Immunol..

[478] Zelante T., Iannitti R. G., Cunha C., De Luca A., Giovannini G., Pieraccini G., Zecchi R., D’Angelo C., Massi-Benedetti C., Fallarino F., Carvalho A., Puccetti P., Romani L. (2013). Tryptophan
catabolites from microbiota engage aryl
hydrocarbon receptor and balance mucosal reactivity via interleukin-22. Immunity.

[479] Borghi M., Pariano M., Solito V., Puccetti M., Bellet M. M., Stincardini C., Renga G., Vacca C., Sellitto F., Mosci P. (2019). Targeting the Aryl Hydrocarbon
Receptor With Indole-3-Aldehyde Protects From Vulvovaginal Candidiasis
via the IL-22-IL-18 Cross-Talk. Front. Immunol..

[480] Powell D. N., Swimm A., Sonowal R., Bretin A., Gewirtz A. T., Jones R. M., Kalman D. (2020). Indoles from the commensal
microbiota act via the AHR and IL-10 to tune the cellular composition
of the colonic epithelium during aging. Proc.
Natl. Acad. Sci. U. S. A..

[481] Swimm A., Giver C. R., DeFilipp Z., Rangaraju S., Sharma A., Ulezko Antonova A., Sonowal R., Capaldo C., Powell D., Qayed M., Kalman D., Waller E. K. (2018). Indoles
derived from intestinal microbiota act via type I interferon signaling
to limit graft-versus-host disease. Blood.

[482] Wang P., Tao W., Li Q., Ma W., Jia W., Kang Y. (2025). Indole-3-Aldehyde alleviates lung
inflammation in COPD
through activating Aryl Hydrocarbon Receptor to inhibit HDACs/NF-kappaB/NLRP3
signaling pathways. J. Mol. Med..

[483] Cui L., Wang Z., Guo Z., Zhang H., Liu Y., Zhang H., Jin H., Xu F., Wang X., Xie C., Guo H., Wang T., Lin Y. (2025). Tryptophan
Metabolite Indole-3-Aldehyde Induces AhR and c-MYC Degradation to
Promote Tumor Immunogenicity. Adv. Sci..

